# Extending the biocatalytic scope of regiocomplementary flavin-dependent halogenase enzymes†
†Electronic supplementary information (ESI) available. See DOI: 10.1039/c5sc00913h


**DOI:** 10.1039/c5sc00913h

**Published:** 2015-04-10

**Authors:** Sarah A. Shepherd, Chinnan Karthikeyan, Jonathan Latham, Anna-Winona Struck, Mark L. Thompson, Binuraj R. K. Menon, Matthew Q. Styles, Colin Levy, David Leys, Jason Micklefield

**Affiliations:** a School of Chemistry , The University of Manchester , 131 Princess Street , Manchester , M1 7DN , UK . Email: jason.micklefield@manchester.ac.uk; b Manchester Institute of Biotechnology , The University of Manchester , 131 Princess Street , Manchester , M1 7DN , UK; c Faculty of Life Sciences , The University of Manchester , 131 Princess Street , Manchester , M1 7DN , UK

## Abstract

Flavin-dependent halogenases are potentially valuable biocatalysts for the regioselective halogenation of aromatic compounds. These enzymes, utilising benign inorganic halides, offer potential advantages over traditional non-enzymatic halogenation chemistry that often lacks regiocontrol and requires deleterious reagents. Here we extend the biocatalytic repertoire of the tryptophan halogenases, demonstrating how these enzymes can halogenate a range of alternative aryl substrates. Using structure guided mutagenesis we also show that it is possible to alter the regioselectivity as well as increase the activity of the halogenases with non-native substrates including anthranilic acid; an important intermediate in the synthesis and biosynthesis of pharmaceuticals and other valuable products.

## Abstract 2

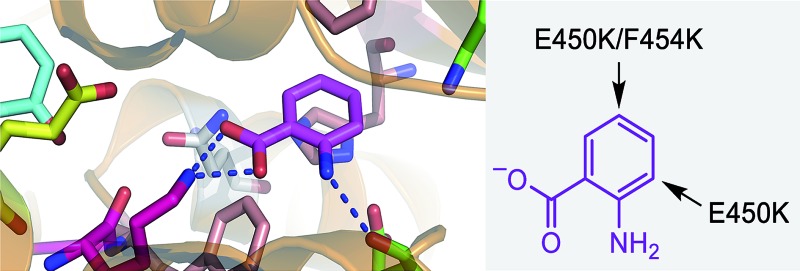
Targeted mutagenesis increases the activity and alters the regioselectivity of flavin-dependent halogenases.

## Introduction

Halogenated aromatic compounds are widely used as pharmaceuticals, agrochemicals, polymers and in other materials.^1^–^3^ Aryl and heteroaryl halides are also key synthetic precursors in many of the most powerful transition metal catalysed cross-coupling reactions which are ubiquitous in synthesis today.^4^–^10^ Despite this, current methods for producing haloaromatic compounds often involve toxic or deleterious reagents, catalysts and solvents. Traditional halogenation methods can also lack regiocontrol rendering some regioisomers inaccessible and resulting in unwanted by-products, including polyhalogenated compounds, that require separation and careful disposal due to their toxicity and/or persistence in the environment.^11^–^15^ The development of regioselective halogenase enzymes, utilising benign inorganic halides to deliver a range of desirable haloaromatic molecules, would present an attractive alternative that may alleviate difficulties encountered with non-enzymatic halogenation chemistry.

**Fig. 1:**
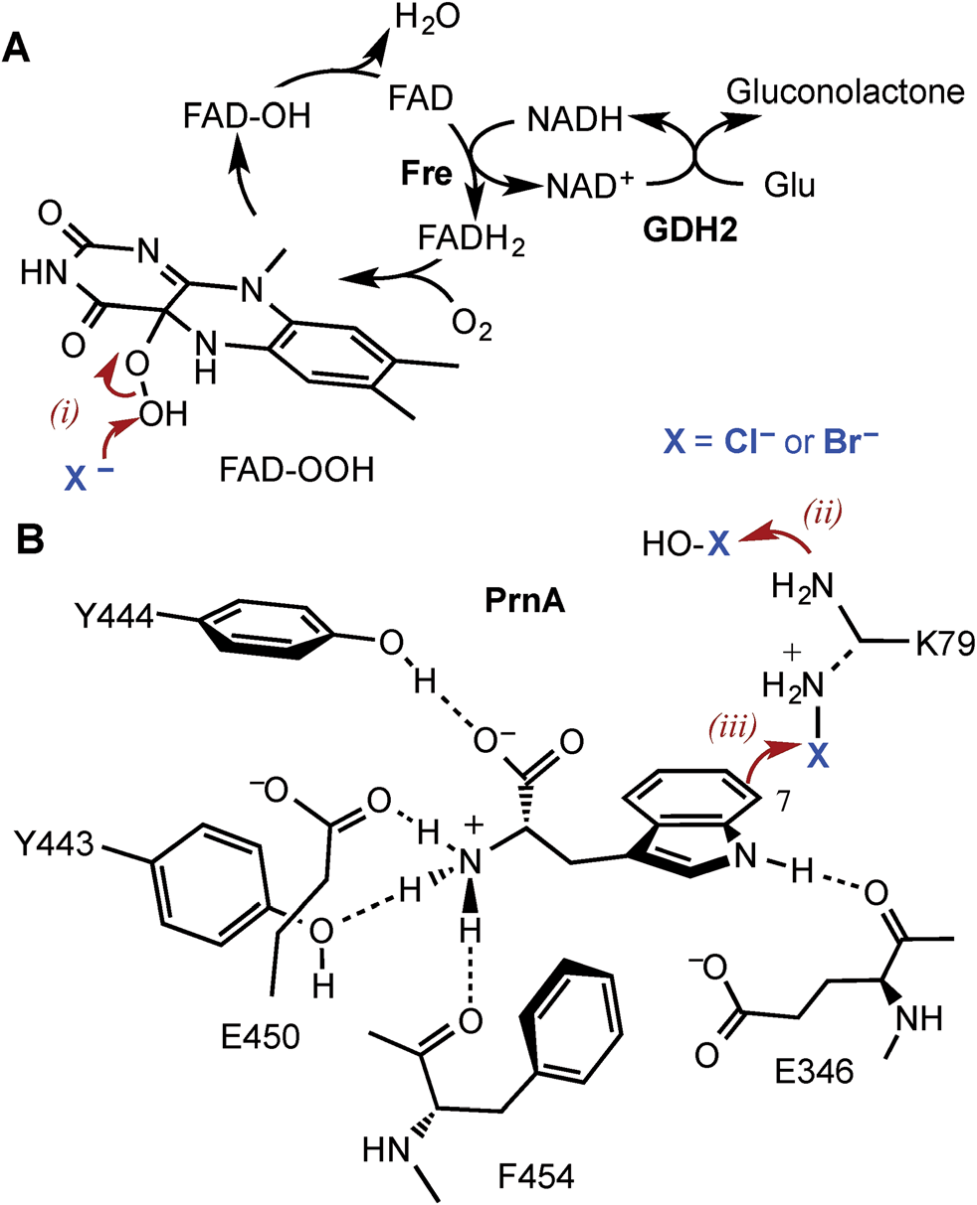
The mechanism of the tryptophan 7-halogenase PrnA. (A) The flavin reductase from *Escherichia coli* (Fre) and glucose dehydrogenase (GDH2) from *Bacillus megaterium* were used to recycle FAD and NAD^+^. (B) PrnA active site with the tryptophan substrate bound. Residues which stack above and below the substrate indole are removed for clarity. The hypohalous acid generated (step i), reacts with the amino group of K79 (step ii) resulting in a chloroamine electrophile which attacks the indole C7 (step iii). E346 acts as a general base to deprotonate the σ-complex.

The heme- and vanadium-dependent haloperoxidases were considered for potential biocatalysis applications, but their development has been hampered by a lack of substrate specificity and regioselectivity, presenting similar issues of by-product separation as those encountered with non-enzymatic halogenations.^16^,^17^ However, in recent years evidence has emerged that it is Fe^2+^/α-ketoglutarate (α-KG) and particularly flavin-dependent halogenases, which are most widely utilised in nature to catalyse regioselective halogenation reactions during the biosynthesis of halogenated natural products.^18^–^21^ Flavin-dependent halogenases require an associated reductase to reduce FAD to FADH_2_, which is then oxidised by the halogenase and O_2_ to give C4a-hydroperoxyflavin (FAD-OOH) (Fig. 1A). It is then suggested that chloride attacks the distal oxygen atom of FAD-OOH to generate hypochlorous acid (HOCl),^22^–^24^ which H-bonds with an active site lysine residue positioning the electrophile in a spatially defined orientation relative to the aromatic substrate.^22^ Alternatively, the active site lysine may react with HOCl to generate an electrophilic chloroamine (Fig. 1B).^24^ Both proposed mechanisms indicate that the lysine residue controls the regiochemistry of the electrophilic attack and the resulting σ-complex is then deprotonated, by an active site general base, to give the halogenated product. All of the flavin-dependent halogenases investigated to date will accept bromide, as well as chloride, to yield either brominated or chlorinated products.^25^

Many of the Fe^2+^/α-KG and flavin-dependent halogenases, process substrates which are tethered to carrier proteins of polyketide synthase or nonribosomal peptide synthetase assembly-line enzymes^26^,^27^ limiting their scope for synthetic applications. On the other hand, the flavin-dependent tryptophan halogenases can regioselectively halogenate free tryptophan which makes them more viable candidates for further development as biocatalysts. Moreover, the tryptophan halogenases characterized to date demonstrate exquisite regio-complementarity and include: PyrH a tryptophan 5-halogenase;^28^ SttH, Thal and KtzR tryptophan 6-halogenases;^29^–^31^ and PrnA, KtzQ and RebH tryptophan 7-halogenases.^31^–^33^ In principle these enzymes would provide a useful starting point from which to develop and evolve more generic regioselective halogenase enzymes. Despite this potential, progress in the development of flavin-dependent halogenases for biocatalysis, has been hampered by limited substrate scope, poor catalytic activity^22^ and enzyme instability.^34^,^35^ In the case of the RebH halogenase, notable recent reports have demonstrated how directed evolution,^34^ or cross-linked enzyme aggregates,^35^ can be used to improve the productivity of halogenase enzymes for synthetic applications. In this paper we describe how Trp halogenases can be engineered, extending their biocatalytic repertoire, to accept a wider range of aryl substrates with improved activity and altered regioselectivities.

## Results and discussion

**Fig. 2:**
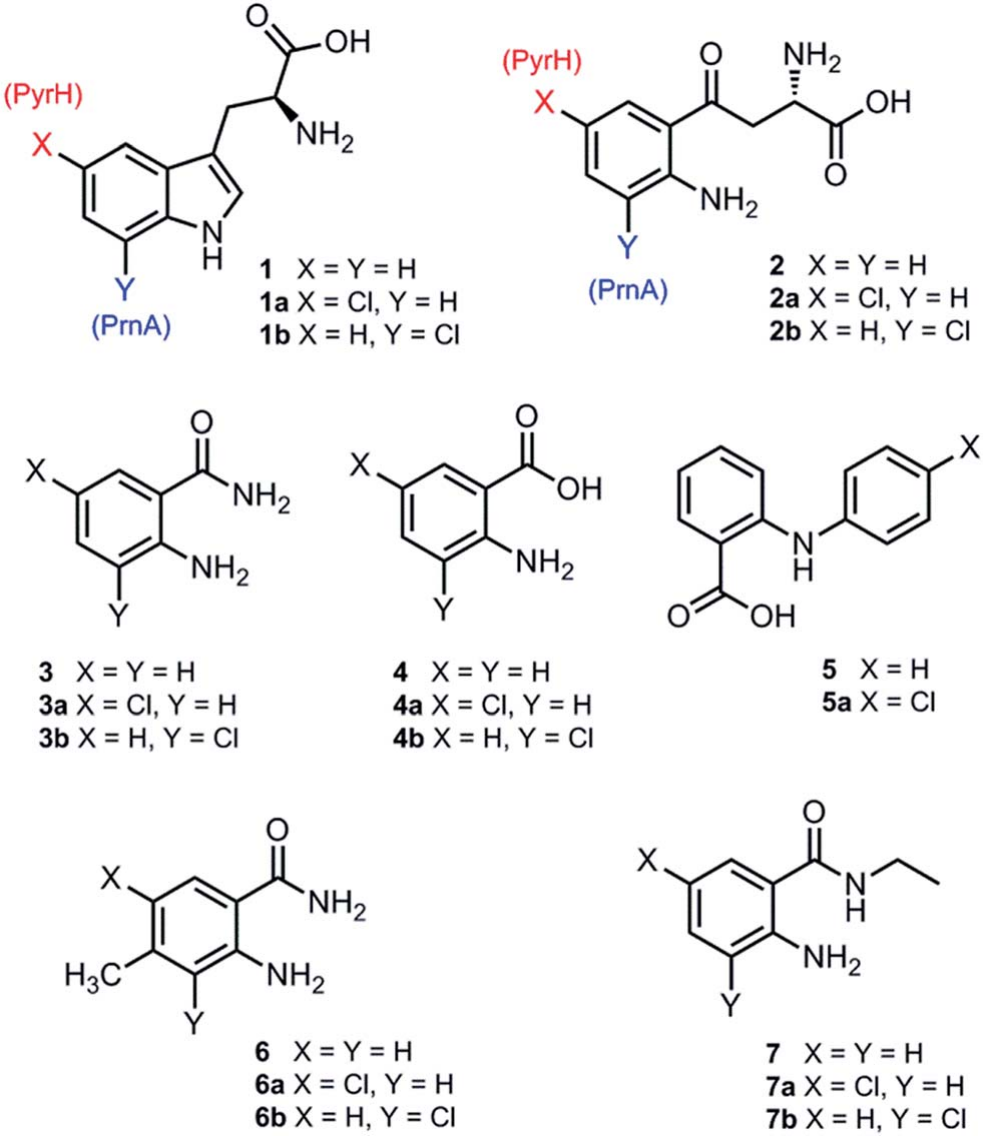
Substrates and products from reactions with PyrH and PrnA.

Although PrnA^36^ and RebH^37^,^38^ have been shown to halogenate indoles as well as Trp (**1**), the substrate specificities of the larger family of Trp halogenases have not been extensively evaluated. Accordingly, the regiocomplementary tryptophan 5- and 7-halogenases PyrH^39^ and PrnA,^22^ were overproduced in *E. coli* and screened against a number of alternative non-indolic substrates including kynurenine (**2**), anthranilamide (**3**), anthranilic acid (**4**) and other anilines (Fig. 2). With the natural substrate tryptophan (**1**), PyrH gave 5-chlorotryptophan (**1a**) and PrnA gave 7-chlorotryptophan (**1b**) exclusively as reported previously.^22^,^28^ With PrnA, the carboxylic acid and the amino group of the Trp substrate are engaged in a series of hydrogen bonds to tyrosine (Y443 and Y444), glutamate (E450) and phenylalanine (F454) residues; the indole moiety is stacked between aromatic amino acids (H101, F103 and W455), with an hydrogen bond between the indole NH and a backbone carbonyl group (E346). These interactions serve to orientate the C7 of the indole towards the key catalytic lysine (K79) and glutamate (E346) residues which are suggested to govern the observed regioselectivity (Fig. 1).^22^

As we move away from the natural substrate to non-indolic molecules the regiocomplementarity is maintained for kynurenine (**2**), with PyrH affording 5-chlorokynurenine (**2a**), whereas PrnA halogenation gives 3-chlorokynurenine (**2b**) (Table 1). Positioning of kynurenine into the active site of PrnA (Fig. S1†), reveals, as anticipated, that **2** is likely to engage in the same hydrogen bonding and π-stacking interactions as the natural substrate (Trp), and an additional contact between the keto group of **2** and an active site asparagine (N459) may also be possible. With the smaller substrate anthranilamide (**3**), both PyrH and PrnA favour halogenation *para* to amino group; PyrH exclusively produced 5-chloroanthranilamide (**3a**), while PrnA formed 5-chloroanthranilamide (**3a**, 86%) with 3-chloro-anthranilamide (**3b**, 14%) as a minor product. Presumably many of the key substrate interactions in the active site of PrnA and PyrH are lost, leading to more flexibility in the binding position of anthranilamide. For example with PrnA π-stacking interactions and contact of the aniline amino group with the backbone carbonyl of E346 may be maintained to allow for C3 (*ortho*) halogenation (Fig. S2A†). However, the NH_2_ of the amide may also participate in hydrogen bonding to E346, which could enable an alternative binding mode, that would position C5 of anthranilamide closest to the key lysine residue (K79) (Fig. S2B†). Anthranilic acid (**4**) proved to be a poorer substrate than anthranilamide for both PyrH and PrnA with conversions of 1% or less for both enzymes (Table 1). Whilst PyrH gave exclusively 5-chloroanthranilate, PrnA gave predominately 3-chloroanthranilate (**4b**, 84%) with 5-chloroanthranilate (**4a**, 16%) the minor product which is notably opposite to the regioselectivity observed for PrnA with anthranilamide.

**Table 1:** Conversions and regioselectivity of PyrH and PrnA with **1–4**

Substrate	Enzyme	Conversion^*a*^	Product^*b*^ (*o*/*p*-ratio)
**1**	PyrH	100%	**1a** (*p*-100%)
	PrnA	59%	**1b** (*o*-100%)
**2**	PyrH	67%	**2a** (*p*-100%)
	PrnA	76%	**2b** (*o*-100%)
**3**	PyrH	46%	**3a** (*p*-100%)
	PrnA	19%	**3a** (*p*-86%), **3b** (*o*-14%)
**4**	PyrH	<1%	**4a** (*p*-100%)
	PrnA	1%	**4a** (*p*-16%), **4b** (*o*-84%)

^*a*^Conversion after 30 minutes with halogenase enzyme (10 μM) and substrate (0.5 mM).

^*b*^Ratios of halogenation *ortho* or *para* to the aryl NH/NH_2_ group was determined by HPLC.

Interestingly, non-enzymatic halogenation of anthranilates using traditional chemistry favours C5 over C3, and can often result in mixtures including C3 and C5 disubstituted products.^40^,^41^ We do not observe any dihalogenation of **3** or **4** with the halogenases. Moreover the fact PrnA predominately halogenates anthranilic acid at C3, whilst the C5 position *para* to the activating amino group is intrinsically the most reactive position,^40^,^41^ suggests that substrate binding still governs regioselectivity. Presumably while anthranilate (**4**) may have greater flexibility in the PrnA active site, compared with the native substrate (Trp), it is preferentially bound in an orientation that would position C3 closer to the catalytic amino and carboxyl groups of K79 and E346 respectively. Several other anilines also proved to be halogenase substrates; *N*-phenylanthranilic acid (**5**) resulted in a single chlorinated product **5a** with both halogenases; 2-amino-4-methylbenzamide (**6**) producing exclusively the 5-chloro derivative (**6a**) with PyrH, whereas PrnA gave a mixture of both the 3- and 5-halogenated products (**6a** and **6b**); PrnA was also able to halogenate 2-amino-*N*-ethylbenzamide (**7**), yielding a mixture of 5- and 3- halogenated derivatives (**7a**) and (**7b**). Although only the chlorination reactions are discussed here, similar regioselectivities were observed for bromination reactions with various alternative non-indolic substrates.

Catalytic parameters were determined for chlorination reactions catalysed by PrnA with selected substrates **1–4** (Table 2). It is clear that although tryptophan binds most tightly to the enzyme, kynurenine exhibits the highest turnover number. This may be due to increased flexibility of the kynurenine aryl group within the active site which could reduce binding affinity, on the one hand, but might also enable the aryl group to adopt a geometry that would lead to a more stable transition state upon attack of the chloroamine electrophile. Smaller substrates anthranilamide (**3**) and anthranilic acid (**4**) exhibit turnover numbers that are similar to the native substrate, but as expected have much higher *K*_m_ values than tryptophan (**1**) or kynurenine (**2**), probably due to reduced potential for hydrogen bonding within the active site of PrnA. Overall from *k*_cat_/*K*_m_ values it is clear that anthranilic acid **4**, is the least efficient substrate with PrnA.

**Table 2:** Kinetics for PrnA wild type and mutants with tryptophan (**1**), kynurenine (**2**), anthranilamide (**3**), and anthranilic acid (**4**) as substrates

Sub	Enzyme	*K*_m_ (μM)	*k*_cat_ (min^–1^)	*k*_cat_/*K*_m_ × 10^–3^ (min^–1^ μM^–1^)
**1**	WT	0.7 ± 0.1	1.1 ± 0.05	1700 ± 200
**2**	WT	19.0 ± 2.2	3.7 ± 0.1	200 ± 10
**3**	WT	3267 ± 491	2.1 ± 0.1	0.7 ± 0.1
**4**	WT	3161 ± 986	0.51 ± 0.07	0.16 ± 0.05
**4**	E450K	384 ± 93	0.93 ± 0.05	2.4 ± 0.6
**4**	F454K	3628 ± 1108	3.66 ± 0.67	1.0 ± 0.4
**4**	E450K, F454K	205 ± 16	1.82 ± 0.04	8.9 ± 0.7
**4**	E450K, F454R	378 ± 59	1.27 ± 0.07	3.4 ± 0.6

Anthranilic acid is a good target for biohalogenation, given that halogenated anthranilic acids are widely used scaffolds in the development of pharmaceuticals, agrochemicals and other materials of industrial importance (for some recent examples see Fig. S3 and references in ESI†). Anthranilic acid is also a key precursor in the biosynthesis of many bioactive natural products.^42^–^44^ Consequently, haloanthranilates, produced *via* fermentation from microbial strains possessing an appropriate engineered halogenase, could be useful precursors for incorporation into biosynthetic pathways leading to new halogenated secondary metabolites.^45^,^46^ In light of this, we sought to engineer PrnA to improve its activity and regioselectivity with anthranilic acid through structure-guided mutagenesis. It seems unlikely that anthranilic acid could contact PrnA active site residues Y443, Y444, E450 and F454, as tryptophan does, while remaining proximal to the catalytic side chains of K79 and E346 (Fig. 1). Consequently, a series of PrnA mutants were produced with selected active site residues mutated to lysine or arginine. It was reasoned that the additional basic amino acid residues might make contact with the carboxylate group of anthranilic acid to enable tighter substrate binding and effect the orientation of anthranilic acid relative to the catalytic active site residues, thus altering the regioselectivity of halogenation.

**Fig. 3:**
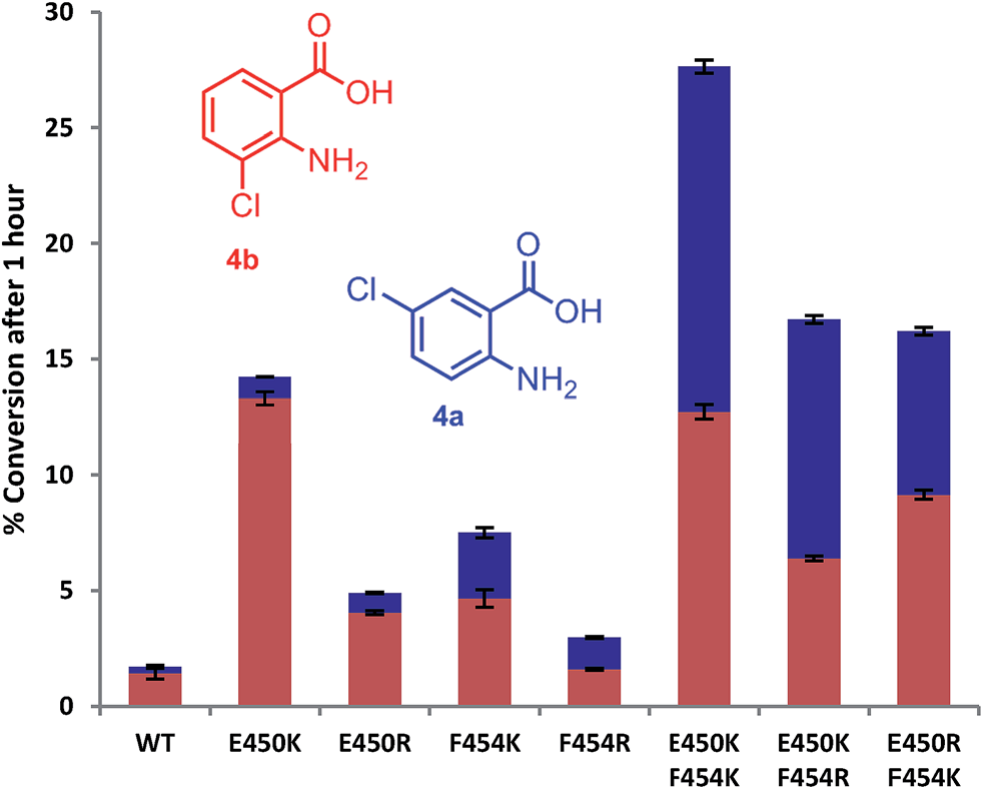
The % conversion of anthranilic **4**, by PrnA mutants, and ratios of products 5- and 3-chloroanthranilic acid (**4a** & **4b**) after 1 hour with halogenase enzyme (10 μM) and substrate (0.5 mM).

Mutation of the active site tyrosines (Y443 or Y444) to either lysine or arginine had little effect on the relative activity or regioselectivity with **4**. However, the mutation E450K not only increased relative activity *ca.* 8 fold, but also enhanced *ortho* selectivity producing 93% 3-chloroanthranilate (**4b**) compared with 84% observed with the wild-type PrnA (Fig. 3 & Table S1†). On the other hand, mutation of F454 to lysine and arginine shifted the regioselectivity towards *para*, with increasing 5-chloroanthranilate (**4a**) produced (38% and 46% respectively), while also increasing relative activity by 4-fold in the case of F454K compared to the wild-type PrnA. Following further mutagenesis PrnA double mutant (E450K/F454K) was found to display a 16-fold increase in activity compared to the wild-type, producing predominantly the *para* chlorinated product (**4a**, 54%). Additionally PrnA (E450K/F454R), also exhibited increased activity, with a notable further shift towards *para* chlorination yielding 62% 5-chloroanthranilate (**4a**).

The kinetic parameters were determined for anthranilic acid (**4**) with the most interesting mutants (Table 2). The double mutant (E450K/F454K) has the lowest *K*_m_ of the mutants tested and highest *k*_cat_/*K*_m_ (55-fold higher than the wild type) which is comparable with % conversion observed in Fig. 3. PrnA E450K also shows a lower *K*_m_ of 384 μM and a *k*_cat_/*K*_m_ improvement compared to the wild type enzyme, which may be due the mutation (E450K) alleviating an unfavourable interaction between carboxylate groups of E450 and anthranilic acid. The F454K mutant on the other hand shows no improvement in *K*_m_, but exhibits a *k*_cat_ 7-fold higher than the parent enzyme. These results show that structure-guided mutagenesis can significantly increase PrnA activity as well as altering regioselectivity from predominantly *ortho*-halogenation with E450K to *para*-halogenation with PrnA (E450K/F454R).

**Fig. 4:**
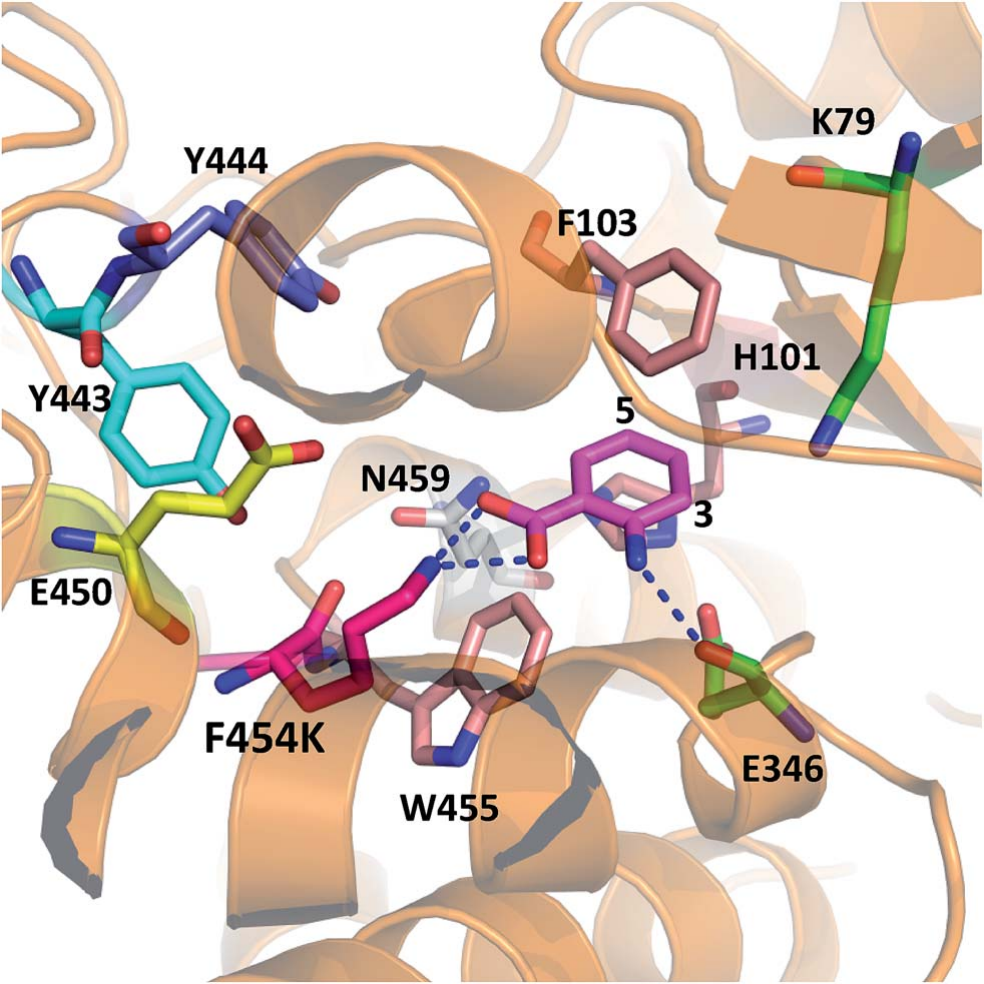
X-ray crystal structure of PrnA F454K mutant (PDB 4Z44) with anthranilic acid (**4**) positioned in the active site showing possible H-bonding interactions with the substrate.

In order to rationalise the effects of the selected mutations on the activity of PrnA, X-ray crystal structures for F454K and E450K were determined at 2.4 and 2.3 Å respectively. The F454K structure (PDB 4Z44) is similar to the wild type structure, except that an extended loop is formed instead of an α-helix from residues 435–445. Electron density for all active site residues is present and it is evident that K454 is orientated towards the substrate binding site and the catalytic residues K79 and E346 (Fig. 4). Attempts to obtain a structure of F454K with anthranilic acid bound were unsuccessful most likely due to low binding affinity of the substrate (Table 2). However, if anthranilic acid is positioned in the active site (Fig. 4) it can be seen that K454 could make a salt bridge to the carboxylate group, whilst E346 contacting the amino group of anthranilate such that electrophilic attack of the postulated K79 chloroamine would occur preferentially at C3 (also see Fig. S4†). In the E450K structure (PDB ; 4Z43) the loop region, from residues 435–445, is completely disordered and electron density from several active site residues is not defined. This may suggest that the E450K mutation has destabilized the structure preventing formation of the helical region between residues 435 and 445 observed in the wild type structure.

## Conclusions

In summary, we have expanded the range of substrates accepted by tryptophan halogenases, PrnA and PyrH, to include alternative aryl substrates. We show that structure guided mutagenesis of PrnA not only resulted in an increase of activity, but also provides an example of how regioselectivity can be improved and regiocomplementary enzymes can be created from a single parent enzyme. The concept of generating enantiocomplementary enzymes was introduced in recent years, and its application is now more widespread with increasingly significant results.^47^,^48^ The ability to manipulate enzymatic regiocontrol, in this case the orientation of aryl halogenation, could offer considerable benefit for the preparation of halogenated therapeutic and agrochemical scaffolds and other materials^1^–^3^ (also see Fig. S3†). Moreover, integration of engineered halogenases into synthetic pathways including powerful chemocatalytic cross-coupling chemistries,^4^–^10^ or biosynthetic pathways *in vivo*,^42^–^46^ could potentially be useful for production of other valuable synthetic or natural products.

## Experimental

### Cloning, expression, and mutagenesis of halogenases (PrnA and PyrH)

A synthetic gene for the halogenase from *Streptomyces rugosporus* (PyrH) was codon optimised using GeneArt® and obtained from Invitrogen (UK). The *pyrH* genes were sub-cloned into the pET28a vector containing an N-terminal His-tag using the restriction enzymes *Nde*I and *Xho*I. The PrnA encoding gene was amplified by PCR from the genomic DNA of *Pseudomonas fluorescens* BL915, with the primers *prnA* F and *prnA* R (Table S1†) using the Phusion High-Fidelity PCR master mix with GC buffer (New England Biosciences) according to the manufacturer's protocol. The PCR product was digested with *Nde*I and *Not*I and ligated into the pET28a expression vector. *E. coli* Arctic Express cells were subsequently transformed with the resulting PyrH- and PrnA-containing vectors for overexpression of the recombinant halogenases. LB medium containing kanamycin (50 μg mL^–1^) was inoculated with the transformants and incubated at 37 °C overnight. The cells were then diluted 1 : 100 in fresh LB medium and incubated shaking at 30 °C until an optical density (OD_600nm_) of 0.6. The cells were subsequently incubated at 4 °C for 30 minutes for cold-shock, and protein expression was induced with addition of IPTG (0.1 mM), before growing overnight at 15 °C, followed by harvesting of cells (4 °C, 20 min, 4000×*g*). Construction of halogenase mutants was achieved using the QuickChange® site-directed mutagenesis kit (Stratagene). The previously described constructs were used as templates and mutations were introduced *via* the manufacturer's protocol using the primers shown in Table S1.†

### Cloning and expression of the flavin reductase (Fre) and glucose dehydrogenase (GDH2)

The gene coding for the flavin reductase (Fre) from *E. coli*^49^ was amplified from *E. coli* BL21 genomic DNA using the oligonucleotides *fre* F and *fre* R (Table S1†), then digested using the restriction enzymes *Kpn*I and *Xho*I, before ligating into the pET45b expression vector containing an N-terminal His-tag. *E. coli* BL21 (DE3) cells transformed with the recombinant Fre plasmid were initially grown overnight at 37 °C in LB medium containing ampicillin (50 μg mL^–1^) before diluting 1 : 100 in fresh LB medium the following day. Cultures were subsequently incubated at 37 °C with shaking until an OD_600_ of 0.6. Recombinant protein overexpression was induced with IPTG (1 mM). Cultures were incubated for a further 5 h at 30 °C before harvesting cells (4 °C, 20 min, 4000×*g*) and purification of recombinant N-terminal His tagged Fre. A pET 21b vector containing a gene encoding GDH2 from *Bacillus megaterium*^50^ was kindly provided by Prof. Nigel Scrutton (University of Manchester). *E. coli* BL21 (DE3) cells transformed with the recombinant GDH2 plasmid were cultivated using the same method as described previously with induction of overexpression using 0.2 mM IPTG. Cultures were incubated overnight at 18 °C before harvesting cells and purification of recombinant His tagged GDH2.

### Protein purification

Cell pellets derived from the *E. coli* protein expression, were resuspended in 25 mL lysis buffer (50 mM phosphate, 500 mM NaCl, and 10 mM imidazole, pH 7.4). Lysozyme (1 mg mL^–1^) was added to the cell resuspension which was then incubated at 30 °C for 1 h. Cells were disrupted by sonication and the lysate was clarified by centrifugation (4 °C, 40 min, 10 000×*g*). The soluble cell extract was loaded onto a HisTrap™ FF crude column and purified by FPLC. The column was washed with 20 mM phosphate buffer (pH 7.4) containing 20 and 60 mM imidazole and 500 mM NaCl. Purified PyrH and PrnA were eluted in phosphate buffer containing 500 mM imidazole, Fre and GDH2 at 250 mM imidazole. Protein samples were subjected to buffer exchange with phosphate buffer containing 10% glycerol using a Vivaspin 20 centricon (10 000 MWCO) before subsequently storing at –20 °C.

### Biotransformations, and characterisation of activity and regioselectivity

The following conditions were used for assays to determine regioselectivity and %conversions (Table 1 and Fig. 3). Purified halogenase enzyme (10 μM) was incubated at 30 °C with shaking for 1 h with Fre (5 μM), FAD (1 μM), NADH (2.5 mM), MgCl_2_ (10 mM) and substrate (0.5 mM) in a total volume of 100 μL in 10 mM potassium phosphate buffer, pH 7.4. Reactions were stopped by incubating at 95 °C for 5 min and precipitated protein was removed by centrifugation before analysis *via* HPLC on an Agilent Technologies 1260 system using an Agilent Zorbax Eclipse Plus C18 4.6 × 100 mm × 3.5 μm column. For tryptophan, absorbance was measured at 280 nm. Kynurenine, anthranilamide and anthranilic acid absorbance were measured at 254 nm, with a 5 min gradient 5–75% H_2_O/acetonitrile + 0.1% formic acid.

To obtain kinetic parameters for selected reactions (Table 2) the concentration of the assay components were varied according to conditions required to provide the best fit for the Michaelis–Menten curve. However in each case the total assay volume was 150 μL and the Fre concentration was always in excess in order to ensure the production of reduced flavin was not a rate limiting factor. Assays were performed at 30 °C with shaking at 800 rpm. Plates and assay components were pre-incubated at 30 °C. Assays were started by the addition of substrate using a multi-channel pipette. Substrate was added at 15 second intervals and the reaction terminated with the addition of formic acid. All assays were performed in triplicate.

Larger scale assays were carried out to obtain chlorinated products for characterisation using halogenase (25 μM), Fre (2.5 μM), GDH2 (12.5 μM), substrate (3 mM), MgCl_2_ (100 mM), FAD (10 μM), NADH (10 μM), glucose (200 mM) in 10 mM potassium phosphate buffer, pH 7.4. Assays were run at 30 °C with shaking. Reactions were stopped by incubating at 95 °C for 5 min and precipitated protein was removed by centrifugation (4 °C, 10 min, 12 000×*g*) before analysis by HPLC. For reactions with low yield, multiple 10 mL reactions were performed and combined prior to purification by semi-prep HPLC. For tryptophan and *N*-phenylanthranilic acid, absorbance was measured at 280 nm. All other substrate and products absorbance were measured at 254 nm, with a 5 min gradient 5–75% H_2_O/acetonitrile + 0.1% formic acid. Products were subsequently characterised using 1D and 2D NMR, LRMS, HRMS and UV and are in good agreement with the literature data (see ESI†).

## Supplementary Material

Supplementary information
