# Supplementary material for: Extending the biocatalytic scope of regiocomplementary flavin-dependent halogenase enzymes
Source: Chem Sci. 2015 Apr 10;6(6):3454–60. doi: 10.1039/c5sc00913h (PMC5659210; doi:10.1039/c5sc00913h)
Supplement: Supplementary file 1 [file SC-006-C5SC00913H-s001.pdf]

Electronic Supplementary Material (ESI) for Chemical Science.
This journal is © The Royal Society of Chemistry 2015

Supporting Information

Extending the Biocatalytic Scope of Regiocomplementary Flavin-Dependent Halogenase Enzymes

Sarah A. Shepherd,[a,b] Chinnan Karthikeyan,[a,b] Jonathan Latham,[a,b] Anna-Winona Struck,[a,b] Mark L. Thompson,[a,b] Binuraj R.K. Menon,[a,b] Matthew Q. Styles,[a,b] Colin Levy,[b,c] David Leys[b,c] and Jason Micklefield[a,b,]*

[a]*School of Chemistry,* [b]*Manchester Institute of Biotechnology, and* [c]*Faculty of Life Sciences, The University of Manchester, 131 Princess Street, Manchester M1 7DN, UK.*

Content

Page S1-S6 Experimental
Page S7 – S9 Supplementary References
Page S10-S20 Supplementary Figures & Tables
Page S21-S62 HPLC, NMR, UV and MS data for halogenase reaction products.

Experimental

General All chemical reagents and solvents were purchased from Sigma-Aldrich Company Ltd, Fisher Scientific UK Ltd, or Alfa Aesar, a Johnson Matthey Company. All chemicals were used without further purification with the exception of anthranilic acid which was HPLC purified before use in assays.

5-chlorotryptophan[28] was prepared according to the general procedure described in the manuscript experimental section with tryptophan and PyrH to give 5-chlorotryptophan (87% yield). λ_{max} (H_2O/CH_3CN) 226 and 286 nm; ^1H NMR (400 MHz, D_2O) δ 7.57 (1H, d, $J_{4,6}$ = 2.0 Hz, ArH4), 7.31 (1H, d, $J_{7,6}$ = 8.7 Hz, ArH7), 7.17 (1H, s, ArH2), 7.07 (1H, dd, $J_{6,7}$ = 8.7 Hz, $J_{6,4}$ = 2.0 Hz, ArH6), 3.87 (1H, dd, $J_{9,8A}$ = 8.0 Hz, $J_{9,8B}$ = 4.9 Hz, H9), 3.28 (1H, dd, $J_{8A,8B}$ = 15.7 Hz, $J_{8A,9}$ = 4.9 Hz, $H8_A$), 3.12 (1H, dd, $J_{8B,8A}$ = 15.7 Hz, $J_{8B,9}$ = 8.0 Hz, $H8_B$); ^{13}C NMR (500/125 MHz, D_2O, HMQC, HMBC) δ 137.6 (ArC7a), 130.8 (ArC3a) 129.3 (ArC2), 127.2 (ArC5), 125.1 (ArC6), 120.4 (ArC4), 116.1 (ArC7), 110.2 (ArC3), 57.9 (C9), 29.2 (C8); LRMS-ESI (m/z) 239.0 (^{35}Cl), 241.0 (^{37}Cl) [M+H]; HRMS-ESI (m/z): calcd for $[M-NH_3]^+$ $C_{11}H_9NO_2{}^{35}Cl$ 222.0317 and $C_{11}H_9NO_2{}^{37}Cl$ 224.0287 found 222.0229 and 224.0247

7-chlorotryptophan[31] was prepared according to the general procedure described in the manuscript experimental section with tryptophan and PrnA to give 7-chlorotryptophan (84% yield). λ_{max} (H_2O/CH_3CN) 220 and 280 nm; ^1H NMR (400 MHz, D_2O) δ 7.51 (1H, d, $J_{4,5}$ = 8.0 Hz, ArH4), 7.11 (1H, s, ArH2), 7.15 (1H, d, $J_{6,5}$ = 7.6 Hz, ArH6), 7.09 (1H, t, $J_{5,4}$ = 8.0 Hz, $J_{5,6}$ = 7.6 Hz, ArH5), 3.89 (1H, dd, $J_{9,8A}$ = 7.7 Hz, $J_{9,8B}$ = 4.9 Hz, H9), 3.31 (1H, dd, $J_{8A,8B}$ = 15.3 Hz, $J_{8A,9}$ = 4.9 Hz, $H8_A$), 3.15 (1H, dd, $J_{8B,8A}$ = 15.3 Hz, $J_{8B,9}$ = 7.7 Hz, $H8_B$); ^{13}C NMR (400/101 MHz, MeOD, HMQC, HMBC) δ 170.4 (C10), 133.9 (ArC7a), 128.9 (ArC3a), 125.1 (ArC2), 120.9 (ArC6), 119.4 (ArC5), 116.5 (ArC4), 107.9 (ArC3), 53.0 (C9), 26.2(C8); LRMS-ESI m/z 239.1 (^{35}Cl), 241.1 (^{37}Cl) [M+H]; HRMS-ESI (m/z): calcd for [M+H] $C_{11}H_{12}N_2O_2{}^{35}Cl$ 239.0587 and $C_{11}H_{12}N_2O_2{}^{37}Cl$ 241.0558, found 239.0566 and 241.0535.

3-chlorokynurenine[S31] was prepared according to the general procedure described in the manuscript experimental section with kynurenine and PrnA to give 3-chlorokynurenine (84% yield). λ_{max} (H_2O/CH_3CN) 228, 264 and 368 nm; ^1H NMR (400 MHz, MeOD) δ 7.81 (1H, dd,

$J_{6,5}$ = 8.2 Hz, $J_{6,4}$ = 1.3 Hz, ArH6), 7.52 (dd, $J_{4,5}$ = 7.7 Hz, $J_{4,6}$ = 1.3 Hz, 1H, ArH4), 6.67 (1H, dd, $J_{5,6}$ = 8.2 Hz, $J_{5,4}$ = 7.7 Hz, ArH5) 4.26 (1H, dd, $J_{9,8A}$ = 7.3 Hz, $J_{9,8B}$ = 3.5 Hz, H9), 3.78 (1H, dd, $J_{8A,8B}$ = 18.6 Hz, $J_{8A,9}$ = 3.5 Hz, H8$_A$), 3.70 (1H, dd, $J_{8B,8A}$ = 18.6 Hz, $J_{8B,9}$ = 7.3 Hz, H8$_B$); ^{13}C NMR (101 MHz, MeOD) δ 200.9 (C7), 173.5 (C10), 149.0 (ArC2), 136.8 (ArC4), 132.1 (ArC6), 122.5 (ArC3), 119.5 (ArC1), 117.1 (ArC5), 51.7 (C9), 41.3 (C8); LRMS-ESI (*m/z*): 243.1 (^{35}Cl), 245.1 (^{37}Cl) [M+H]; HRMS-ESI (m/z): calcd for [M+H] $C_{10}H_{12}N_2O_3{}^{35}Cl$ 243.0536 and $C_{10}H_{12}N_2O_3{}^{37}Cl$ 245.0507, found 243.0515 and 245.0484.

5-chlorokynurenine[S31] was prepared according to the general procedure described in the manuscript experimental section with kynurenine and PyrH to give 5-chlorokynurenine (76% yield). λ_{max} (H_2O/CH_3CN) 230, 258 and 378 nm; ^{1}H NMR (500 MHz, D_2O) δ 7.85 (1H, d, $J_{6,4}$ = 2.8 Hz, ArH6), 7.38 (1H, dd, $J_{4,3}$ = 9.4 Hz, $J_{4,6}$ = 2.8 Hz, ArH4), 6.87 (1H, d, $J_{3,4}$ = 9.4 Hz, ArH3), 4.16 (1H, dd, $J_{9,8A}$ = 7.4 Hz, $J_{9,8B}$ = 5.0 Hz, H9), 3.68 (2H, m, H8$_A$+ H8$_B$); ^{13}C NMR (500MHz/125 MHz, D_2O, HSQC) δ 138.2 (ArC4), 133.6 (ArC6), 122.4 (ArC3), 53.4 (C9), 42.0 (C8); LRMS-ESI m/z 243.0, 245.0 [M+H] HRMS-ESI (m/z): calcd for [M+H] $C_{10}H_{12}N_2O_3{}^{35}Cl$ 243.0536 and $C_{10}H_{12}N_2O_3{}^{37}Cl$ 245.0507, found 243.0513 and 245.0482.

3-chloroanthranilamide[S32] was prepared according to the general procedure described in the manuscript experimental section with anthranilamide and PrnA to give 3-chloroanthranilamide (14% yield). λ_{max} (H_2O/CH_3CN) 220, 242 and 330 nm; ^{1}H NMR (400 MHz, MeOD) δ 7.52 (1H, dd, $J_{6,5}$ = 7.5 Hz, $J_{6,4}$ = 1.4 Hz, ArH6), 7.38 (1H, dd, $J_{4,5}$ = 7.9 Hz, $J_{4,6}$ = 1.4 Hz, ArH4), 6.62 (1H, dd, $J_{5,6}$ = 7.5 Hz, $J_{5,4}$ = 7.9 Hz, ArH5); LRMS-ESI (m/z) 171.0, 173.1 [M+H]; HRMS-ESI (m/z): calcd for [M+H] $C_7H_8N_2O^{35}Cl$ 171.0325 and $C_7H_8N_2O\,^{37}Cl$ 173.0296, found 171.0308 and 173.0277

5-chloroanthranilamide[S33] was prepared according to the general procedure described in the manuscript experimental section with anthranilamide and PyrH to give 5-chloroanthranilamide (19% yield). λ_{max} (H_2O/CH_3CN) 216, 254 and 340 nm; ^{1}H NMR (400 MHz, MeOD) δ 7.55 (1H, d, $J_{6,4}$ = 2.3 Hz, ArH6), 7.17 (1H, dd, $J_{4,3}$ = 8.7 Hz, $J_{4,6}$ = 2.3 Hz, ArH4), 6.74 (1H, d, $J_{3,4}$ = 8.7 Hz, ArH3). ^{13}C NMR (400MHz/101 MHz, MeOD, HSQC) δ 132.0 (ArC4), 127.8 (ArC6), 117.9 (ArC3); LRMS-ESI (m/z) 171.0, 173.0 [M+H]; HRMS-ESI (m/z): calcd for [M+H] $C_7H_8N_2O^{35}Cl$ 171.0325 and $C_7H_8N_2O\,^{37}Cl$ 173.0296, found 171.0307 and 173.0277.

3-chloroanthranilic acid[S34] was prepared according to the general procedure described in the manuscript experimental section with anthranilic acid and PrnA E450K to give 3-chloroanthranilic acid (12% yield). λ_{max} (H_2O/CH_3CN) 220, 244 and 338 nm; ^1H NMR (400 MHz, MeOD) δ 7.84 (1H, dd, $J_{6,5}$ = 8.1 Hz, $J_{6,4}$ = 1.5 Hz, ArH6), 7.42 (1H, dd, $J_{4,5}$ = 7.9 Hz, $J_{4,6}$ = 1.5 Hz, ArH4), 6.62 (1H, dd, $J_{5,6}$ = 8.1 Hz, $J_{5,4}$ = 7.9 Hz, ArH5); ^{13}C NMR (400MHz/101 MHz, MeOD, HSQC, HMBC) δ 169.8 (C7), 147.0 (ArC1), 133.3 (ArC4), 130.1 (ArC6), 119.3 (ArC3), 114.9 (ArC5), 111.5 (ArC2); LRMS-ESI m/z 172.0, 173.9 [M+H]; HRMS-ESI (m/z): calcd for [M+H] $C_7H_7NO_2{}^{35}Cl$ 172.0165 and $C_7H_7NO_2{}^{37}Cl$ 174.0136, found 172.0148 and 174.0116.

5-chloroanthranilic acid[S33] was prepared according to the general procedure described in the manuscript experimental section with anthranilic acid and PyrH to give 5-chloroanthranilic acid (9.4% yield) λ_{max} (H_2O/CH_3CN) 220, 256 and 340 nm; ^1H NMR (400 MHz, MeOD) δ 7.75 (1H, d, $J_{6,4}$ = 2.6 Hz, ArH6), 7.20 (1H, dd, $J_{4,6}$ =, 2.6 Hz, $J_{4,3}$ = 8.9 Hz, ArH4), 6.74 (1H, d, $J_{3,4}$ = 8.9 Hz, ArH3); LRMS-ESI m/z 172.0, 173.9 [M+H]; HRMS-ESI (m/z): calcd for [M+H] $C_7H_7NO_2{}^{35}Cl$ 172.0165 and $C_7H_7NO_2{}^{37}Cl$ 174.0136, found 172.0149 and 174.0117.

Chlorination of 2-amino-4-methylbenzamide was carried out according to the procedure described in the manuscript experimental section with starting material 2-amino-4-methylbenzamide and enzyme PrnA to give a mixture of 3 and 5-chlorinated products. The products were separated by HPLC to give product 2-amino-3-chloro-4-methylbenzamide (6.5% yield) and 2-amino-5-chloro-4-methylbenzamide (9.8% yield). **2-amino-3-chloro-4-methylbenzamide -** λ_{max} (H_2O/CH_3CN) 220, 250 and 328 nm; ^1H NMR (400 MHz, MeOD) δ 7.43 (1H, d, $J_{6,5}$ = 8.0 Hz, ArH6), 6.59 (1H, d, $J_{5,6}$ = 8.0 Hz, ArH5), 2.36 (3H, s, CH_3); LRMS-ESI m/z 185.0, 187.0 [M+H]; HRMS-ESI (m/z): calcd for [M+H] $C_8H_{10}N_2O^{35}Cl$ 185.0482 and $C_8H_{10}N_2O^{37}Cl$ 187.0452, found 185.0464 and 187.0433. **2-amino-5-chloro-4-methylbenzamide -** λ_{max} (H_2O/CH_3CN) 218, 256 and 338 nm; ^1H NMR (500 MHz, MeOD) δ 7.57 (1H, s, ArH6), 6.84 (1H, s, ArH3), 2.31 (3H, s, CH_3); ^{13}C NMR (125 MHz/500 MHz, MeOD, HSQC, HMBC) δ 144.5 (C2), 131.5 (C6), 122.8 (C3), 122.4 (C5), 22.2 (CH_3); LRMS-ESI m/z 185.0, 187.0 [$M+H^+$]; HRMS-ESI (m/z): calcd for [M+H] $C_8H_{10}N_2O^{35}Cl$ 185.0482 and $C_8H_{10}N_2O^{37}Cl$ 187.0452, found 185.0462 and 187.0432.

Chlorination of 2-amino-*N*-ethylbenzamide was carried out according to the procedure described in the manuscript experimental section with starting material 2-amino-*N*-ethylbenzamide and enzyme PrnA to give a mixture of 3 and 5-chlorinated products. The products were separated by HPLC to give 2-amino-3-chloro-*N*-ethylbenzamide and 2-amino-5-chloro-*N*-ethylbenzamide. Yields not recorded. **2-amino-3-chloro-*N*-ethylbenzamide -** λ_{max} (H_2O/CH_3CN) 216 and 324 nm; ^{1}H NMR (400 MHz, MeOD) δ 7.53 (1H, dd, $J_{6,5}$ = 7.8 Hz, $J_{6,4}$ = 1.4 Hz, ArH6), 7.46 (1H, dd, $J_{4,5}$ = 7.9 Hz, $J_{4,6}$ = 1.4 Hz, ArH4), 6.59 (1H, dd, $J_{5,4}$ = 7.9 Hz, $J_{5,6}$ = 7.8 Hz, ArH5), 3.39 (2H, q, $J_{9,10}$ = 7.2 Hz, C9H_2), 1.24 (3H, t, $J_{10,9}$ = 7.2 Hz, C10H_3); LRMS-ESI m/z 199.0, 201.0 [M+H$^+$]. **2-amino-5-chloro-*N*-ethylbenzamide -** λ_{max} (H_2O/CH_3CN) 218, 254 and 336 nm; ^{1}H NMR (400 MHz, MeOD) δ 7.50 (1H d, $J_{6,4}$ = 2.5 Hz, ArH6), 7.21 (1H, dd, $J_{4,3}$ = 8.7 Hz, $J_{4,6}$ = 2.5 Hz, ArH4), 6.82 (1H, d, $J_{3,4}$ = 8.7 Hz, ArH3), 3.37 (2H, q, $J_{9,10}$= 7.3 C*H*$_2$), 1.22 (3H, t, $J_{10,9}$ = 7.3 Hz, C10H_3); ^{13}C NMR (400 MHz/101 MHz, MeOD, HMQC) δ 131.1 (C4), 127.1 (C6), 118.8 (C3), 34.0 (C9) 13.4 (C10); LRMS-ESI m/z 199.0, 201.0 [M+H$^+$]; HRMS-ESI (m/z): calcd for [M+H] $C_9H_{12}N_2O^{35}Cl$ 199.0638 and $C_9H_{12}N_2O\,^{37}Cl$ 201.0609, found 199.0620 and 201.0588.

***N*-(4-chlorophenyl)anthranilic acid**[S35] was prepared according to the general procedure described in the manuscript experimental section with *N*-phenylanthranilic acid and PrnA to give *N*-(4-chlorophenyl)anthranilic acid (7% yield) λ_{max} (H_2O/CH_3CN) 222, 284 and 354 nm; ^{1}H NMR (800 MHz, MeOD) δ 7.96 (1H, d, $J_{7,8}$ = 7.5 Hz, ArH7), 7.25 (2H, d, $J_{3,2}$ = 8.8 Hz, ArH3), 7.18 (2H, d, $J_{2,3}$ = 8.8 Hz, ArH2), 7.28-7.23 (2H, m, ArH9+10), 6.76 (1H, t, $J_{8,7}$ = 7.5 Hz, ArH8); ^{13}C NMR (800 MHz/201 MHz, MeOD, HSQC, HSQC TOCSY) δ 131.8 (C7), 130.9 (C9), 128.8 (C3), 120.5 (C2), 117.4 (C8), 113.7 (C10); LRMS-ESI m/z 248.0, 250.0 [M+H]; HRMS-ESI (m/z): calcd for [M-H] $C_{13}H_9NO_2^{-35}Cl$ 246.0327 and $C_{13}H_9NO_2^{-37}Cl$ 248.0297, found 246.0363 and 248.0457

Enzyme Preparation, Crystallization, and Soaking. For crystallographic trials, E450K and F454K variant enzymes for PrnA halogenase from *Pseudomonas fluorescens* were prepared by overexpressing in Origami™ 2(DE3) Singles™ competent cells from Novagen. Bacterial cells containing the pet28b_PrnA plasmid (Hexa His tagged at C and N terminal) were grown in 2YT medium at 37°C for 2 h, before decreasing the temperature to 24°C until OD_{600} 0.6. Recombinant protein overexpression was induced with IPTG (0.1 mM) and the cells

incubated at 18°C for a further 24 h prior to harvesting by centrifugation (4°C, 20 min, 4000 x *g*,). Proteins were purified initially using an Ni^{2+} sepharose affinity tag procedure (as described previously), followed by loading onto a 140 ml Sephadex 200 size exclusion chromatography column (pre-equilibrated with 50 mM potassium phosphate, 500 mM NaCl, pH 7.2). Monomeric homogeneous variant enzyme solution was subsequently diluted (12.5 mg/ml) in crystallization buffer (20 mM HEPES, 100 mM NaCl, pH 7.2 buffer) and mixed in a 1:1 ratio with the precipitant solution (commercially available protein crystallization screen solution Morpheus®, single reagent, tube Number c3 from Molecular Dimensions). The composition of the precipitant solution was as follows: 1.0 M Imidazole and MES Buffer Mix at pH 6.5, containing 0.09 M nitrate phosphate sulfate salt mix ($NaNO_3$; Na_2HPO_4; $(NH4)_2SO_4$)) and 60% w/v glycerol and polyethylene glycol 4K). Crystals were grown by vapour diffusion at 4°C overnight.

Data Collection, Model Building, and Refinement. Data were collected at Diamond Light Source from single cryofrozen crystals of PrnA variant F454K and E450K crystals to a resolution of 2.4Å and 2.3Å respectively. (Table S3).

All data were processed and scaled using XDS and the structures solved by molecular replacement in Phaser (Search model 2AQJ). Iterative cycles of rebuilding and refinement were carried out in COOT and Phenix, validation with MOLPROBITY was integrated into the iterative rebuild process. Further validation of the final model was carried out using PDB_REDO. The crystal structures for F454K and E450K are deposited in the protein data bank (pdb files 4Z44 and 4Z43).

Supplementary References

S1 S. S. Antonysamy, B. Aubol, J. Blaney, M. F. Browner, A. M. Giannetti, S. F. Harris, N. Hébert, J. Hendle, S. Hopkins, E. Jefferson, et al., *Bioorg. Med. Chem. Lett.* 2008, **18**, 2990–5.

S2 D. Joseph-McCarthy, K. Parris, A. Huang, A. Failli, D. Quagliato, E. G. Dushin, E. Novikova, E. Severina, M. Tuckman, P. J. Petersen, et al., *J. Med. Chem.* 2005, **48**, 7960–9.

S3 N. Koay, L. Campeau, *J. Heterocycl. Chem.* 2011, **48**, 473–478.

S4 B. G. Shearer, H. S. Patel, A. N. Billin, J. M. Way, D. a Winegar, M. H. Lambert, R. X. Xu, L. M. Leesnitzer, R. V Merrihew, S. Huet, et al., *Bioorg. Med. Chem. Lett.* 2008, **18**, 5018–22.

S5 P. Filippakopoulos, S. Picaud, O. Fedorov, M. Keller, M. Wrobel, O. Morgenstern, F. Bracher, S. Knapp, *Bioorg. Med. Chem.* 2012, **20**, 1878–86.

S6 K. Ozaki, Y. Yamada, T. Oine, *J. Med. Chem.* 1985, **28**, 568–576.

S7 M. Pippel, B. D. Allison, V. K. Phuong, L. Li, M. F. Morton, C. Prendergast, X. Wu, N. P. Shankley, M. H. Rabinowitz, *Bioorg. Med. Chem. Lett.* 2009, **19**, 6373–5.

S8 M. Pippel, K. Boyce, H. Venkatesan, V. K. Phuong, W. Yan, T. D. Barrett, G. Lagaud, L. Li, M. F. Morton, C. Prendergast, et al., *Bioorg. Med. Chem. Lett.* 2009, **19**, 6376–8.

S9 U. Schindler, H. Strobel, K. Schönafinger, *Mol. Pharmacol.* 2006, **69**, 1260–1268.

S10 A. M. Doody, A. L. Antosh, W. J. Brown, *Biochem. Biophys. Res. Commun.* 2009, **388**, 695–9.

S11 A. Pajor, N. Sun, *Biochemistry* 2013, **52**, 2924–2932.

S12 G. P. Lahm, T. M. Stevenson, T. P. Selby, J. H. Freudenberger, D. Cordova, L. Flexner, C. a Bellin, C. M. Dubas, B. K. Smith, K. a Hughes, et al., *Bioorg. Med. Chem. Lett.* 2007, **17**, 6274–9.

S13 G. P. Lahm, T. P. Selby, J. H. Freudenberger, T. M. Stevenson, B. J. Myers, G. Seburyamo, B. K. Smith, L. Flexner, C. E. Clark, D. Cordova, *Bioorg. Med. Chem. Lett.* 2005, **15**, 4898–906.

S14 G. M. Karp, *J. Org. Chem.* 1995, **60**, 5814–5819.

S15 C. Gnamm, A. Jeanguenat, A. C. Dutton, C. Grimm, D. P. Kloer, A. J. Crossthwaite, *Bioorg. Med. Chem. Lett.* 2012, **22**, 3800–6.

S16 K. Kimura, T. Kitagawa, O. Uchida, *Method of Producing Benzoxazinone-Based Compound*, 2009, Patent WO 2009/041715 A1.

S17 N. Griebenow, A. Buchmüller, P. Kolkhof, H. Bischoff, *Substituted Benzoxepino-Isoxazoles and Use Thereof*, 2007, WO 2008/003424 A1.

S18 B. Aquila, L. Dakin, J. Ezhuthachan, J. Lee, P. Lyne, T. Pontz, *Substituted Quinazolones as Anti-Cancer Agents*, 2008, US 2008/0275022 A1.

S19 A. Krasinski, S. Punna, S. Ungashe, Q. Wang, Y. Zeng, *Fused Heteroaryl Pyridyl and Phenyl Benzenesuflonamides as CCR2 Modulators for the Treatment of Inflammation*, 2011, US 2011/0319433 A1.

S20 N. Ip, F. Ip, Y. Hu, Y. Han, S. Chung, *Heterodimers And Methods Of Using Them*, 2009, US 7,605,265 B2.

S21 A. Nardi, M. Grunnet, J. Demnitz, T. D. Jensen, P. Christophersen, D. S. Jones, *Novel Cinnamic Amide Derivatives Useful as Ion Channel Modulators*, 2007, WO 2008/074755 A2.

S22 P. Chen, A. Daugan, R. L. M. Gosmini, D. Igo, L. Katrincic, P. Martres, E. Nicodeme, *Compounds Which Increase Apolipoprotein A-1 Production and Uses Thereof in Medicine*, 2006**,** WO 2006032470 A1.

S23 M. Hadd, M. Holladay, M. Rowbottom, *Quinazoline Compounds and Methods of Use Thereof*, 2012, WO 2012/030948 A1.

S24 T. W. Butler, A. F. J. Fliri, R. J. Gallaschun, B. P. Jones, J. A. Ragan, *Tetrahydroquinazoline-2, 4-Diones and Therapeutic Uses Thereof*, 2001, EP 1 083 178 A1.

S25 P. D. Adams, P. V. Afonine, G. Bunkoczi, V. B. Chen, I. W. Davis, N. Echols, J. J. Headd, L. W. Hung, G. J. Kapral, R. W. Grosse-Kunstleve, A. J. McCoy, N. W. Moriarty, R. Oeffner, R. J. Read, D. C. Richardson, J. S. Richardson, T. C. Terwilliger, P. H. Zwart, *Acta Crystallographica Section D-Biological Crystallography* 2010, **66**, 213-221.

S26 V. B. Chen, W. B. Arendall, J. J. Headd, D. A. Keedy, R. M. Immormino, G. J. Kapral, L. W. Murray, J. S. Richardson, D. C. Richardson, *Acta Crystallographica Section D-Biological Crystallography,* 2010, **66**, 12-21.

S27 P. Emsley, K. Cowtan, *Acta Crystallographica Section D-Biological Crystallography,* 2004, **60**, 2126-2132.

S28 R. P. Joosten, F. Long, G. N. Murshudov, A. Perrakis, *IUCrJ,* 2014, **1**, 213-220.

S29 W. Kabsch, *Acta Crystallographica Section D-Biological Crystallography* 2010, **66**, 125-132.

S30 A. J. McCoy, R. W. Grosse-Kunstleve, P. D. Adams, M. D. Winn, L. C. Storoni, R. J. Read, *J Appl Crystallogr*, 2007, **40**, 658-674.

S31 C. Maitrani, R. S. Phillips, *Bioorg. Med. Chem.* 2013, **21**, 4670–7.

S32 A. Nathubhai, R. Patterson, T. J. Woodman, H. E. C. Sharp, M. T. Y. Chui, H. H. K. Chung, S. W. S. Lau, J. Zheng, M. D. Lloyd, A. S. Thompson, et al., *Org. Biomol. Chem.* 2011, **9**, 6089–99.

S33 H. Zhao, H. Fu, R. Qiao, *J. Org. Chem.* 2010, **75**, 3311–6.

S34 S. Tani, N. Yadomatsu, A. Ikumi, Y. Hirano, T. Ido, *Nitrobenzene Compound Manufacturing Method*, 2014, EP 2 754 651 A1.

S35 A. O. Adeniji, B. M. Twenter, M. C. Byrns, Y. Jin, M. Chen, J. D. Winkler, T. M. Penning, *J. Med. Chem.* 2012, **55**, 2311–2323.

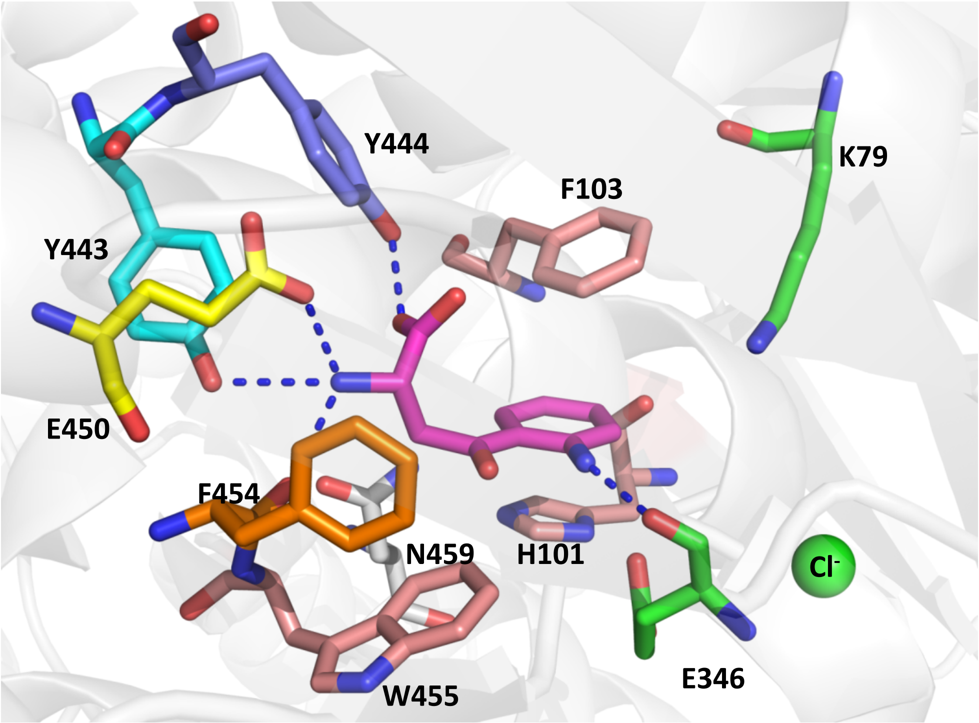

Figure S1 – Kynurenine (magenta) docked into PrnA crystal structure (2AQJ) showing possible active site interactions. Key active site residues K79 (green), H101 (pink), F103 (pink), E346 (green), Y443 (cyan), Y444 (blue), E450 (yellow), F454 (orange), W455 (pink) and N459 (grey).

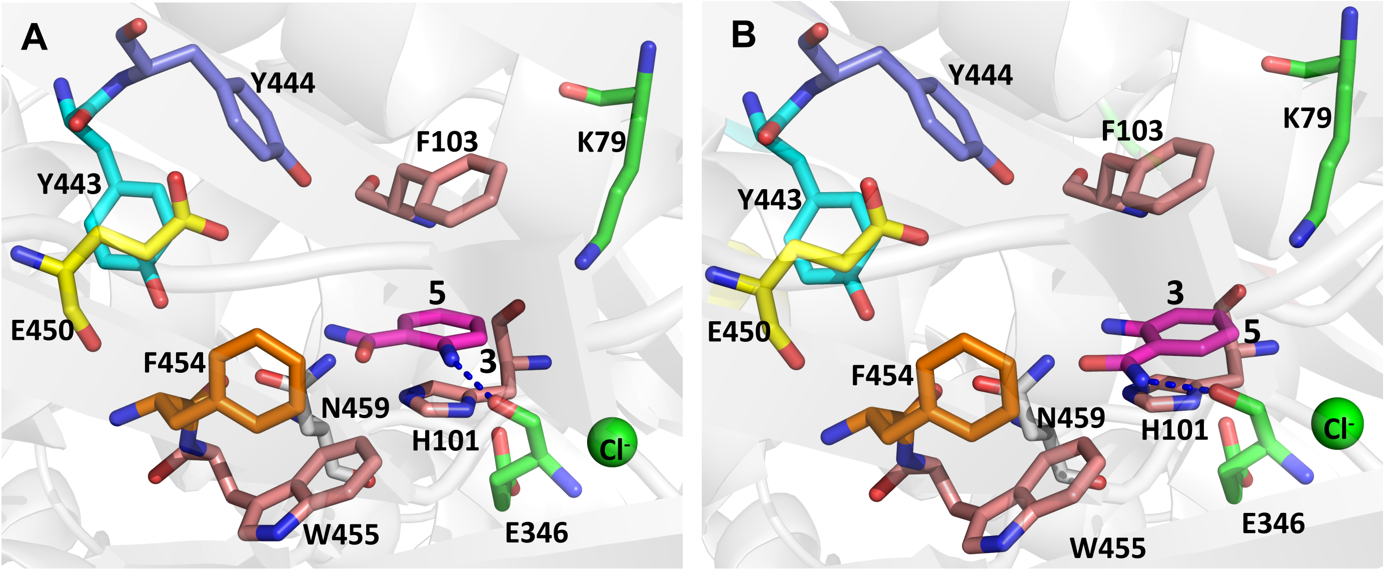

Figure S2 - Anthranilamide (magenta) docked into PrnA crystal structure (2AQJ) showing possible active site interactions. (**A**) Orientation of substrate favouring halogenation at the 3-position of anthranilamide, (**B**) Orientation of substrate favouring halogenation at the 5-position of anthranilamide. Key active site residues K79 (green), H101 (pink), F103 (pink), E346 (green), Y443 (cyan), Y444 (blue), E450 (yellow), F454 (orange), W455 (pink), N459 (grey).

A

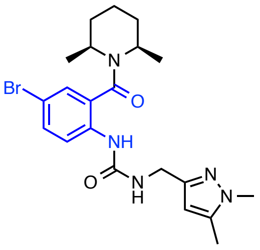

Hepatitis C virus NS5b RNA polymerase inhibitors
SGX Pharmaceuticals & Roche
Antonysamy *et al.* 2008 [S3]

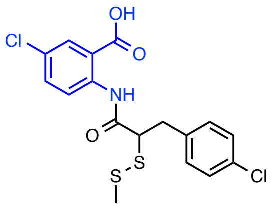

Acyl Carrier Protein Synthase Inhibitors (antibacterial)
Wyeth & Millennium Pharmaceuticals
Joseph-McCarthy *et al.* 2005 [S4]

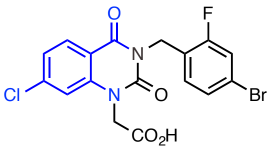

Zenarestat, an aldose reductase inhibitor
for treatment of diabetic disorders
Merck Frosst
Koay et al. 2011 [S5]

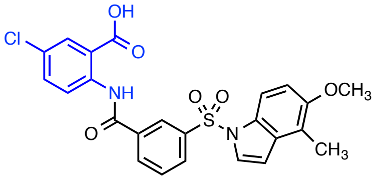

GSK1115, PPARδ partial agonist
Tool compound diabetes
GlaxoSmithKline
Shearer *et al.* 2008 [S6]

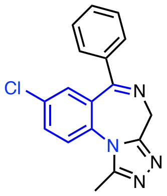

Alprazolam (Xanax), Pfizer
Benzodiazepine psychoactive drug
& also protein interaction inhibitors
Filippakopoulos *et al.* 2012 [S7]

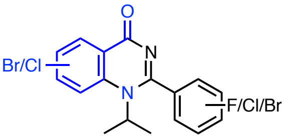

4(1*H*)-quinazolinones
Antiinflamatory agents
Tanabe Seiyaku Co.
Ozaki *et al.* 1985 [S8]

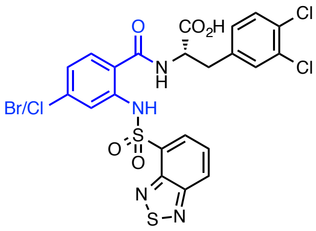

CCK1R antagonists
for Gastroesophageal reflux disease
Johnson & Johnson,
Pippel *et al.* 2009 [S9,10]

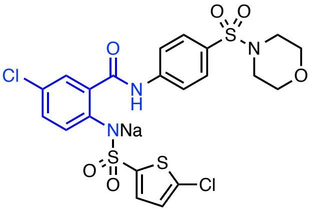

soluble guanylyl cyclase agonists
for cardiovascular disease
Sanofi-Aventis
Schindler *et al.* 2006 [S11]

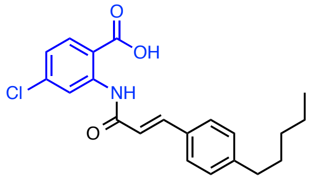

Phospholipase A_2 antagonist,
Doody *et al.* 2009 [S12]
Na^+/Dicarboxylate Symporter Inhibitor
Pajor*et al.* 2013 [S13]

B

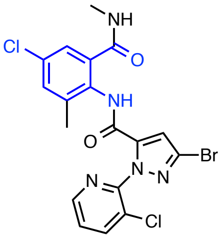

Rynaxypyr^{TM}, Insecticide
DuPont Crop Protection
Lahm *et al.* 2007 [S14,15]

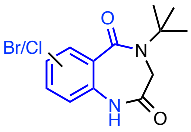

1,4-benzodiazepine-2,5-diones
Herbicides
American Cyanamid
Karp *et al.* 1995 [S16]

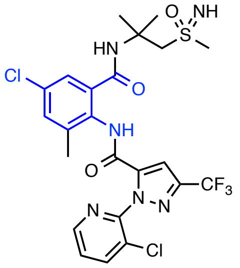

Sulfoximine Insecticides
Syngenta
Gnamm *et al.* 2012 [S17]

C

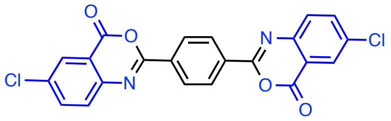

benzoxazinone-based UV absorbing materials
for optical film applications
FujiFilm, Kimura *et al* 2009 [S18]

Figure S3. Halogenated anthranilic acids derivatives have been reported widely in the scientific and patent literature and are of considerable industrial importance. The deversity of important biological targets [S1-S15] illustrated above would suggest that halo anthranilic acid represent a privileged bioactive structure: (**A**) Some examples of pharmaceuticals in use and in development that are halo anthranilic acid derivatives [S1-S11]; (**B**) Agrochemicals derived from from halo anthranilic acids [S12-S15] including DuPont's "blockbuster" insecticide Rynaxpyr (chlorantraniliprole); (C) Chloro anthranilic acid based materials (FujiFilm) [S16]; Other examples where halo anthranilic acid derivatives have been used industry are also cited [S17-S24].

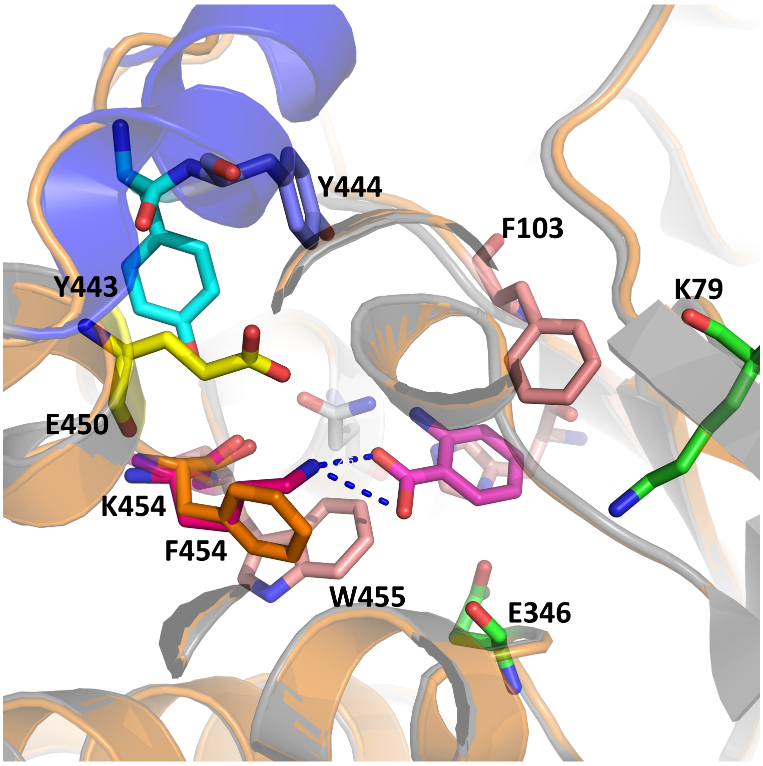

Figure S4. X-ray crystal structures of PrnA wild-type (PDB 2AQJ) in grey cartoon aligned with PrnA F454K (PDB 4Z44) in orange cartoon, with anthranilic acid **4** docked in an alternative binding mode (shown in magenta) The region in blue is a helical region in the wild type compared to a loop in the F454K structure. Only key active site residues are displayed, with mutant residue K454 shown as red sticks. The additional contact between the carboxylate group of **4** and K454, may enable an alternative substrate binding mode opposite to the one shown in Figure 4; in this case the amino group of **4** is oriented away from E346 at the opposite side of the substrate binding pocket, which would favour C5-halogenation. This would account for the increase in C5-halogenation of F454K, compared with the wild-type enzyme. In the wild-type enzyme an H-bond between the amino group of **4** and E346 is likely to be the major contact with the substrate which would lead to predominately C3 halogenation.

PrnA Mutant	% 4a	% 4b	% Conversion (4a, 4b)
Wild type	16	84	1.7
E450K	7	93	14.2
E450R	18	82	4.9
F454K	38	62	7.5
F454R	46	54	3.0
E450K F454K	54	46	27.6
E450K F454R	62	38	16.7
E450R F454K	44	56	16.2

Table S1 - Results from PrnA mutants with Anthranilic acid, showing % conversion of 5-chloroanthranilic acid (4a) and 3-chloroanthranilic (4b) as well as total % conversion

prnA F	5'-AAAAAACATATGATGAACAAGCCAATCAAGAA-3'
prnA R	3'-AAAAAAGCGGCCGCCTACTGGCGTTCCTGAGCC-5'.
fre F	5'-AAAAAAGGTACCATGACAACCTTAAGCTGTAAA-3'
fre R	3'-AAAAAACTCGAGTCAGATAAATGCAAACGCATC-5'
prnA E450K F	5'-CCACGTATCACGAGACCTTCGACTACAAATTCAAG-3'
prnA E450K R	5'-GCCGTTCAACCAGAAGTTCTTGAATTTGTAGTC-3'
prnA F454K	5'-CGACTACGAATTCAAGAATAAATGGTTGAACGGCAACTACT-3'
prnA F454R	5'-CGACTACGAATTCAAGAATCGCTGGTTGAACGGCAACTACT-3'
prnA Y444K	5'-CGTTCGACGATTCCACGTACAAAGAGACCTTCGACTACGAATT-3'
prnA Y444R	5'-CGTTCGACGATTCCACGTACCGCGAGACCTTCGACTACGAATT-3'
prnA Y443K	5'-GTCGTTCGACGATTCCACGAAATACGAGACCTTCGACTACG-3'
prnA Y443R	5'-GTCGTTCGACGATTCCACGCGCTACGAGACCTTCGACTACG-3'
prnA E450K F454K	5'-CTACGAGACCTTCGACTACAAATTCAAGAATAAATGGTTG-3'
prnA E450K F454R	5'-CTACGAGACCTTCGACTACAAATTCAAGAATCGCTGGTTG-3'
prnA E450R F454K	5'-GTACTACGAGACCTTCGACTACCGTTTCAAGAATAAATGGTTGAACG-3'

Table S2 – Primers used in this study

Data collection	F454K (4Z44)<br>FAD and Cl	E450K (4Z43)<br>FAD and Cl
Wavelength (Å)	0.977	0.977
Resolution range (Å)	68.84-2.20 (2.28-2.20)	30.85-2.29 (2.37-2.29)
Space group	P 43 21 2	P 43 21 2
Unit cell	68.1 68.1 275.38 90 90 90	68.74 68.74 276.17 90 90 90
Unique reflections	33920 (3298)	30949 (3030)
Completeness (%)	99.96 (99.82)	99.92 (99.93)
Mean I/sigma(I)	8.31 (3.80)	22.16 (3.44)
Wilson B-factor	31.37	47.95
R-work	0.1795	0.2075
R-free	0.2325	0.2578
Number of atoms	4561	4220
macromolecules	4158	4067
ligands	59	53
water	344	100
RMS(bonds)	0.008	0.009
RMS(angles)	1.13	1.21
Ramachandran favored (%)	97	94.2
Ramachandran outliers (%)	0.19	0.40
Clashscore	2.41	7.71
Average B-factor	26.00	51.2
macromolecules	25.70	51.3
ligands	24.60	46.5
solvent	30.10	48.9

Table S3 - Crystallographic data and refinement statistics [S25-29]

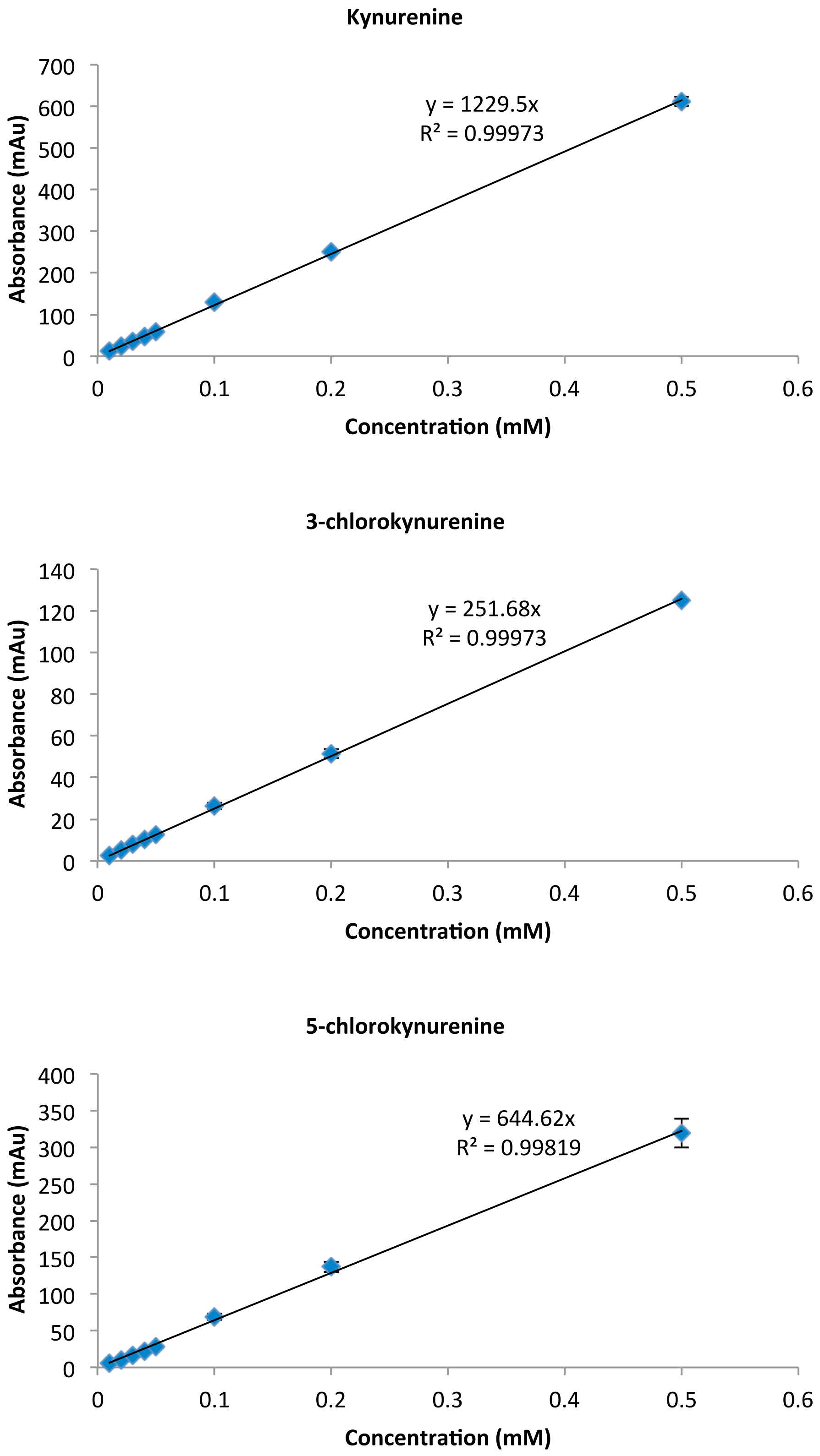

Figure S5 Kynurenine HPLC calibrations

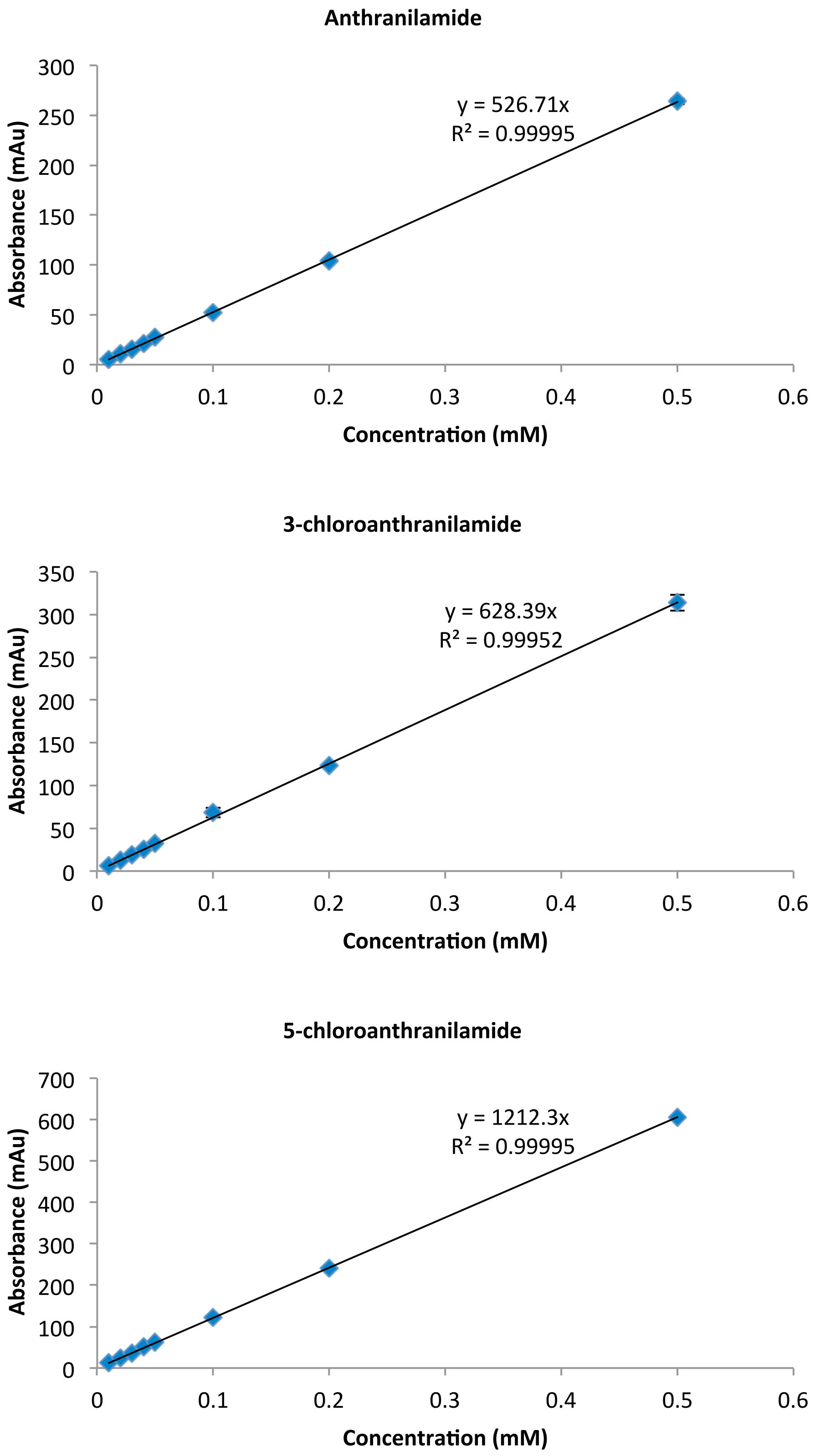

Figure S6 Anthranilamide HPLC calibrations

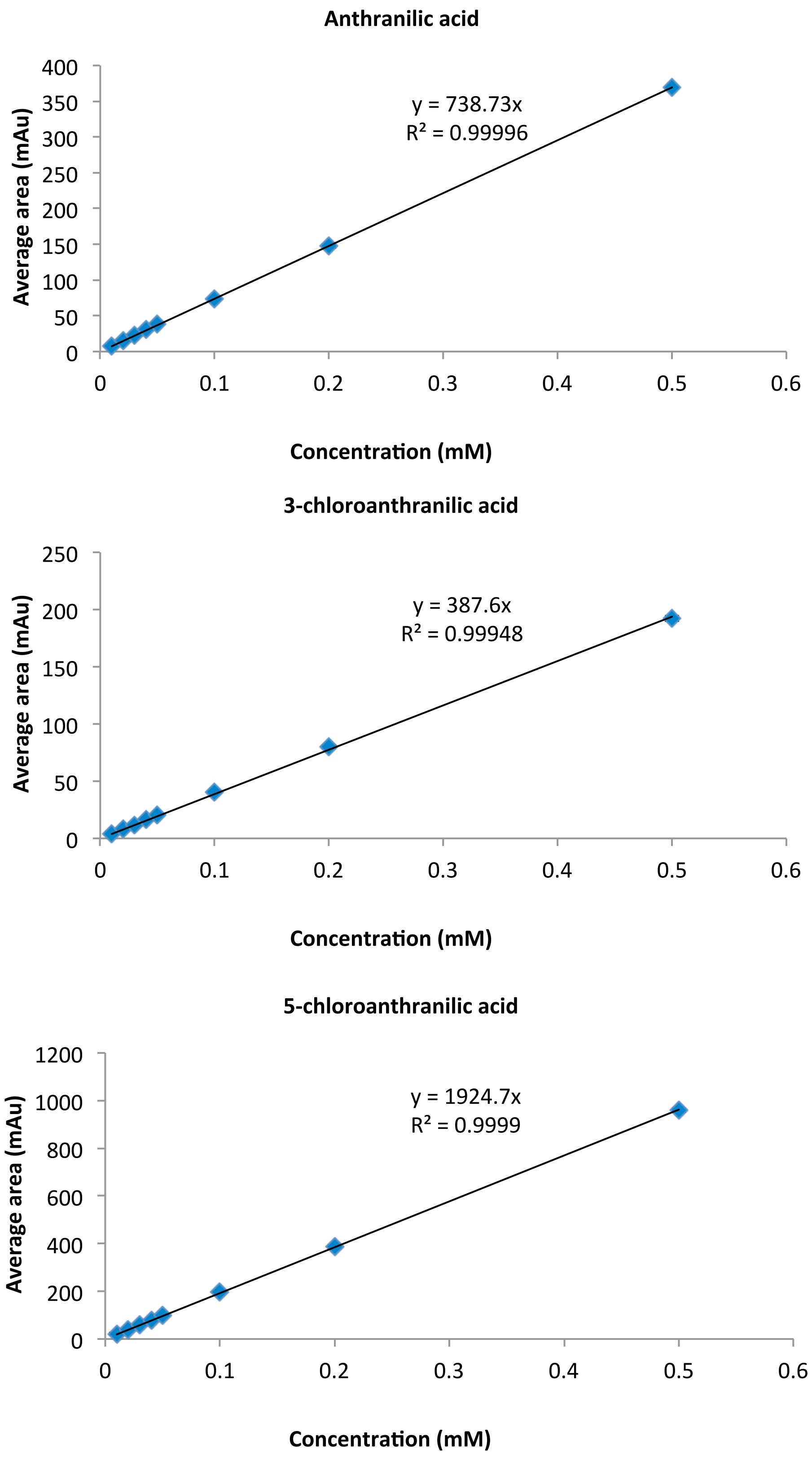

Figure S7 Anthranilic acid HPLC calibrations

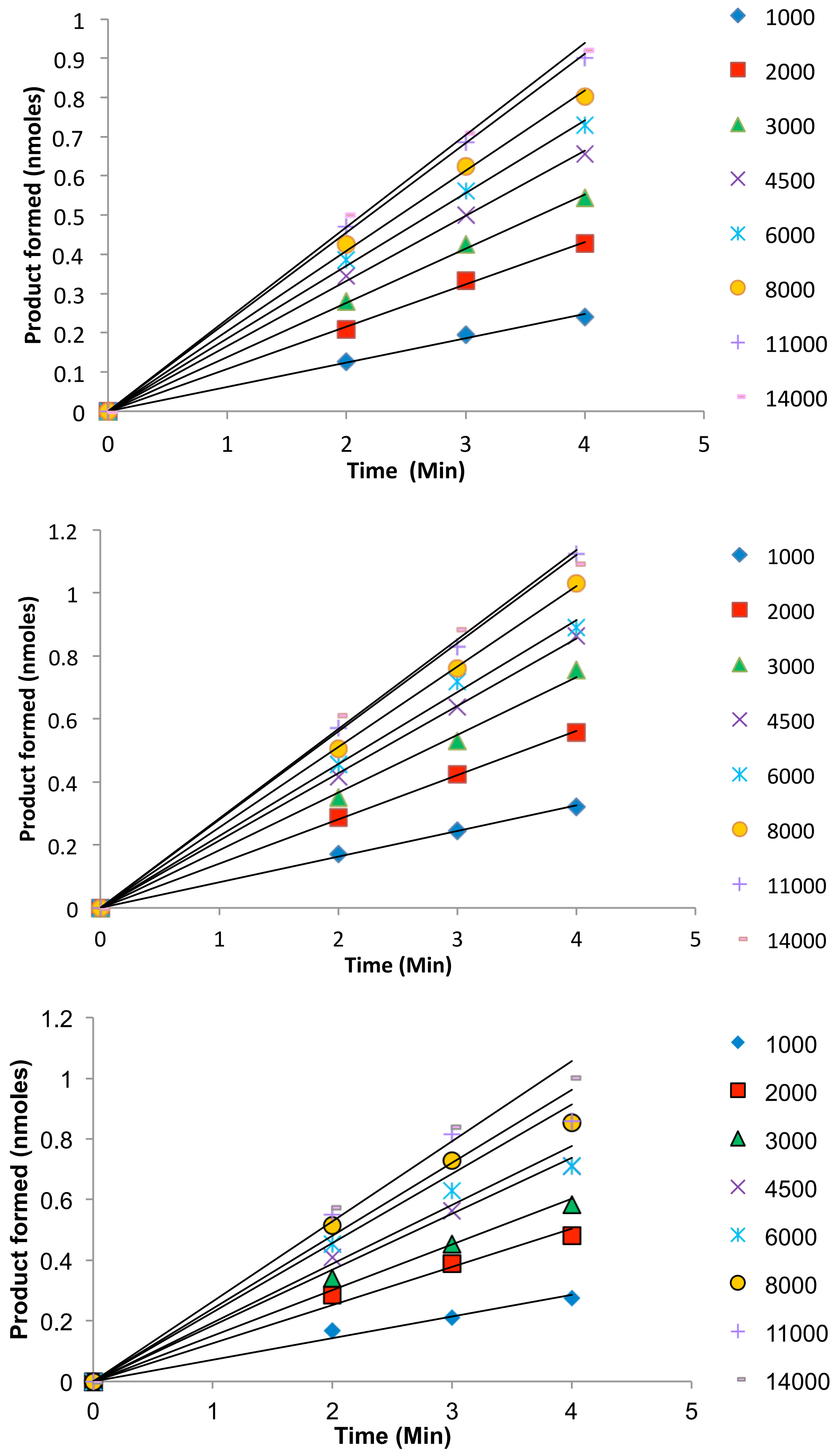

Figure S8 Representative kinetic data - Rate graphs - PrnA wild-type with anthranilamide (**3**)

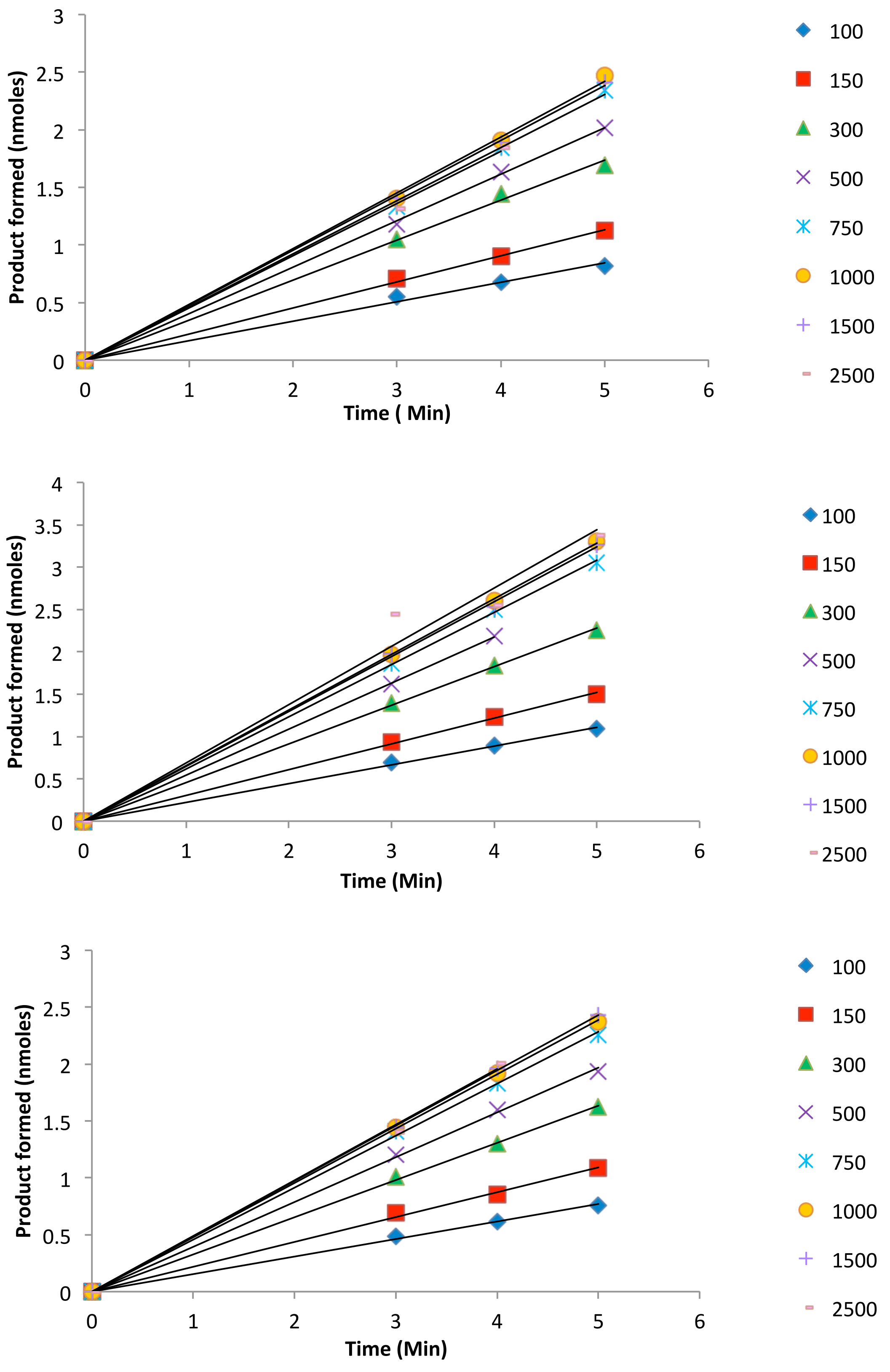

Figure S9 Representative kinetic data - Rate graphs - PrnA E450K F454K with anthranilic acid (**4**)

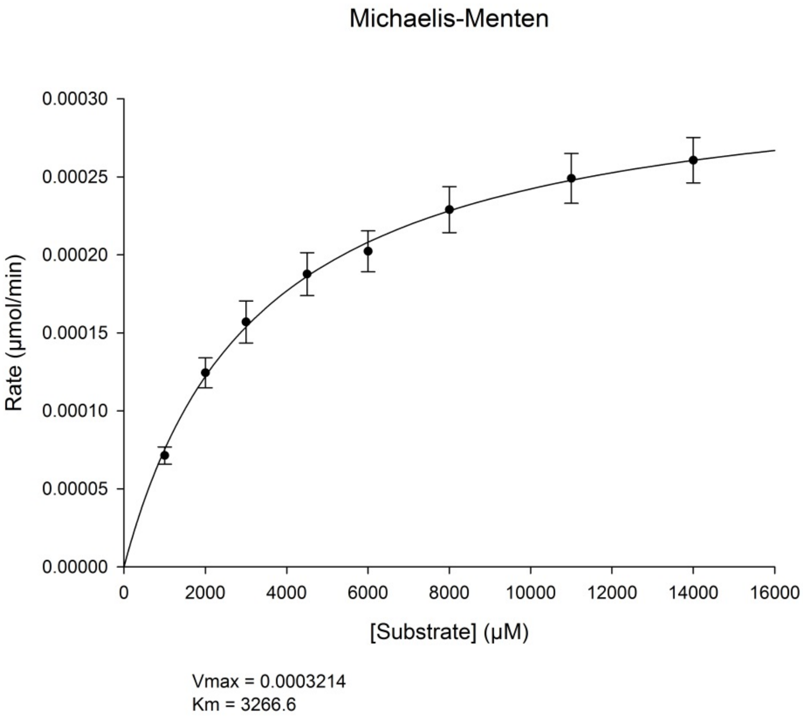

Figure S10 - Michaelis-Menten graph of PrnA wild-type with anthranilamide (**3**)

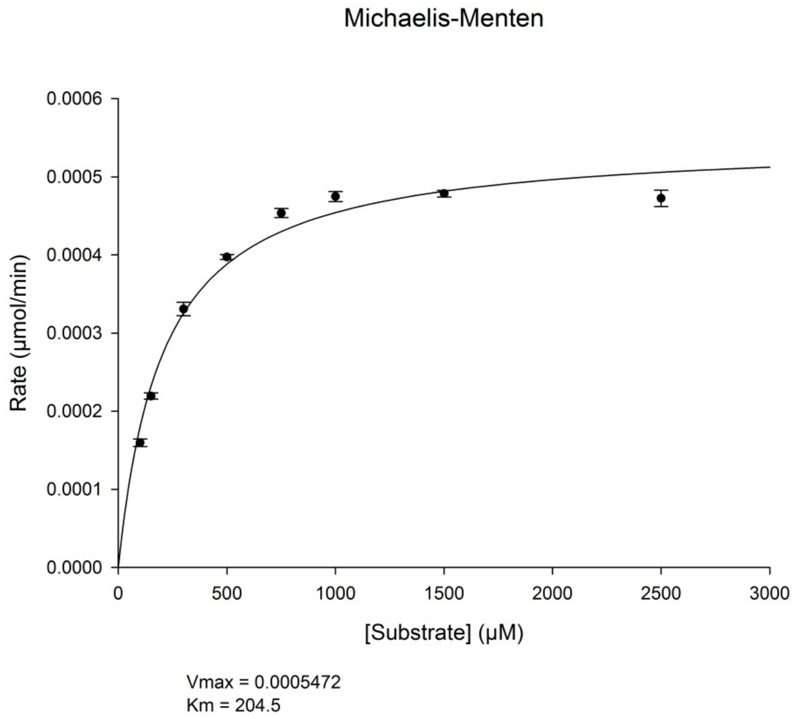

Figure S11 - Michaelis-Menten graph of PrnA E450K F454K with anthranilic acid (**4**)

Original data

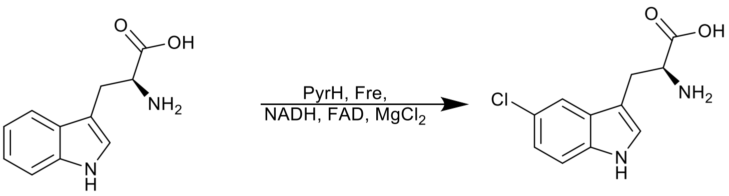

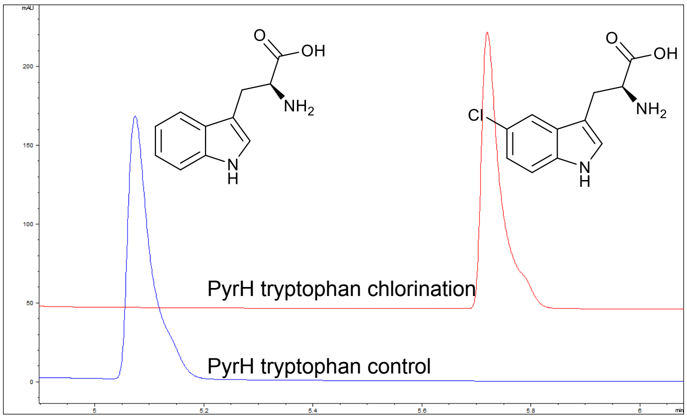

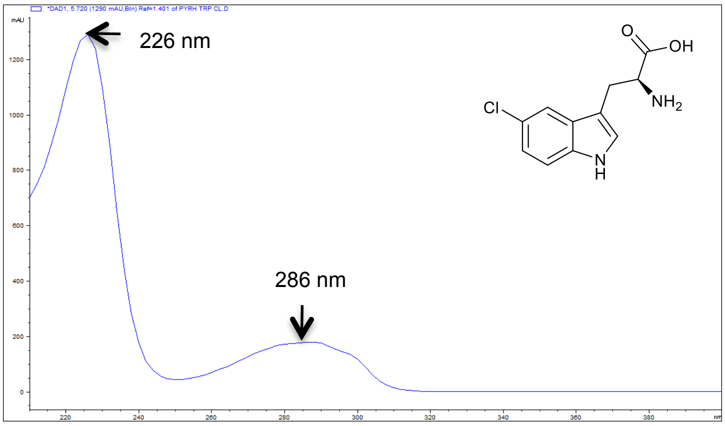

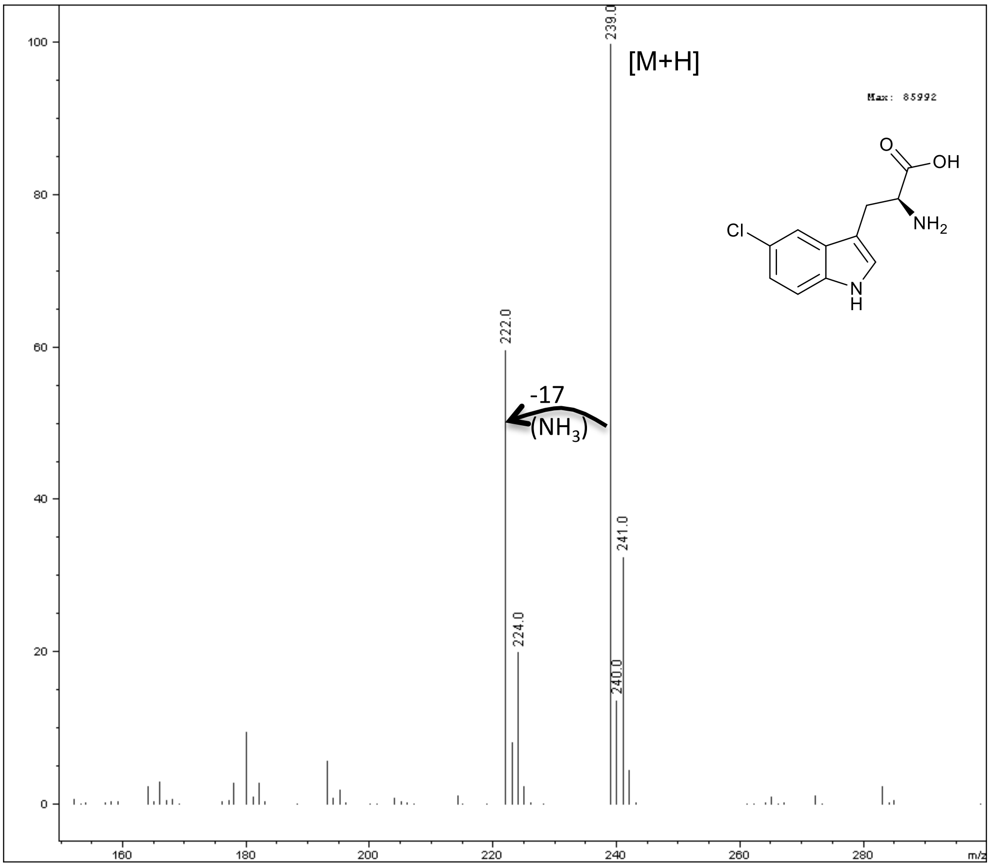

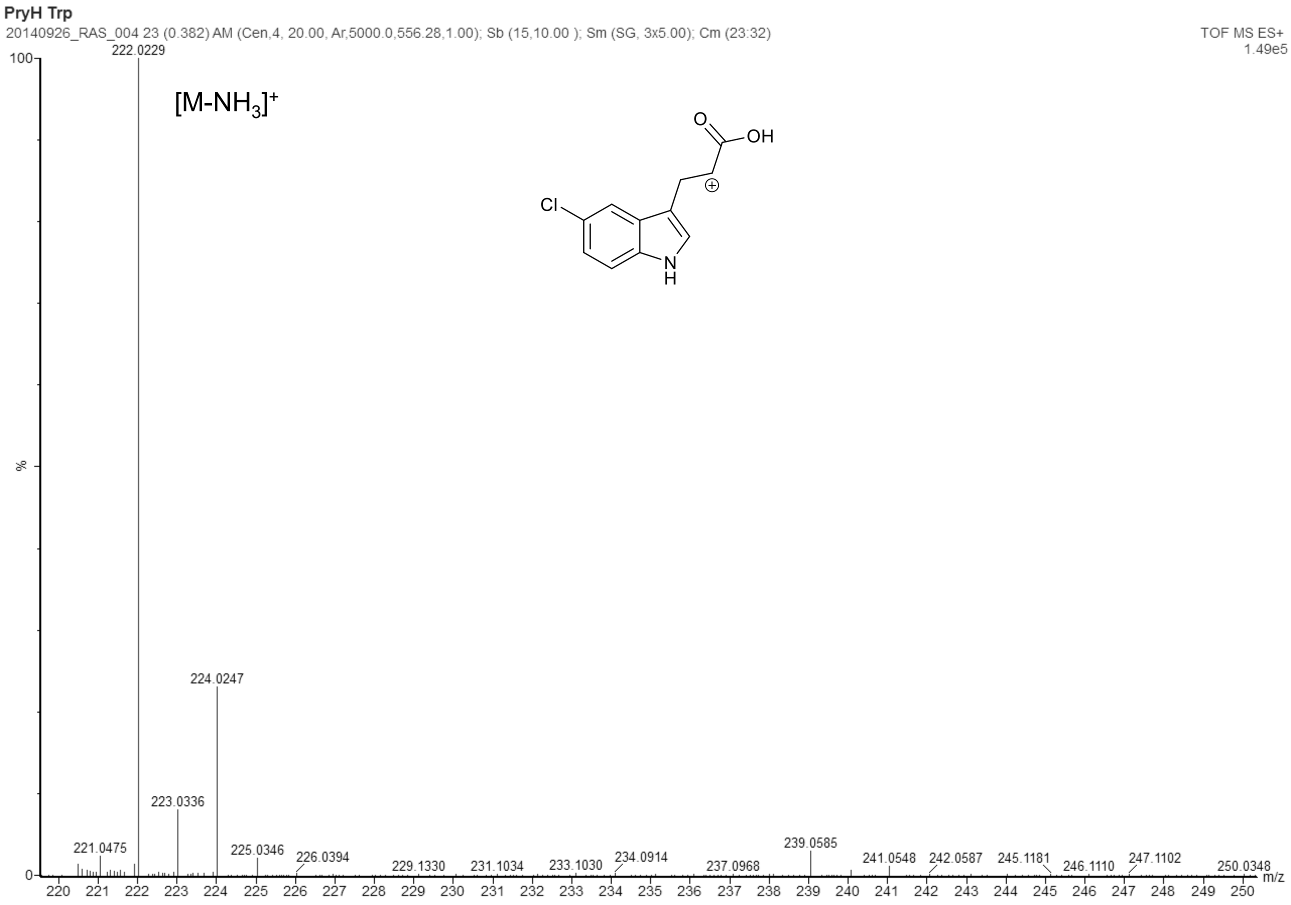
PryH Trp
20140926_RAS_004 23 (0.382) AM (Cen,4, 20.00, Ar,5000.0,556.28,1.00); Sb (15,10.00); Sm (SG, 3x5.00); Cm (23:32)
TOF MS ES+
1.49e5
[M-NH3]+
222.0229
224.0247
223.0336
221.0475
225.0346
226.0394
229.1330
231.1034
233.1030
234.0914
237.0968
239.0585
241.0548
242.0587
245.1181
246.1110
247.1102
250.0348
m/z
%

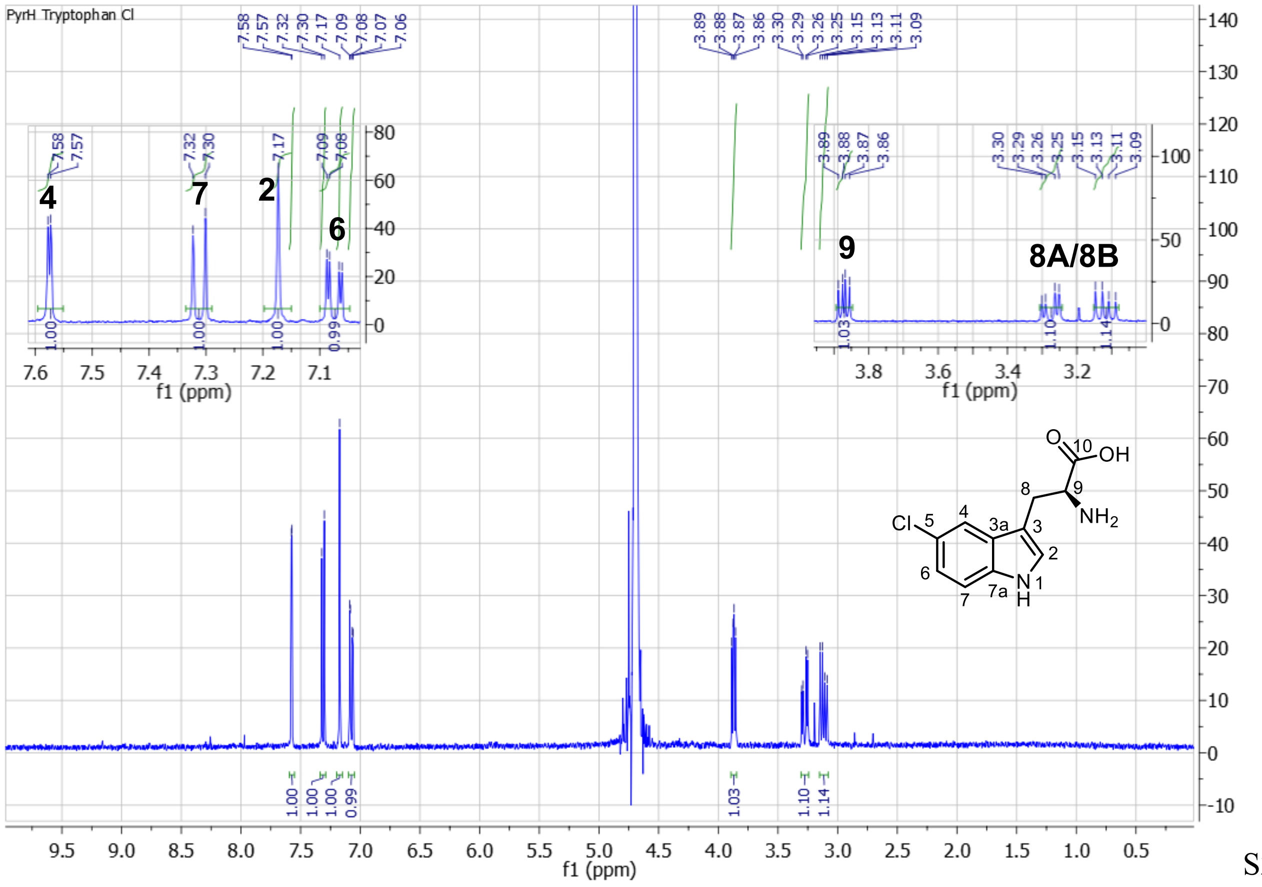
PryH Tryptophan Cl
4
7
2
6
9
8A/8B
f1 (ppm)

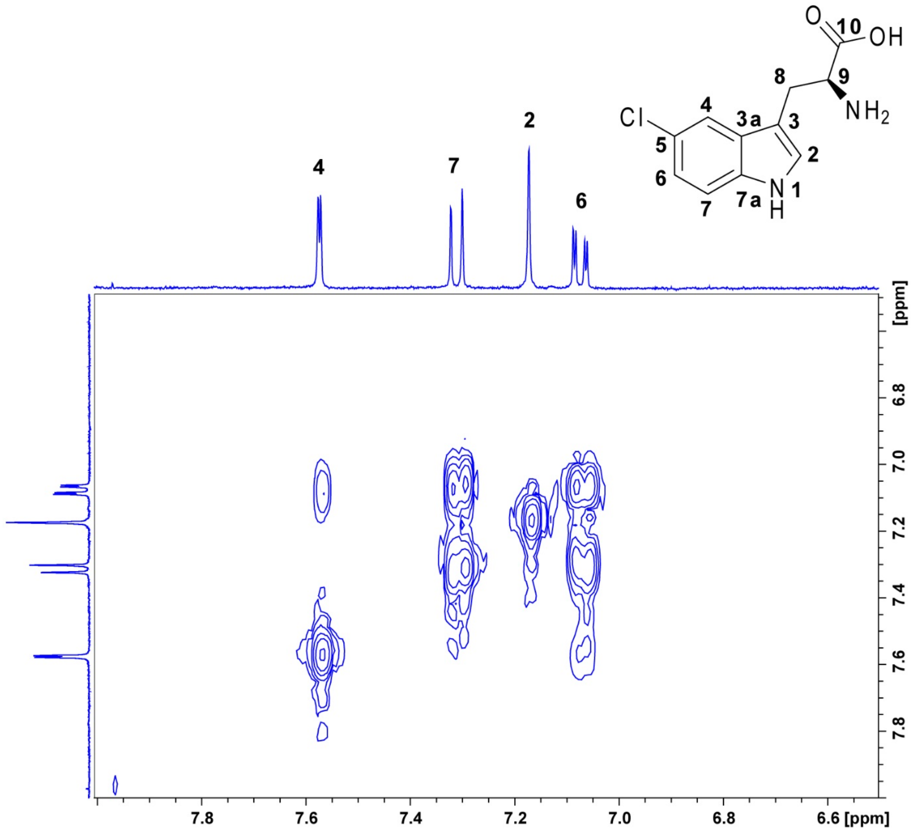

COSY of 5-chlorotryptophan

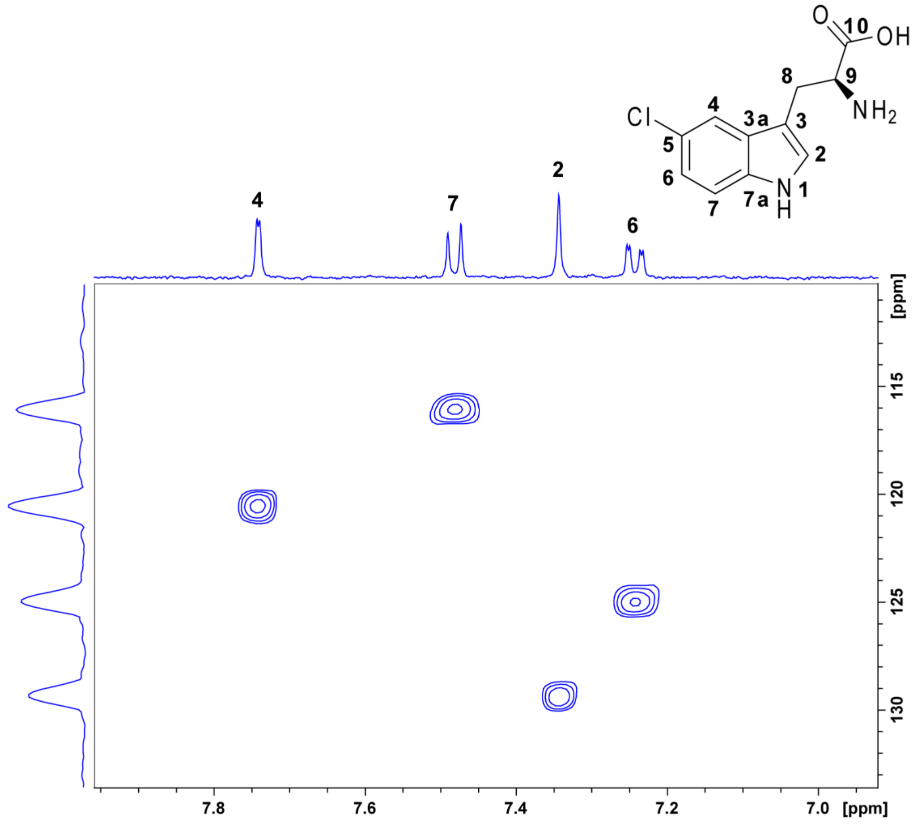

HSQC of 5-chlorotryptophan

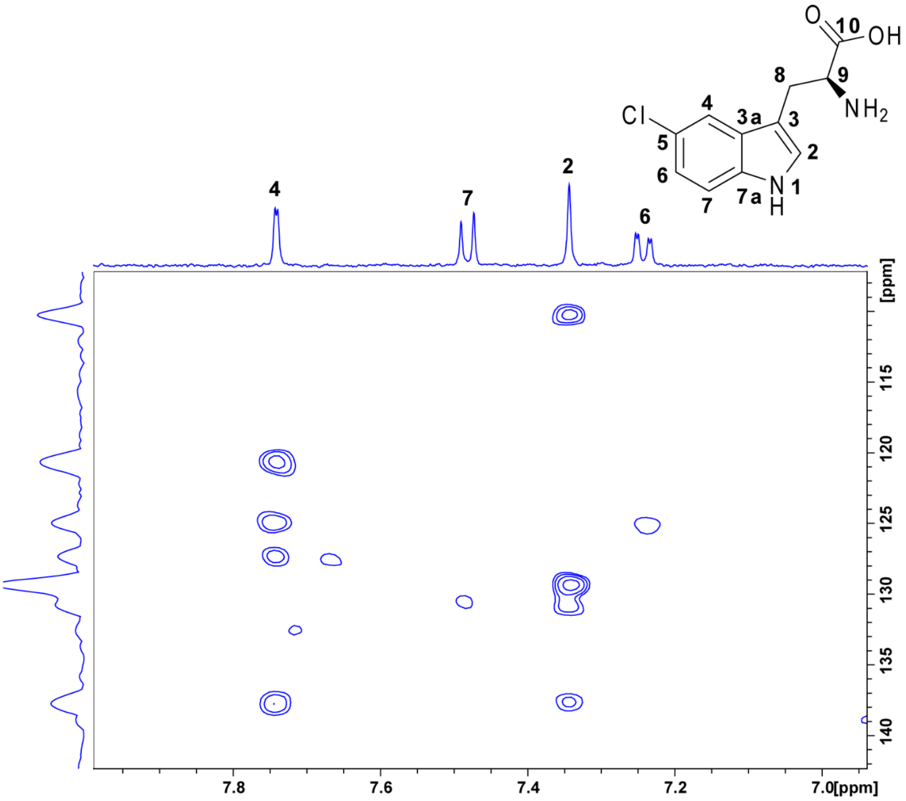

HMBC of 5-chlorotrytophan

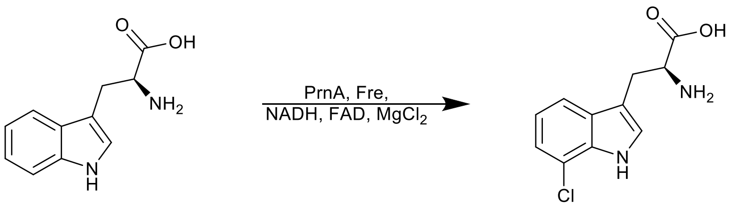
PrnA, Fre,
NADH, FAD, MgCl2

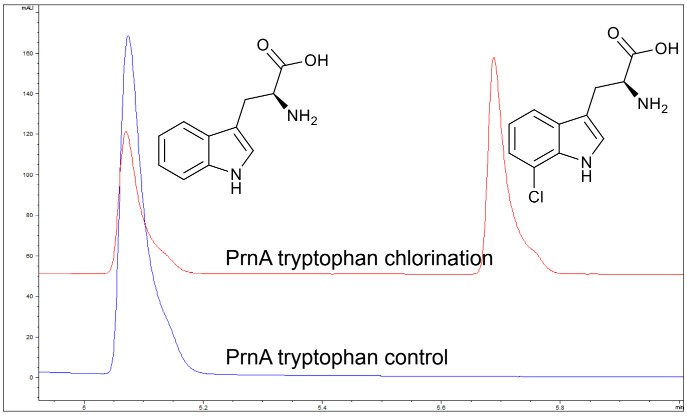
PrnA tryptophan chlorination
PrnA tryptophan control

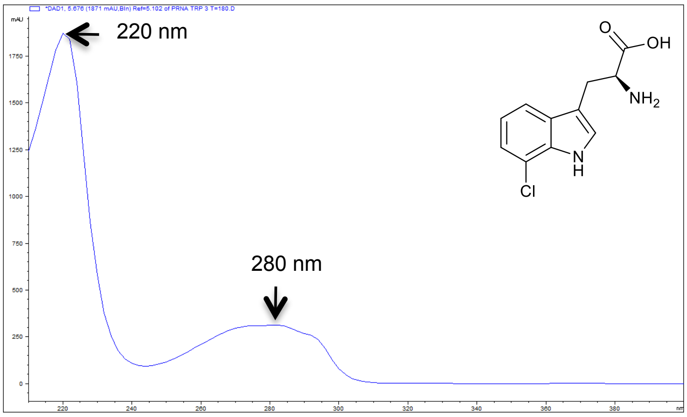
220 nm
280 nm

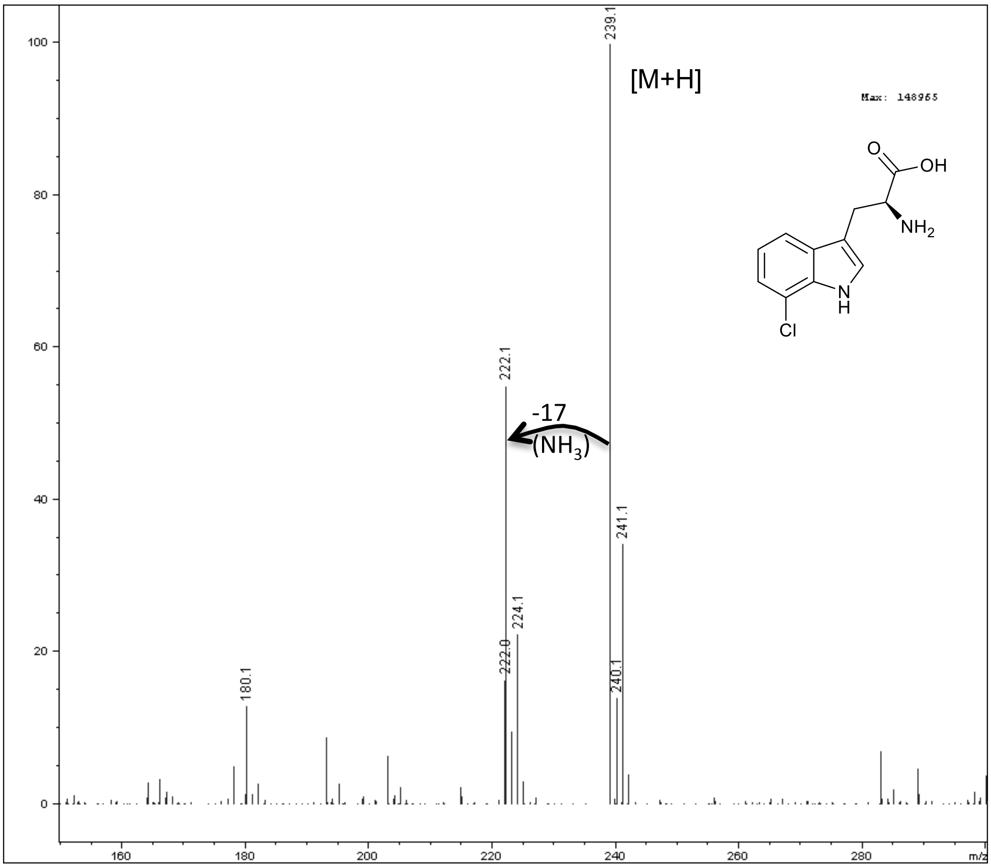
[M+H]
239.1
222.1
-17
(NH3)
241.1
224.1
222.0
240.1
180.1

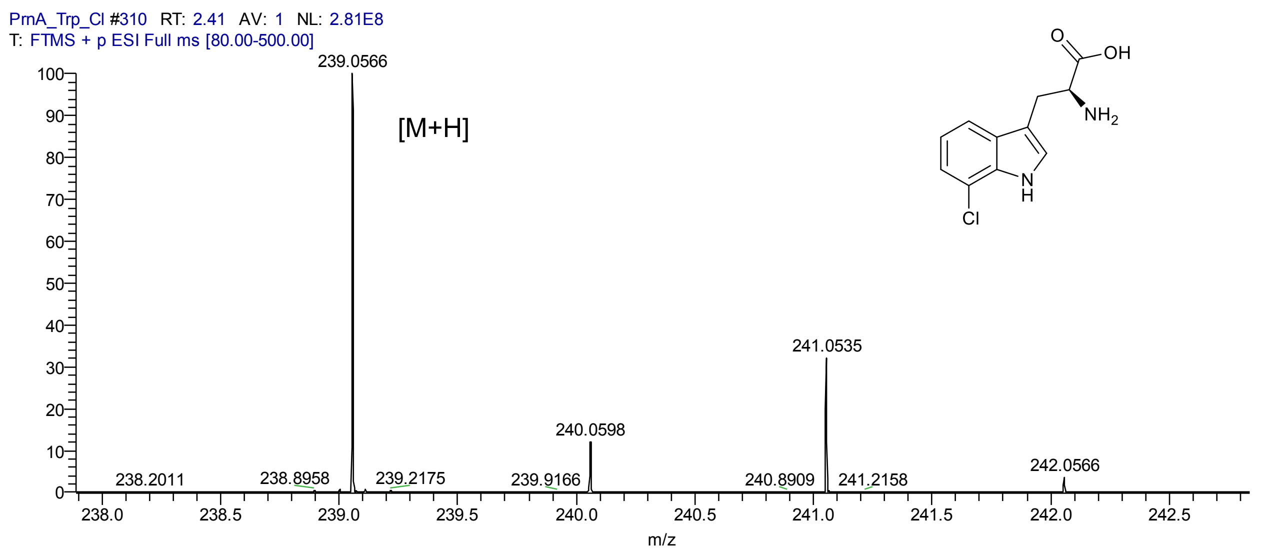
PmA_Trp_Cl #310 RT: 2.41 AV: 1 NL: 2.81E8
T: FTMS + p ESI Full ms [80.00-500.00]
239.0566
[M+H]
241.0535
240.0598
242.0566
m/z

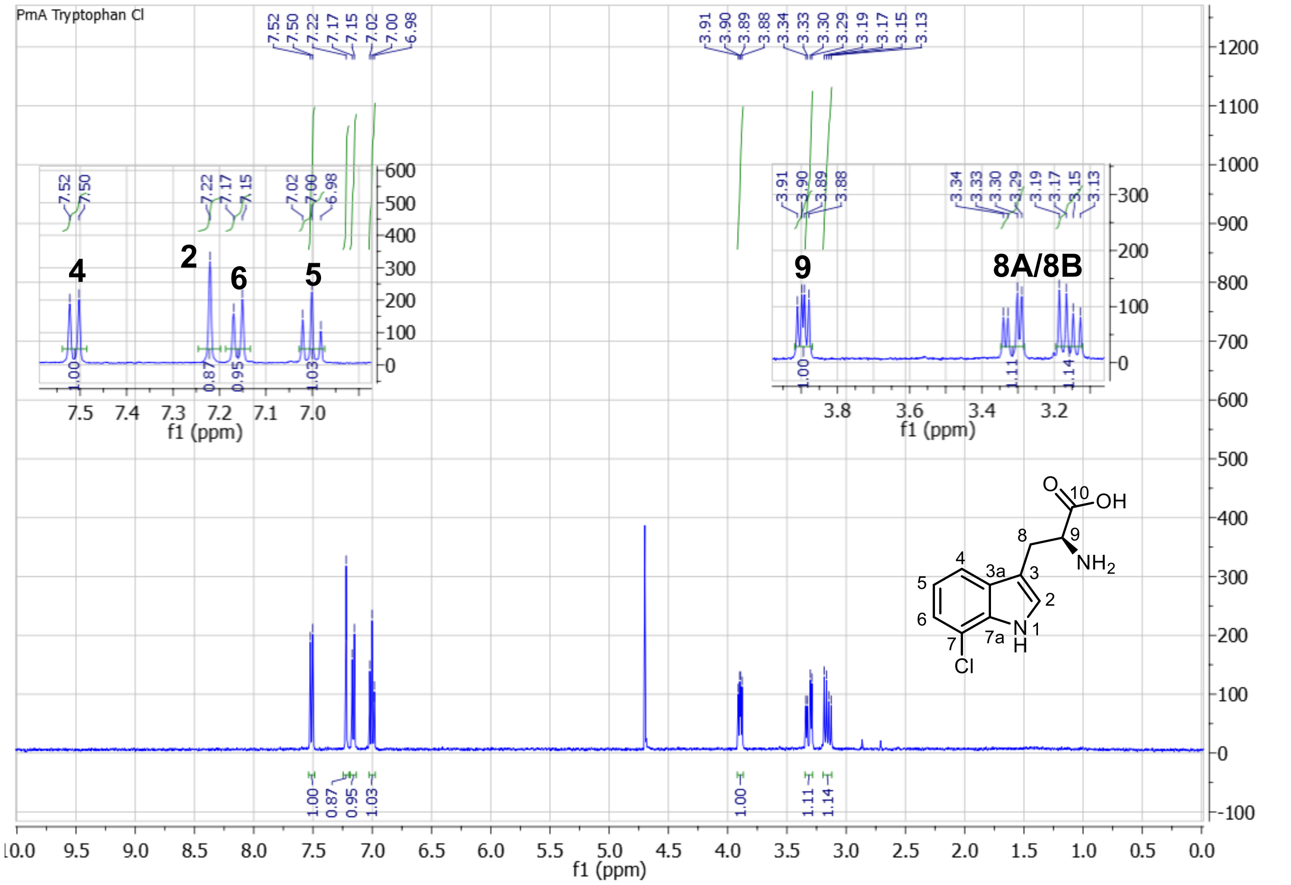
PmA Tryptophan Cl
4
2
6
5
9
8A/8B
f1 (ppm)

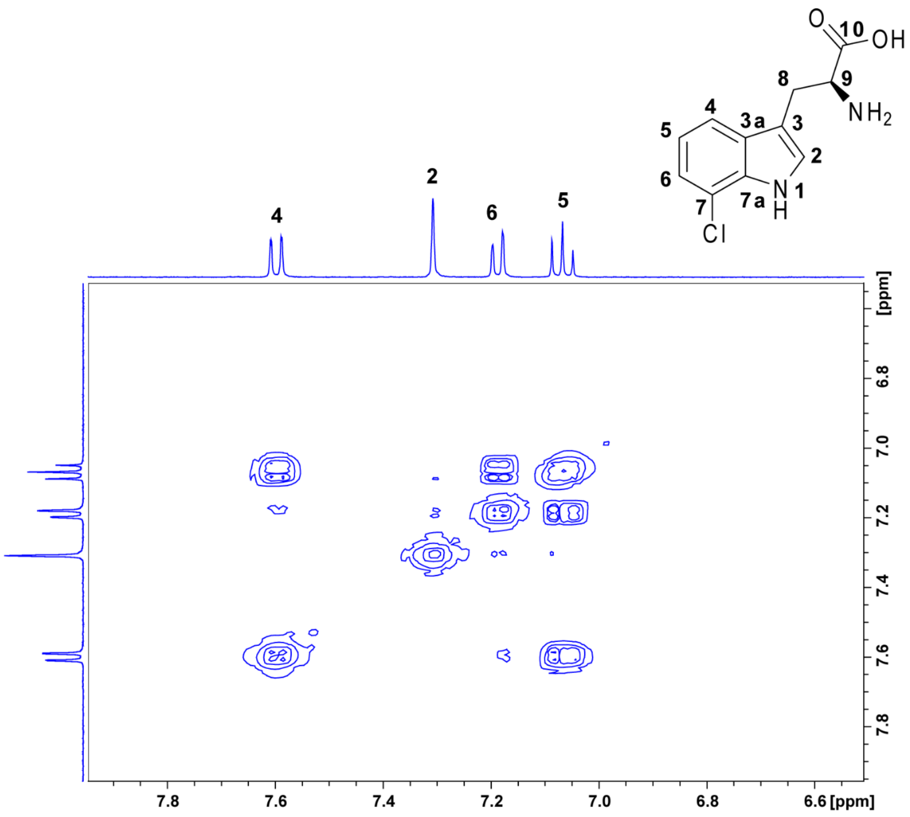

COSY of 7-chlorotryptophan

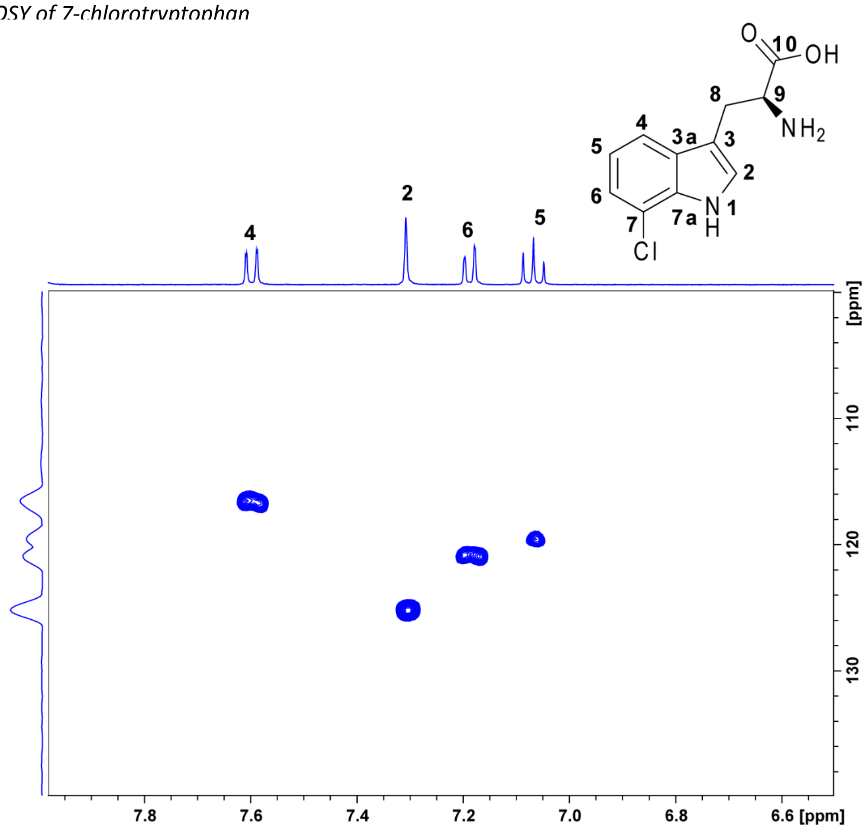

HMQC of 7-chlorotryptophan

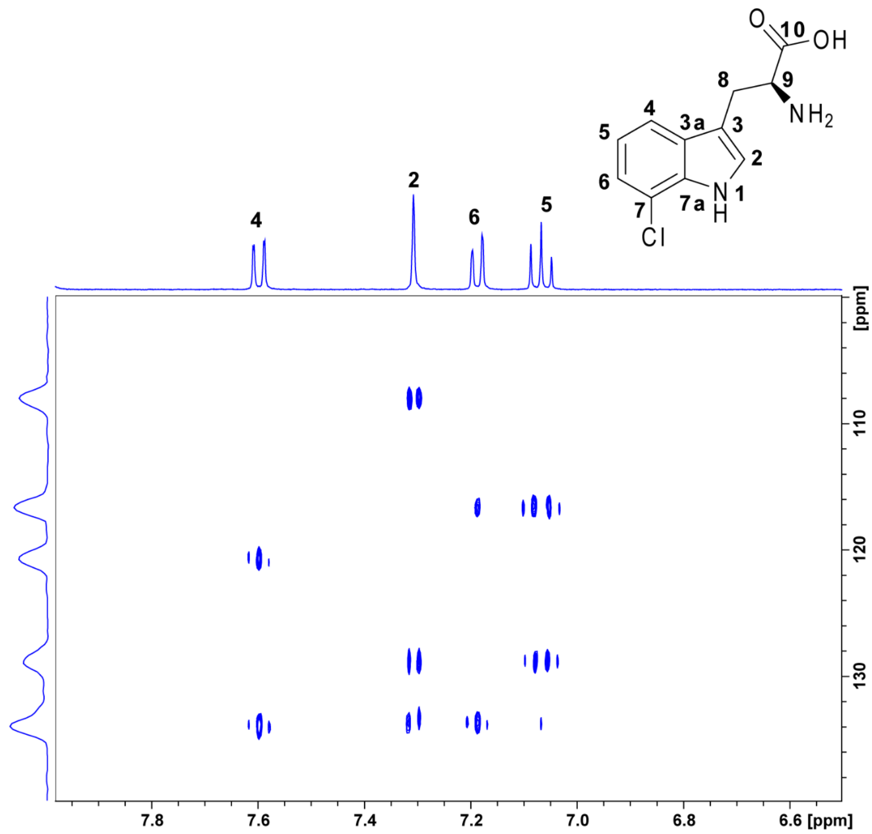

HMBC of 7-chlorotryptophan

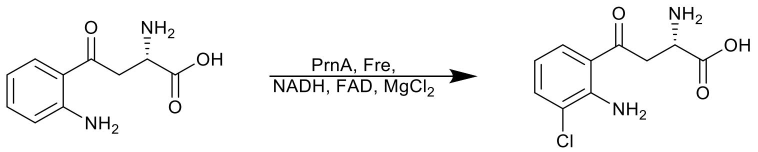
PrnA, Fre,
NADH, FAD, $MgCl_2$

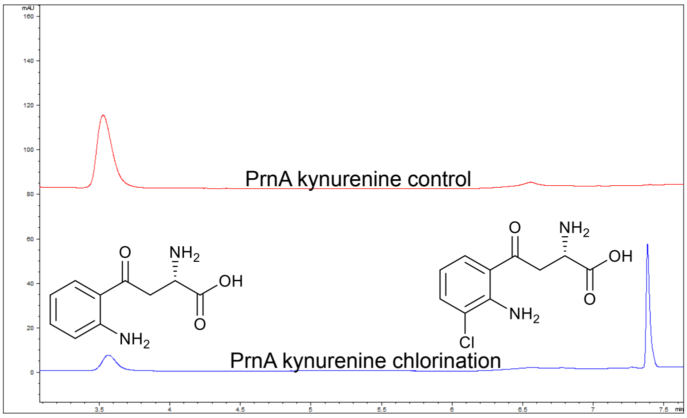
PrnA kynurenine control
PrnA kynurenine chlorination

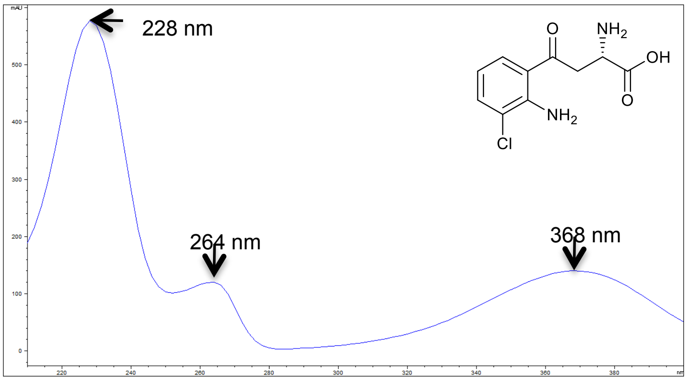
228 nm
264 nm
368 nm

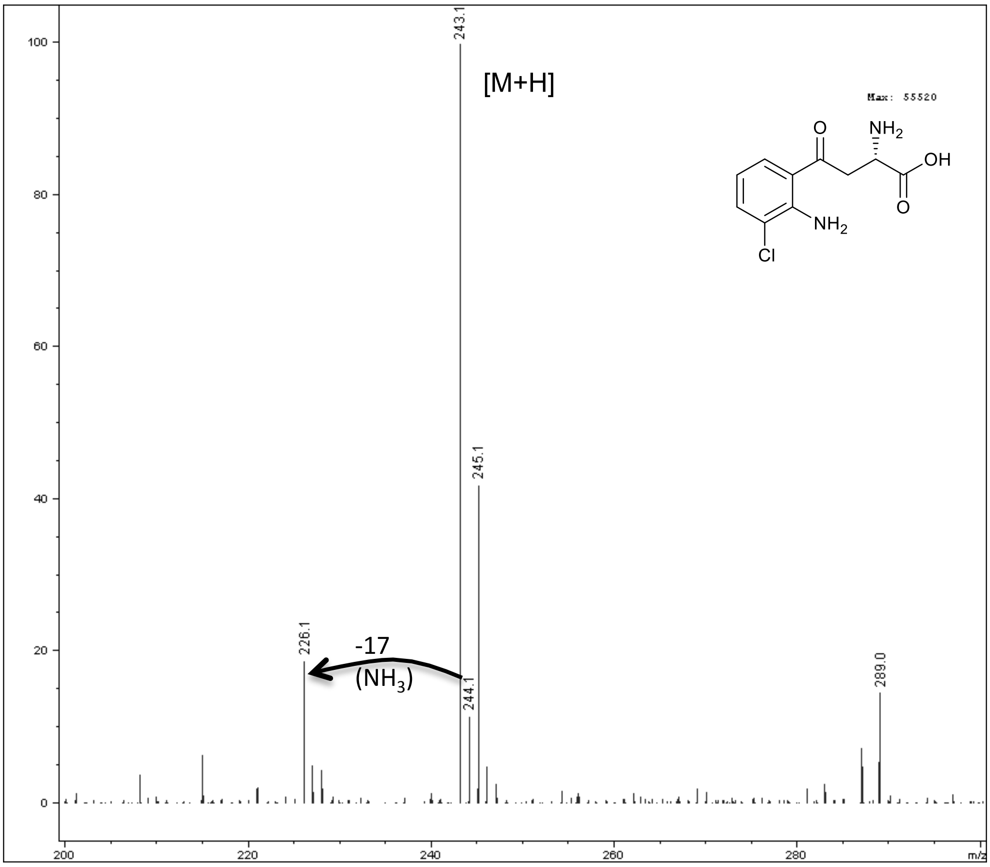
[M+H]
Max: 55520
243.1
245.1
226.1
244.1
289.0
-17
(NH_3)
m/z

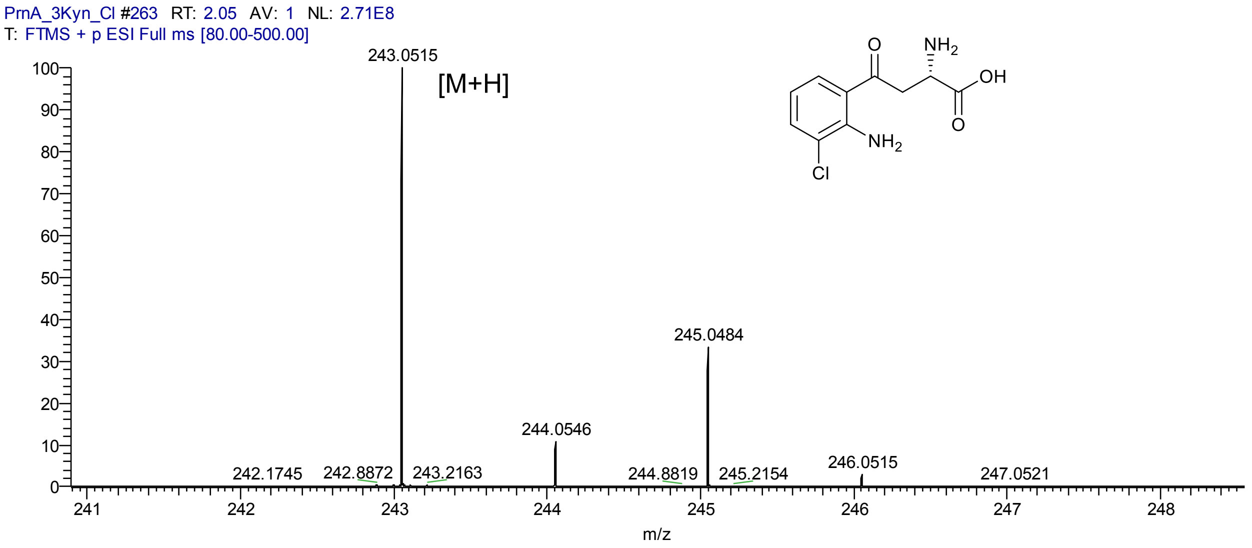
PmA_3Kyn_Cl #263 RT: 2.05 AV: 1 NL: 2.71E8
T: FTMS + p ESI Full ms [80.00-500.00]
243.0515
[M+H]
245.0484
244.0546
246.0515
m/z

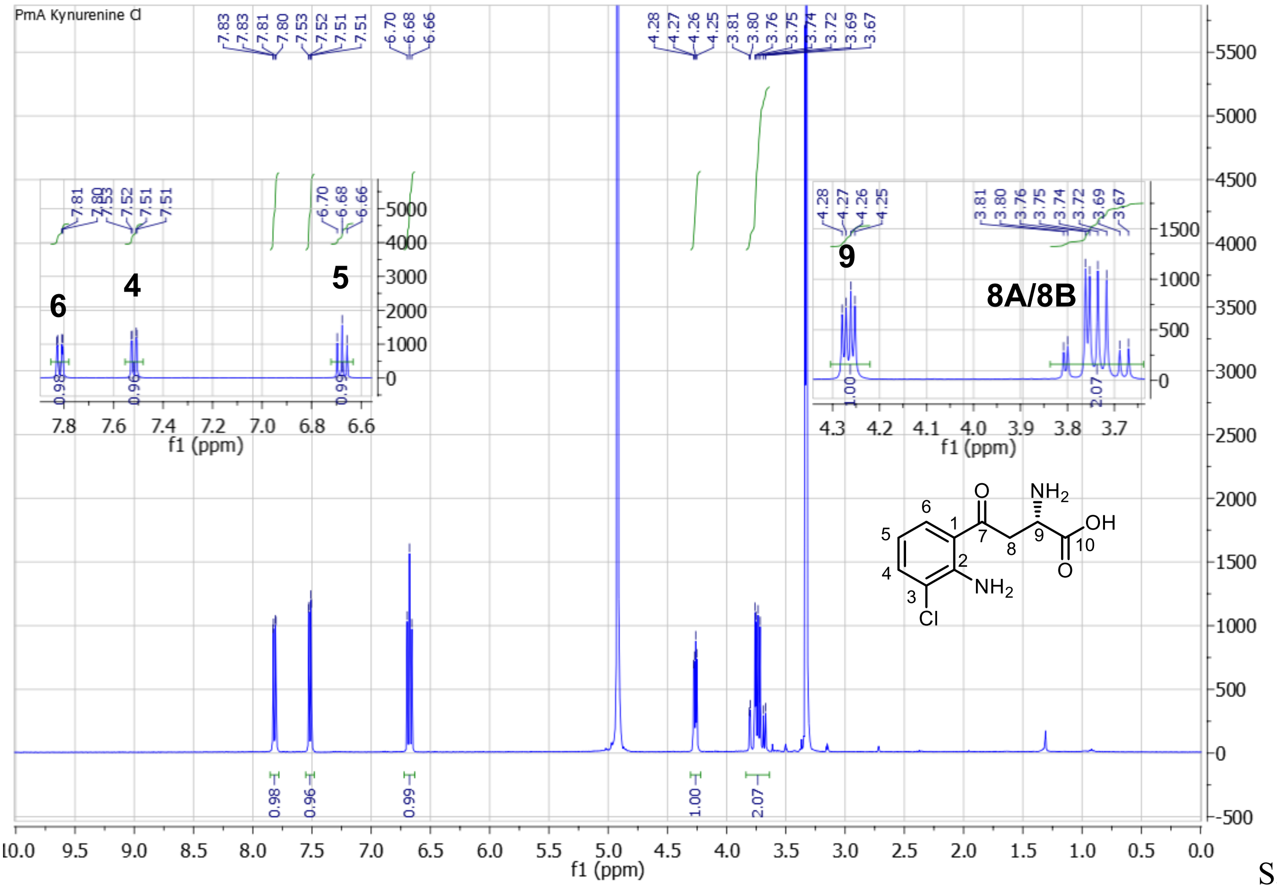
PmA Kynurenine Cl
6
4
5
9
8A/8B
f1 (ppm)

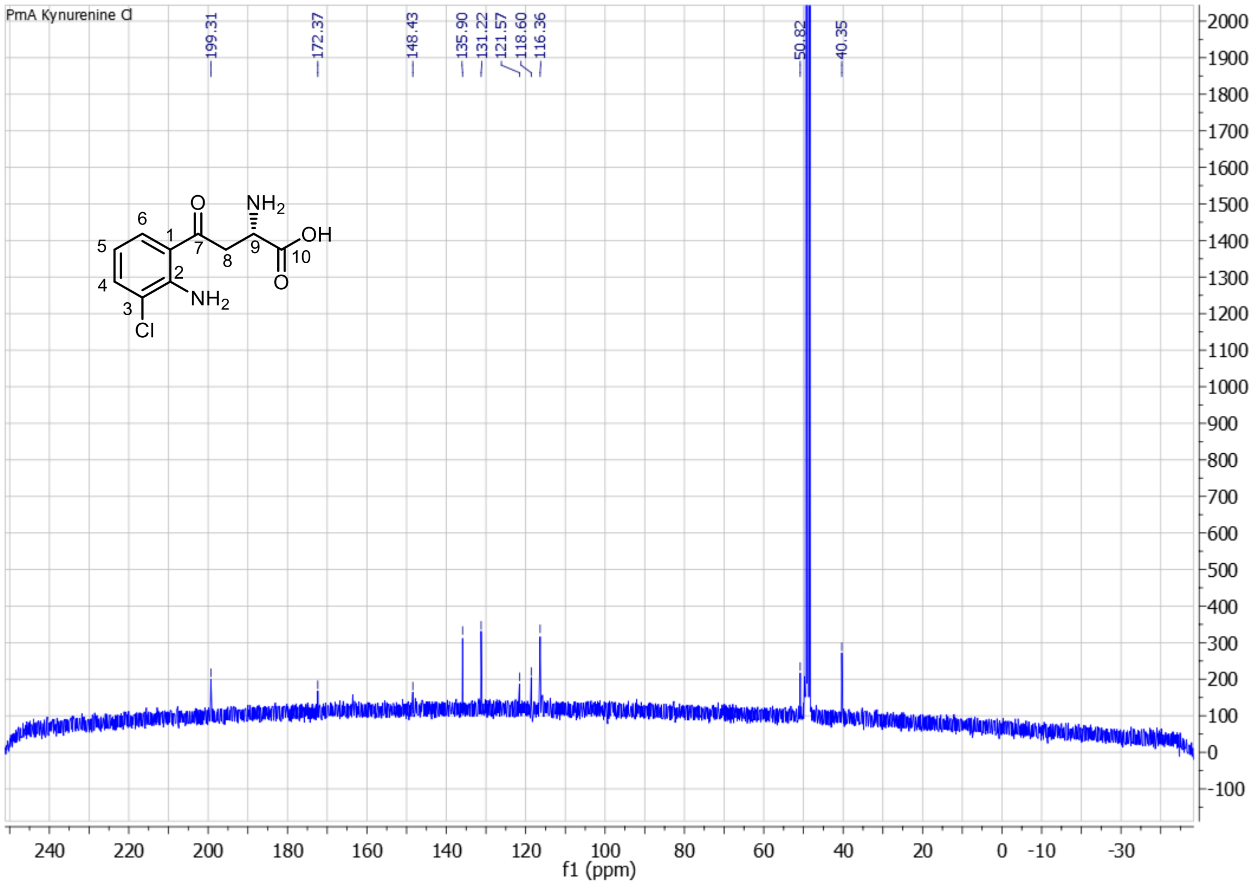

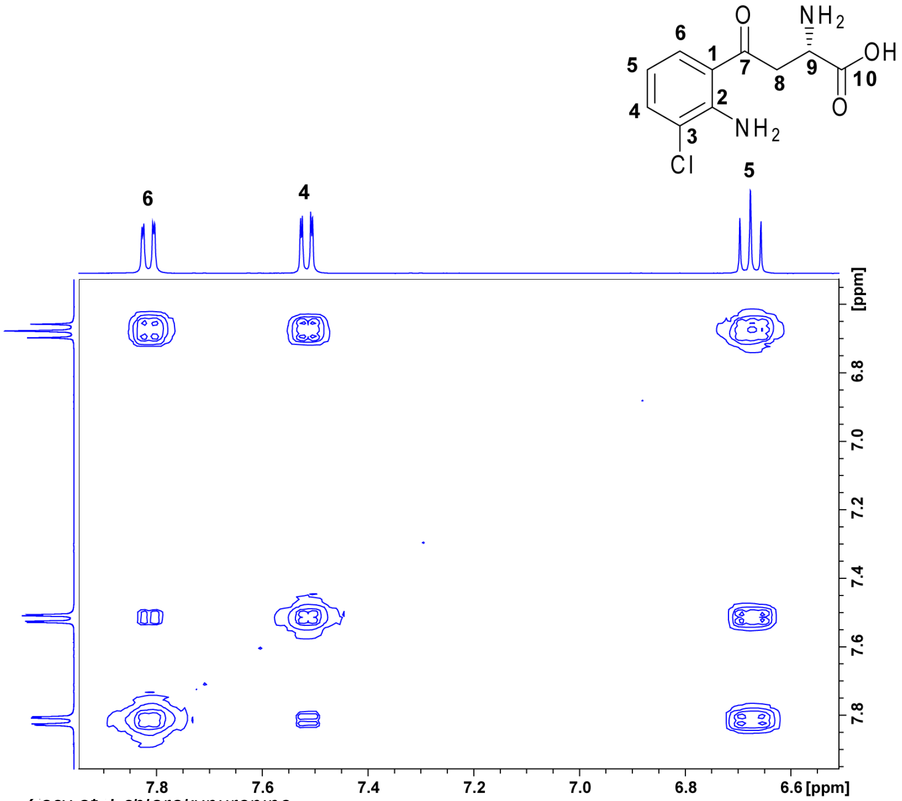

Cosy of 3-chlorokynurenine

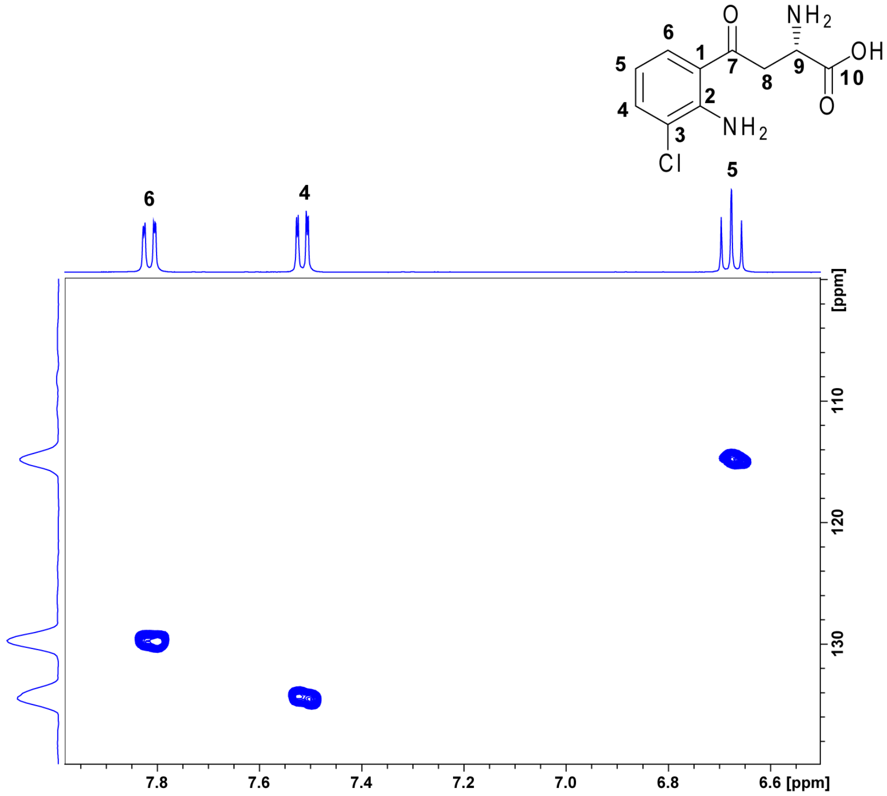

HMQC of 3-chlorokynurenine

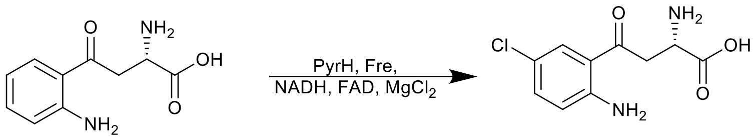
O
NH2
OH
O
NH2
PyrH, Fre,
NADH, FAD, MgCl2
Cl
O
NH2
OH
O
NH2

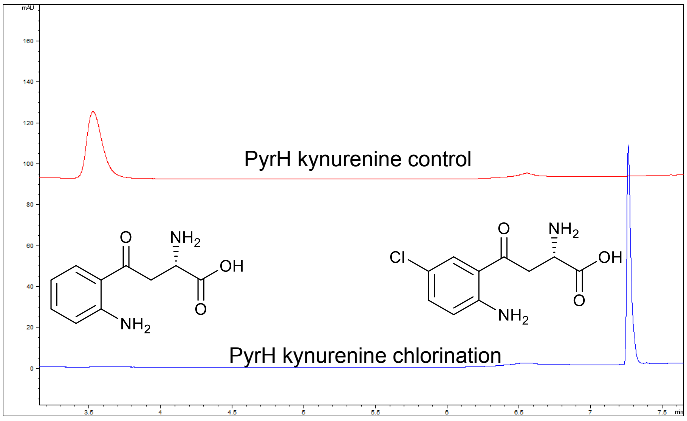
PyrH kynurenine control
O
NH2
OH
O
NH2
Cl
O
NH2
OH
O
NH2
PyrH kynurenine chlorination

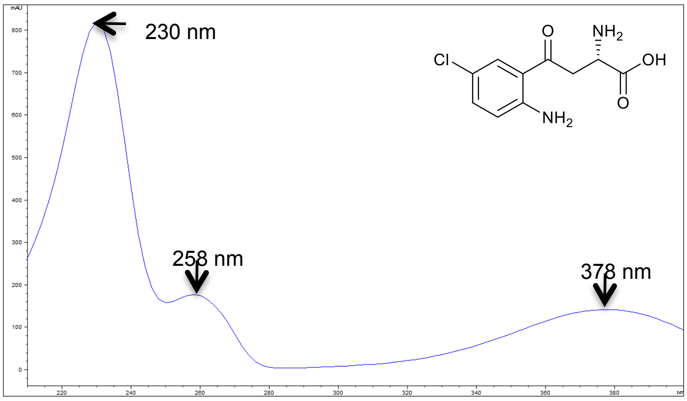
230 nm
258 nm
378 nm
Cl
O
NH2
OH
O
NH2

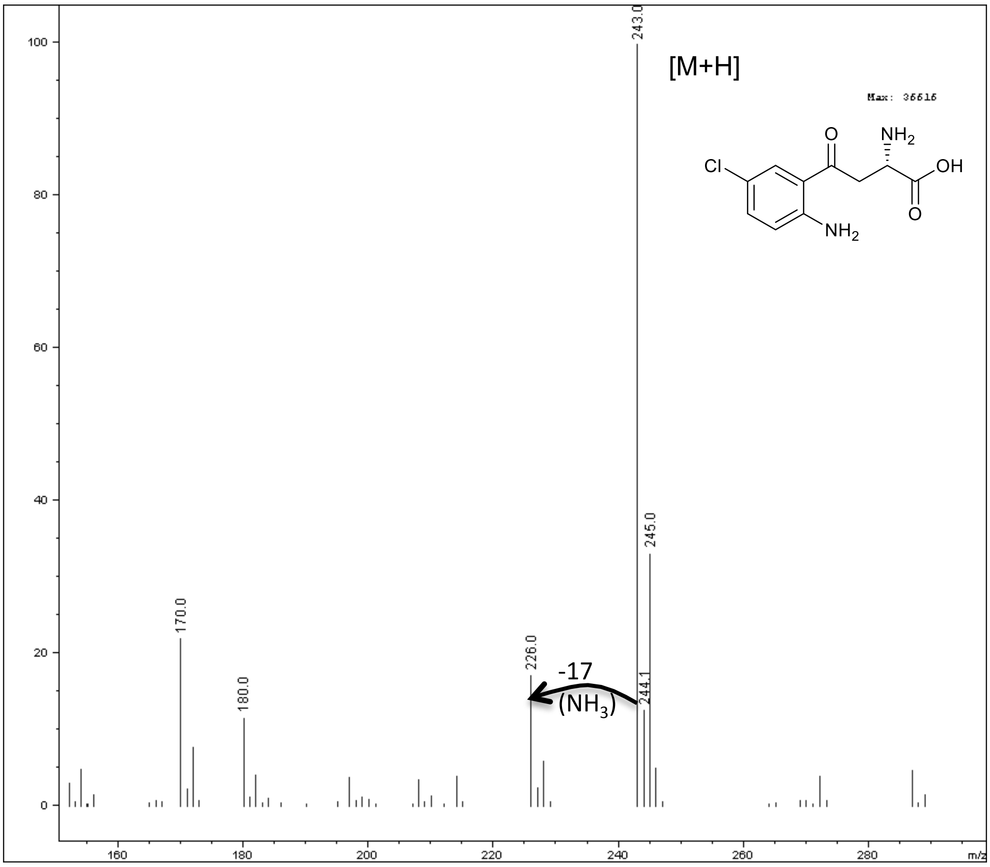
100
80
60
40
20
0
160
180
200
220
240
260
280
m/z
243.0
245.0
170.0
180.0
226.0
244.1
-17
(NH3)
[M+H]
Cl
O
NH2
OH
O
NH2

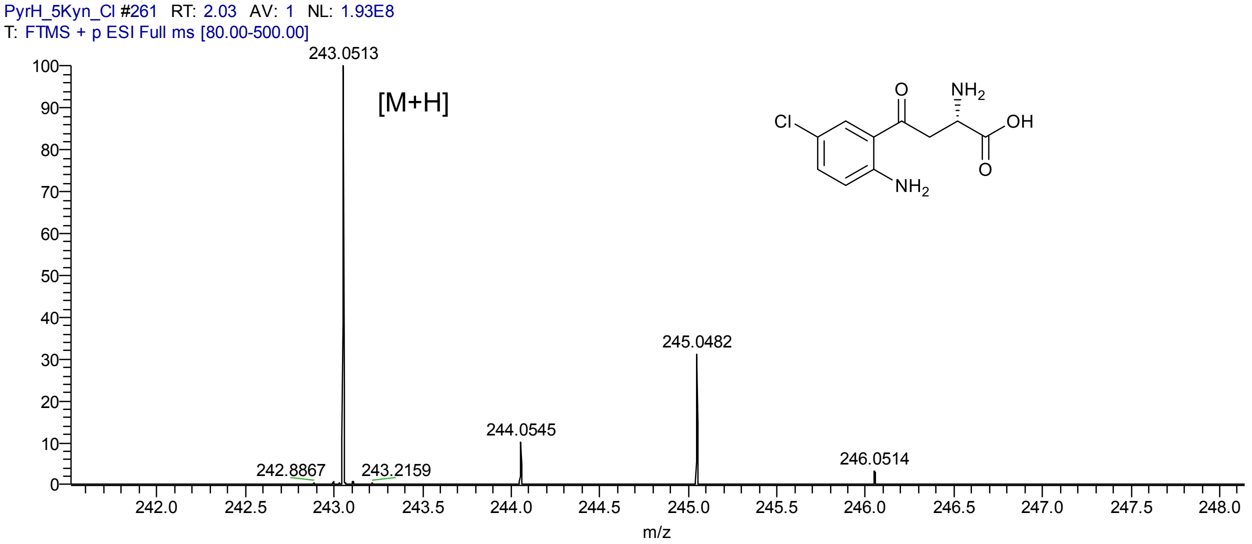
PyrH_5Kyn_Cl #261 RT: 2.03 AV: 1 NL: 1.93E8
T: FTMS + p ESI Full ms [80.00-500.00]
243.0513
[M+H]
245.0482
244.0545
246.0514
242.8867
243.2159
m/z
Cl
O
NH2
OH
O
NH2

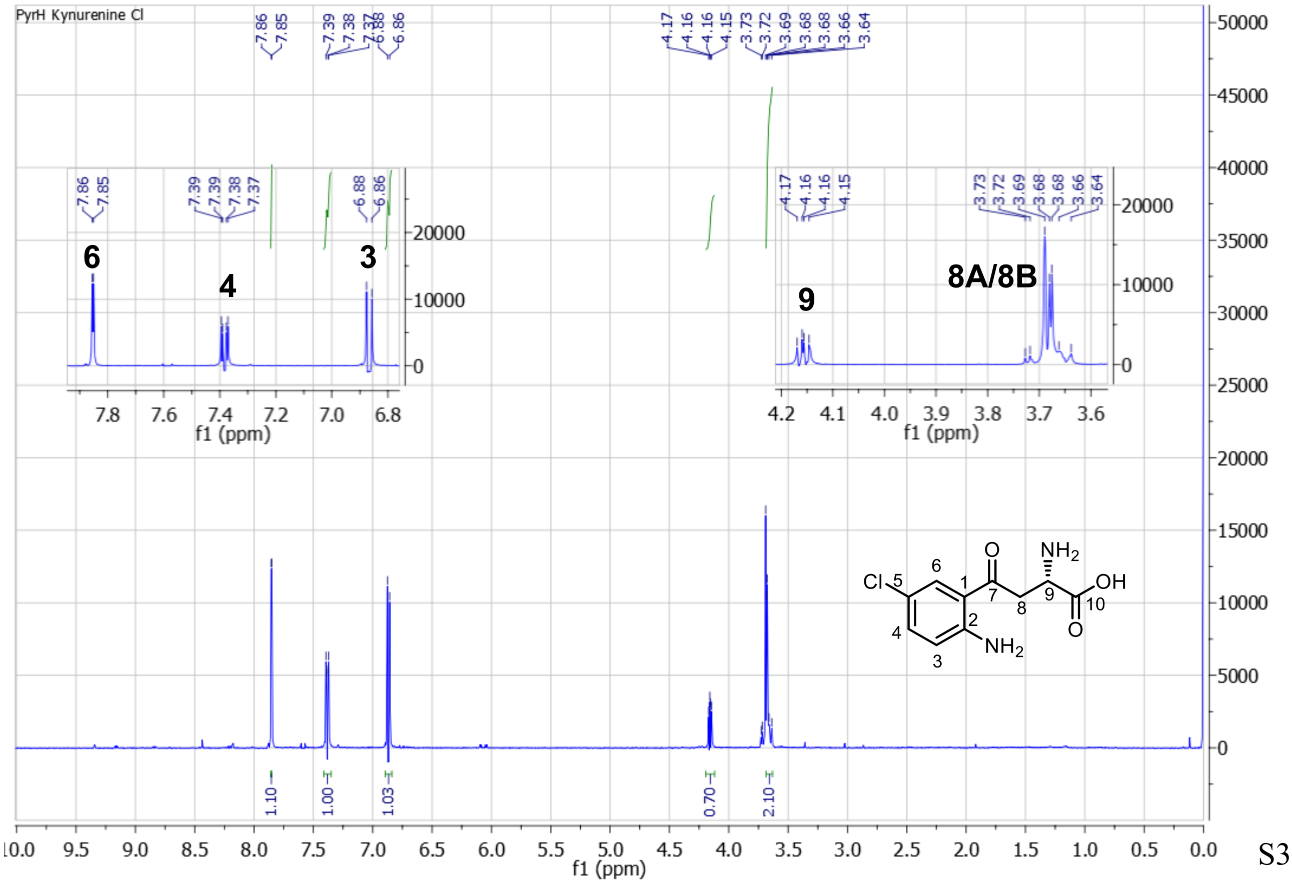
PyrH Kynurenine Cl
6
4
3
9
8A/8B
f1 (ppm)
1.10
1.00
1.03
0.70
2.10
Cl
5
6
1
2
3
4
7
8
9
10
O
NH2
OH
O
NH2

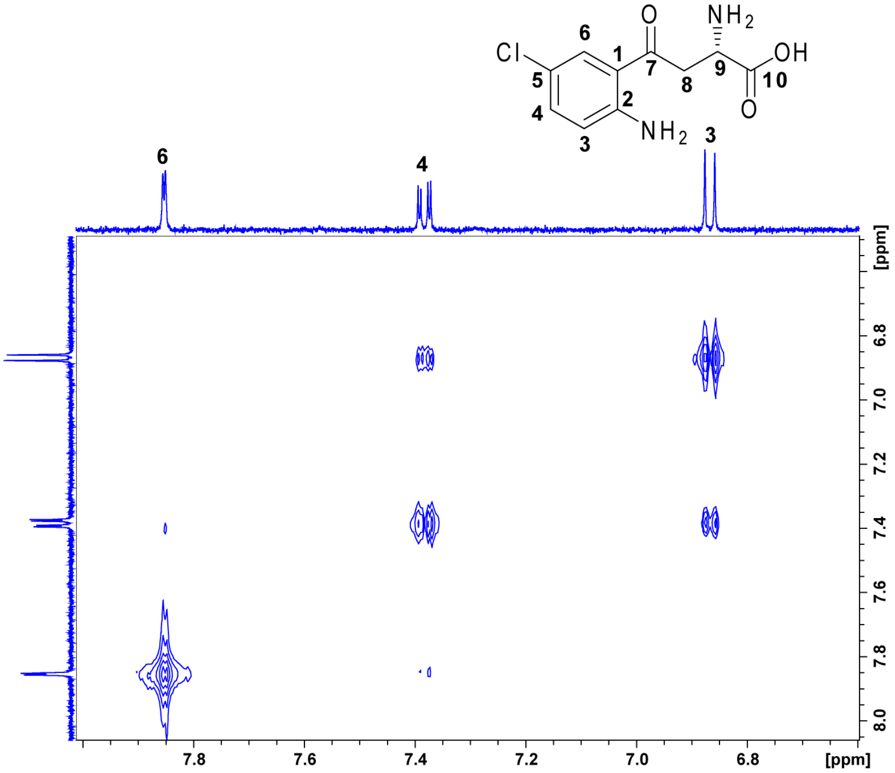

COSY of 5-chlorokynurenine

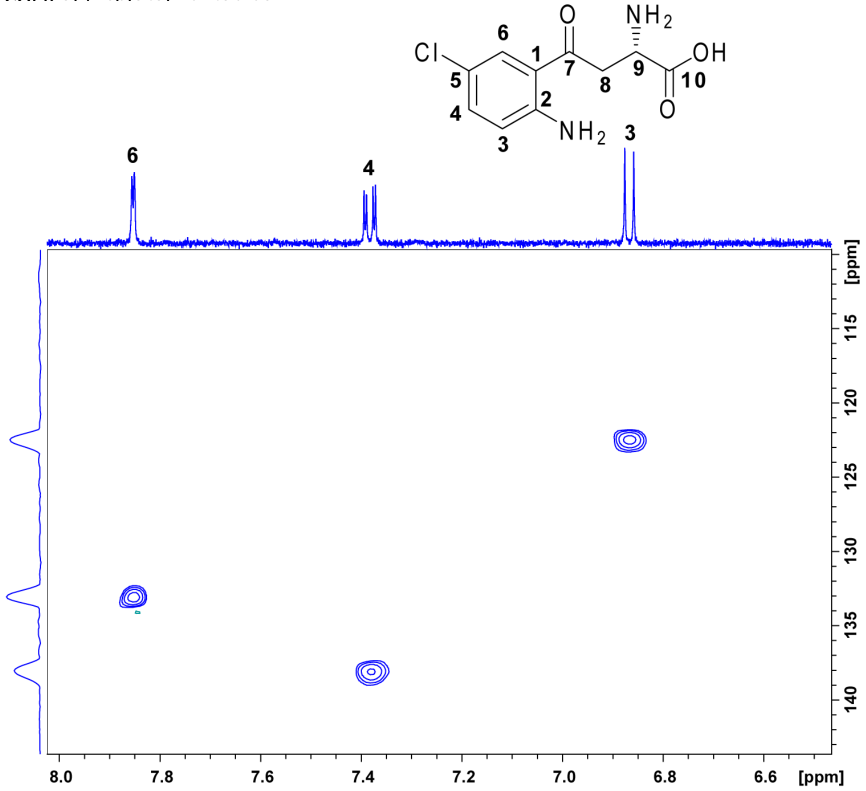

HSQC of 5-chlorokynurenine

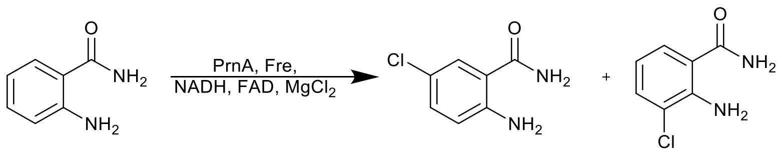
PrnA, Fre,
NADH, FAD, MgCl2
+

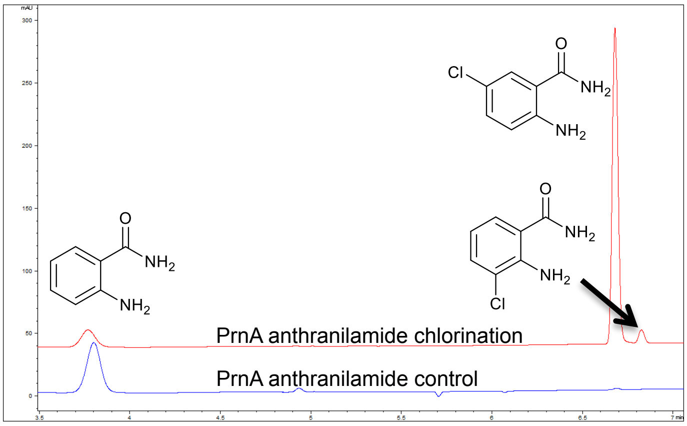
PrnA anthranilamide chlorination
PrnA anthranilamide control

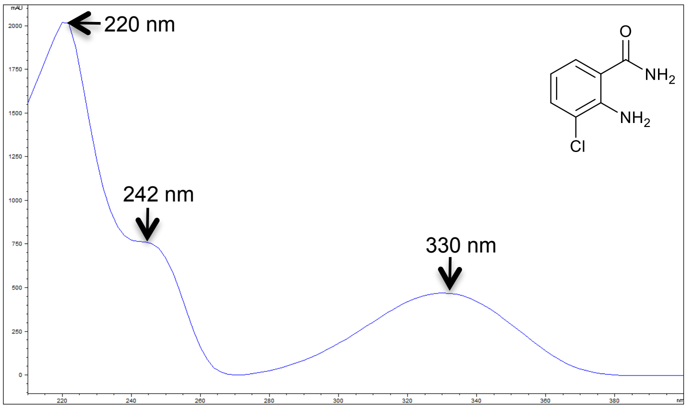
220 nm
242 nm
330 nm

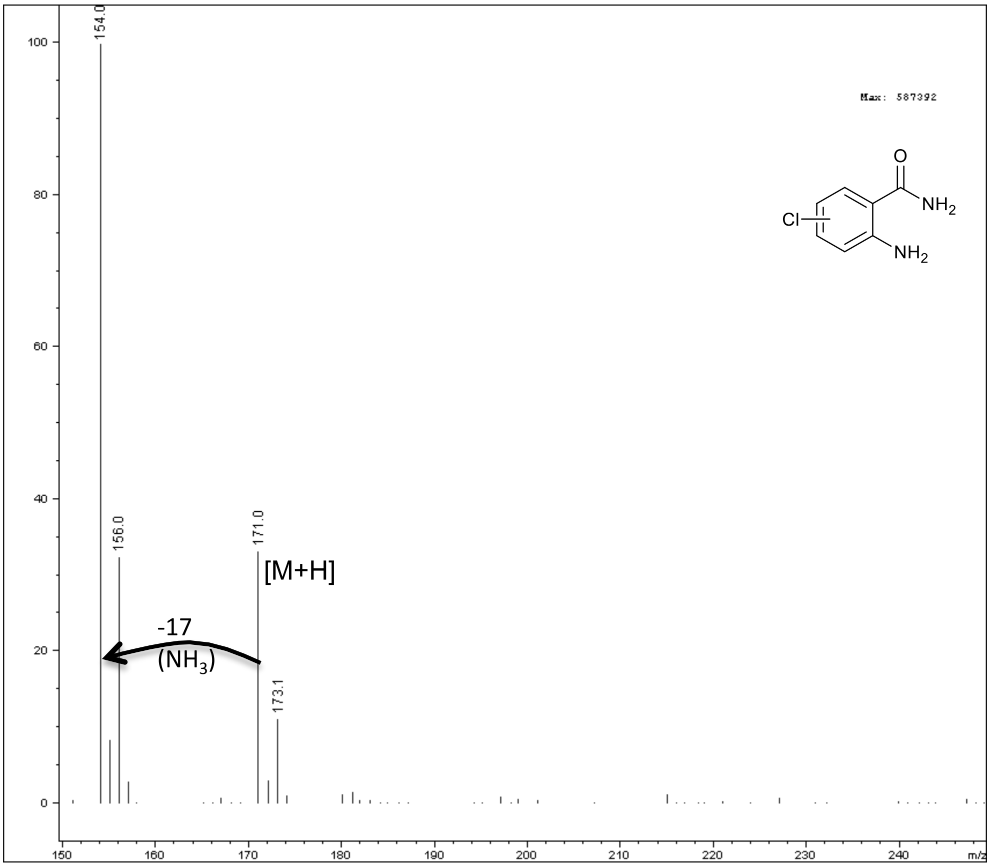
154.0
156.0
171.0
173.1
[M+H]
-17
(NH3)
Max: 587392

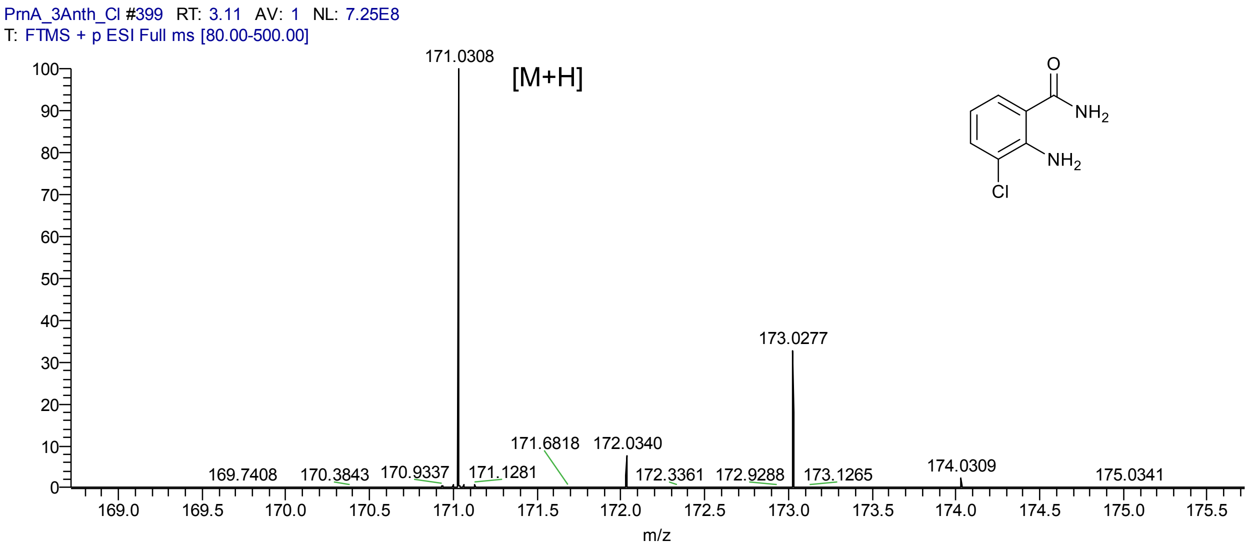

PmA_3Anth_Cl #399 RT: 3.11 AV: 1 NL: 7.25E8
T: FTMS + p ESI Full ms [80.00-500.00]
171.0308
[M+H]
173.0277
172.0340
171.6818
174.0309
175.0341
m/z

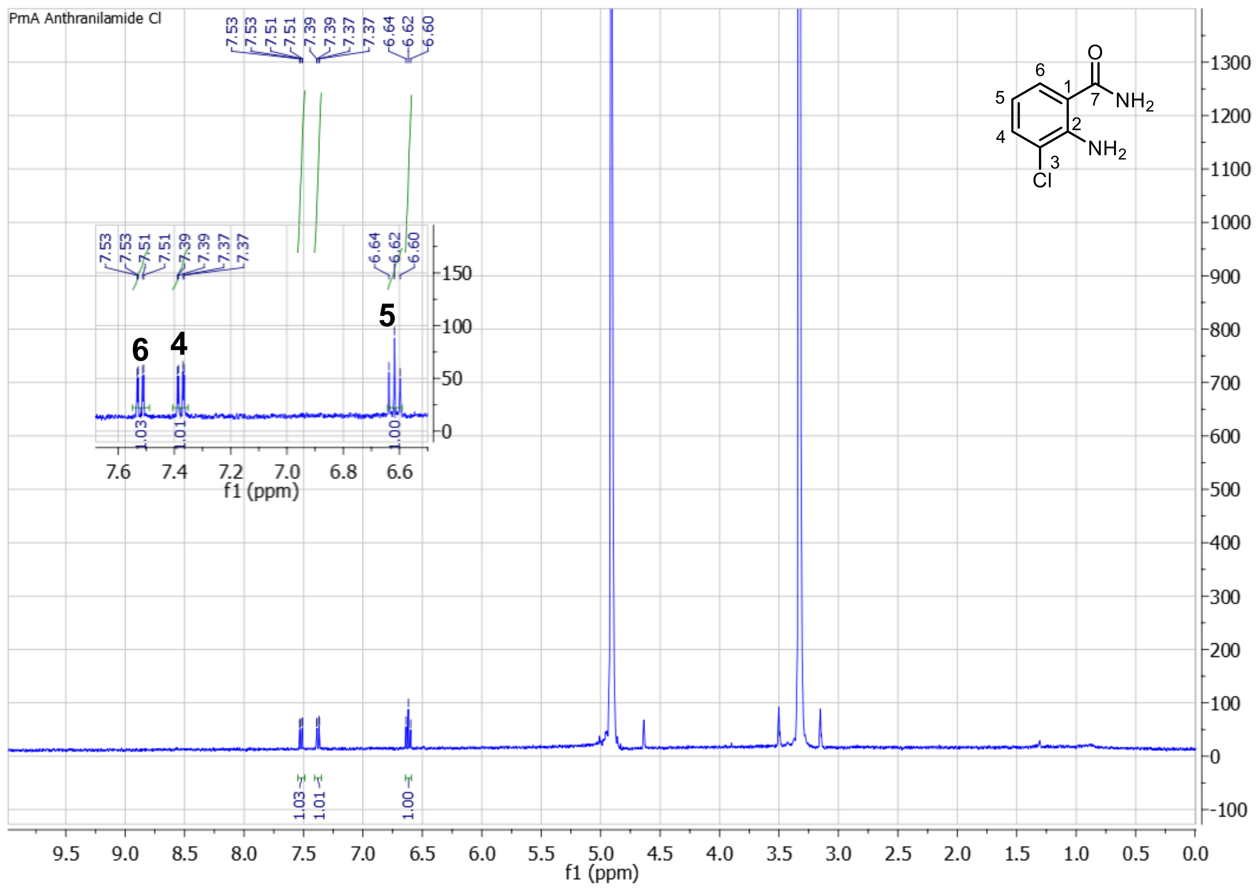

PmA Anthranilamide Cl
7.53
7.53
7.51
7.51
7.39
7.39
7.37
7.37
6.64
6.62
6.60
6
4
5
1.03
1.01
1.00
f1 (ppm)

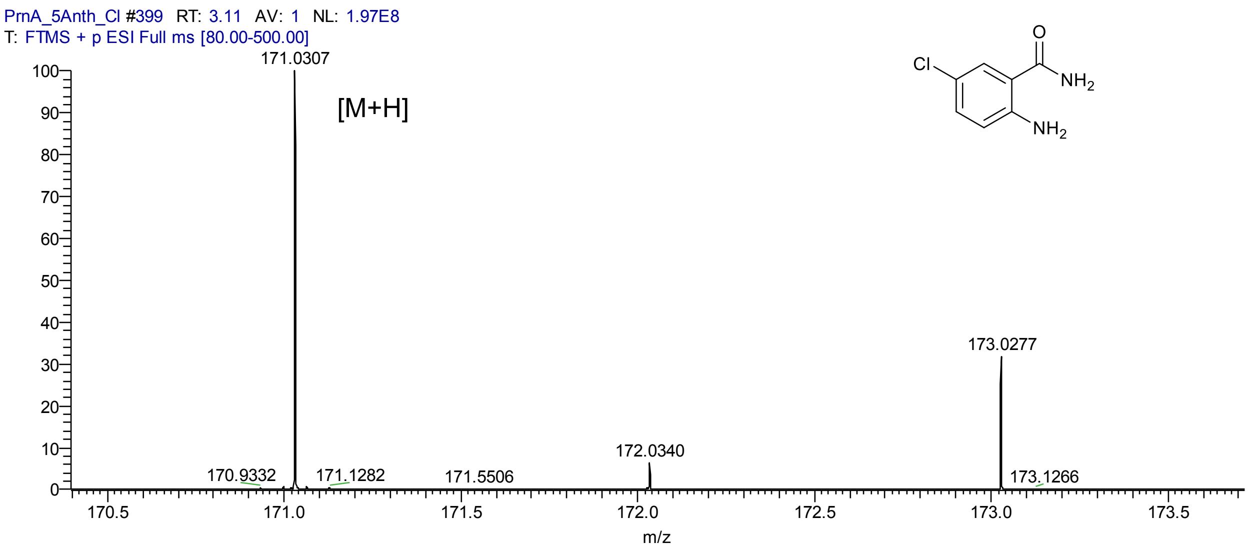

PrnA_5Anth_Cl #399 RT: 3.11 AV: 1 NL: 1.97E8
T: FTMS + p ESI Full ms [80.00-500.00]
171.0307
[M+H]
173.0277
172.0340
170.9332
171.1282
171.5506
173.1266
m/z
Cl
O
NH2
NH2

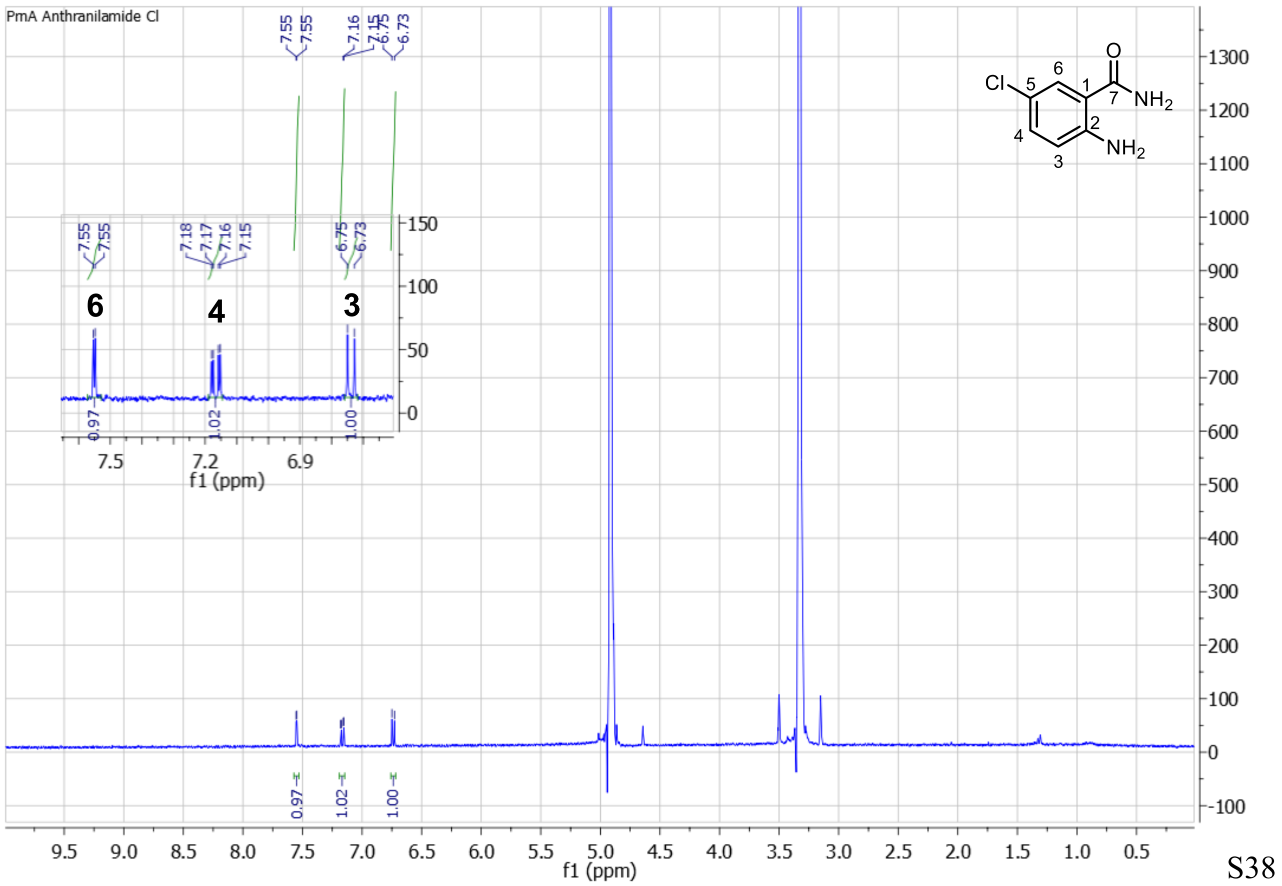

PrnA Anthranilamide Cl
6
4
3
f1 (ppm)
Cl
O
NH2
NH2

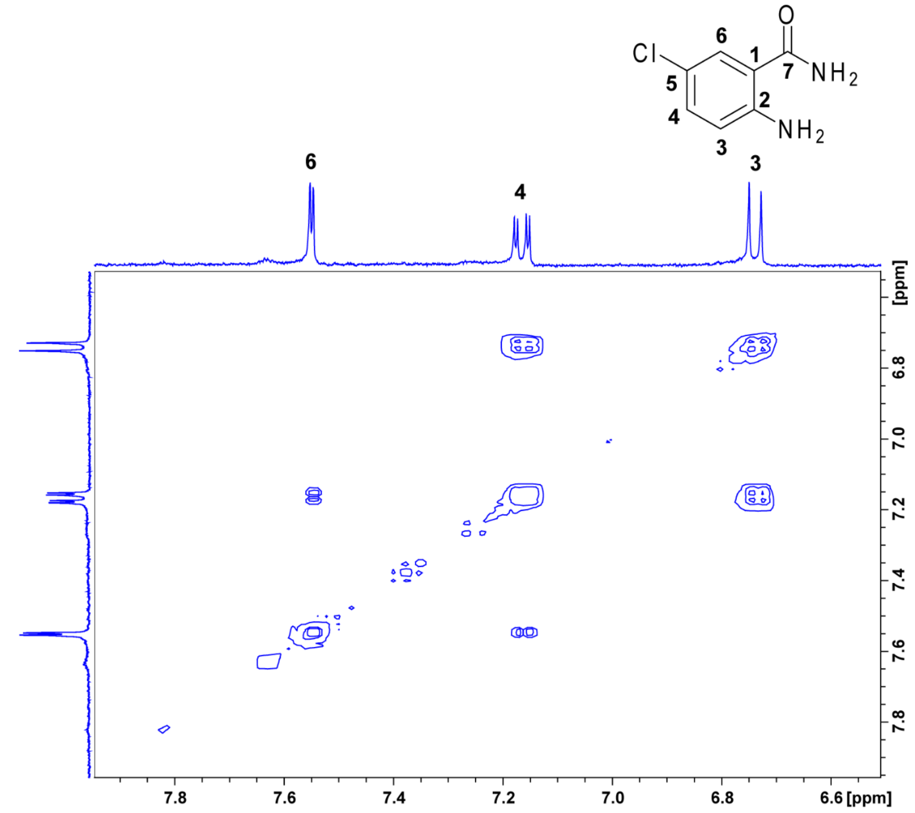

COSY of 5-chloroanthranilamide

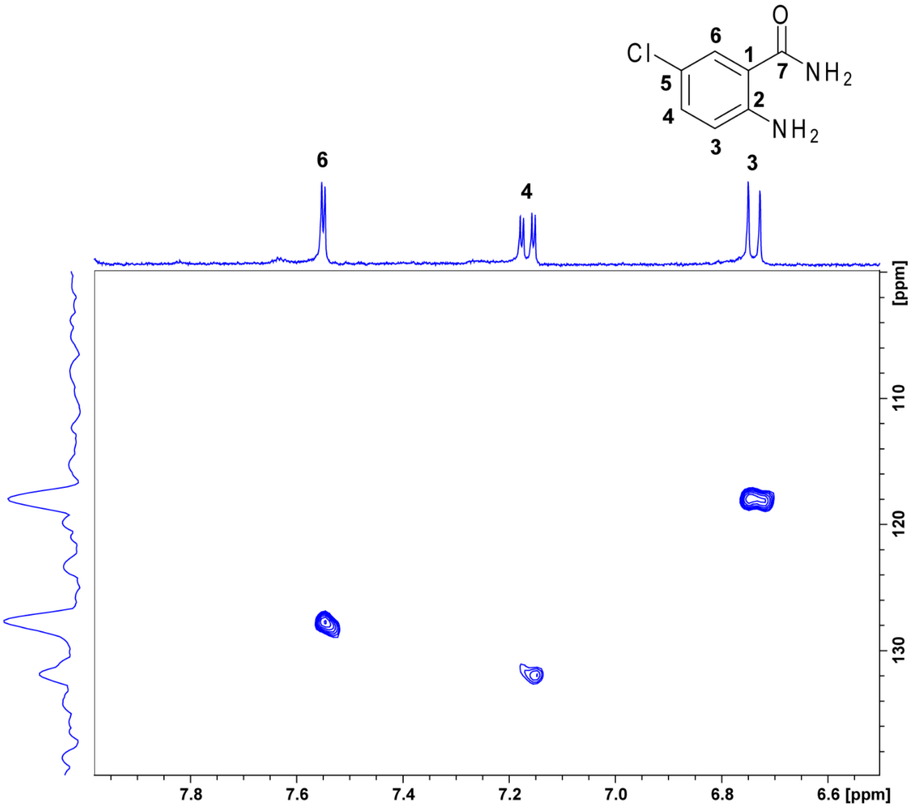

HMQC of 5-chloroanthranilamide

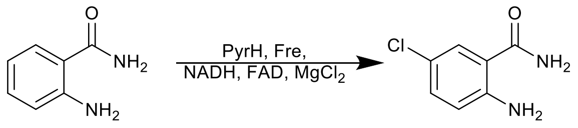
PyrH, Fre,
NADH, FAD, MgCl2
Cl
O
NH2
NH2

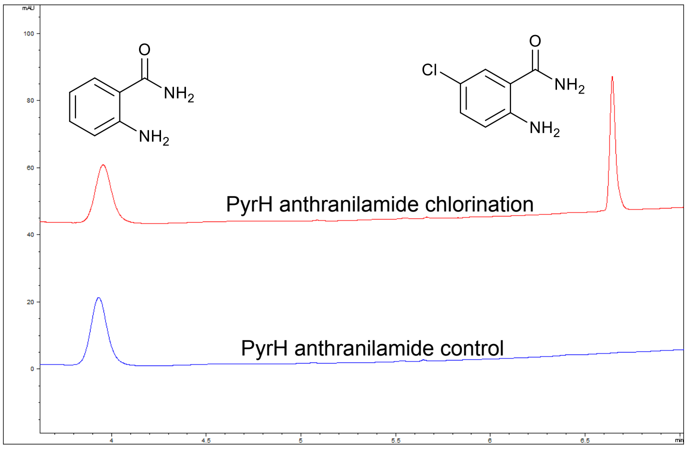
PyrH anthranilamide chlorination
PyrH anthranilamide control
Cl
O
NH2
NH2

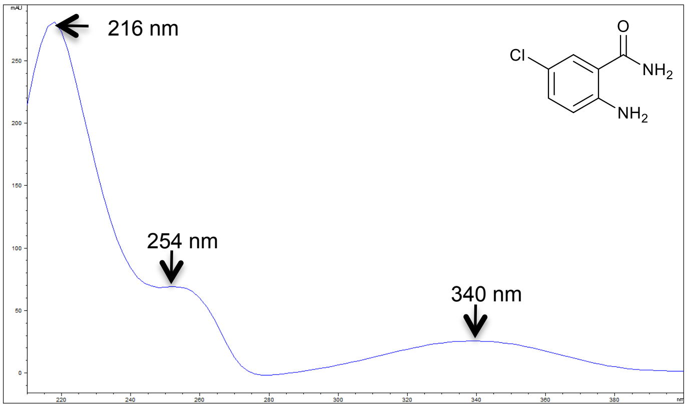
216 nm
254 nm
340 nm
Cl
O
NH2
NH2

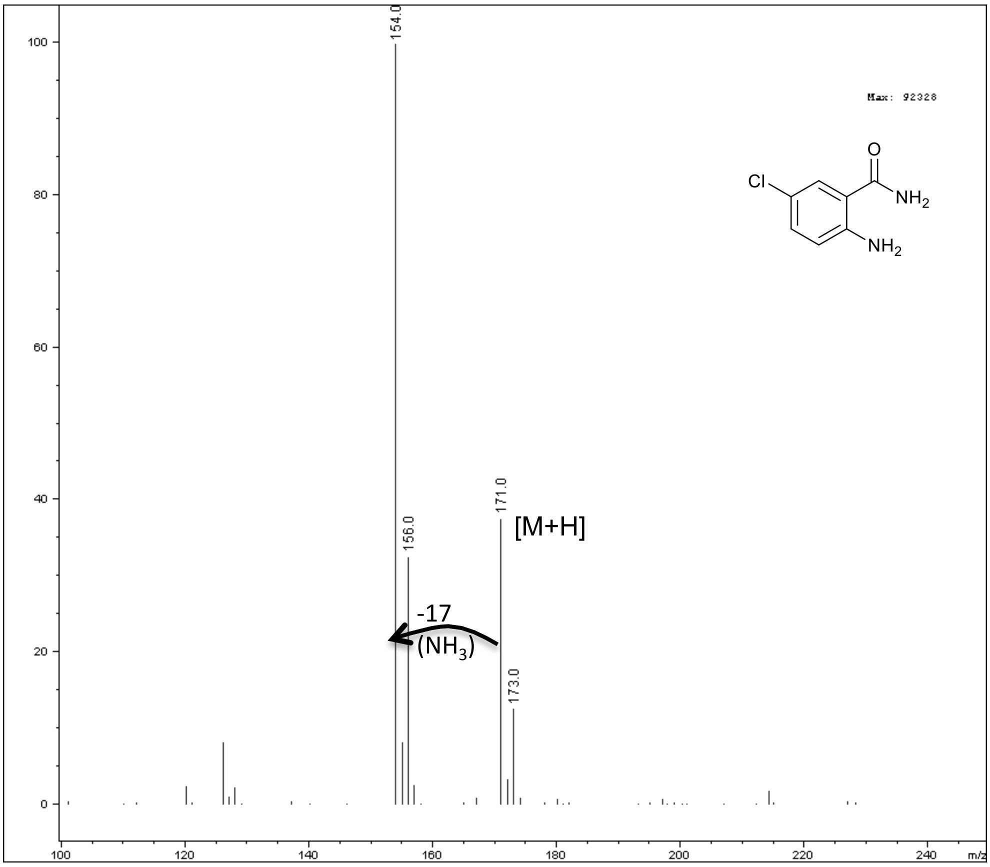
154.0
156.0
171.0
173.0
[M+H]
-17
(NH3)
Max: 92328
Cl
O
NH2
NH2
m/z

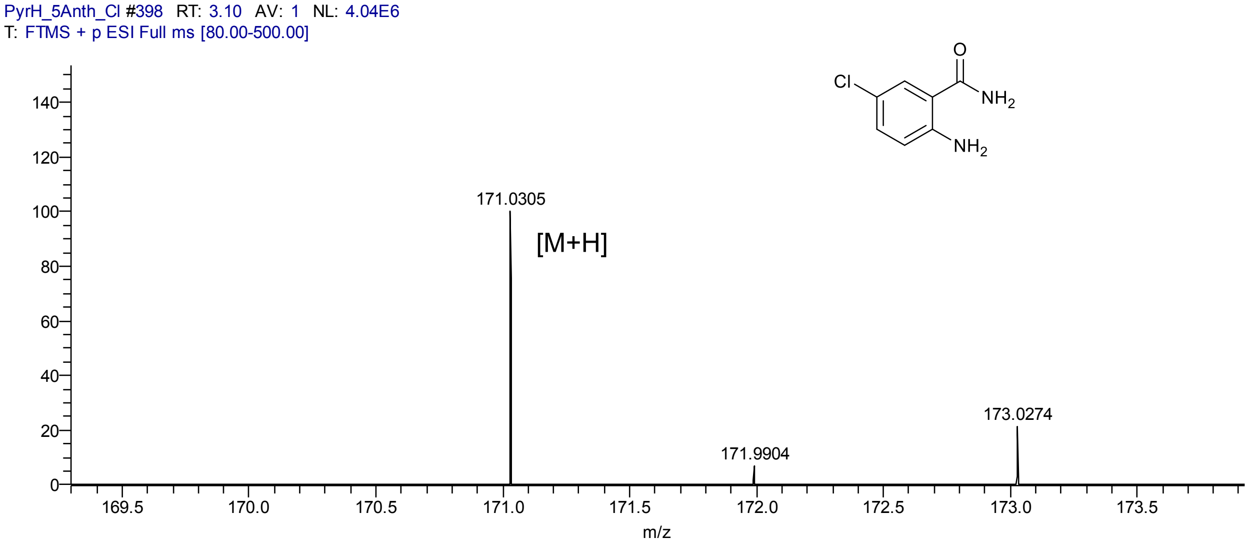
PyrH_5Anth_Cl #398 RT: 3.10 AV: 1 NL: 4.04E6
T: FTMS + p ESI Full ms [80.00-500.00]
171.0305
[M+H]
171.9904
173.0274
m/z
Cl
O
NH2
NH2

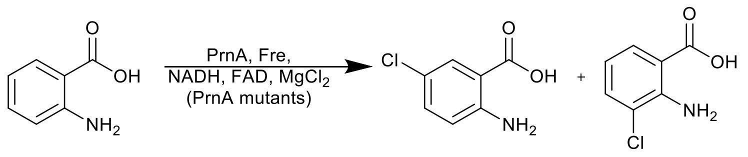
PrnA, Fre,
NADH, FAD, $MgCl_2$
(PrnA mutants)
+

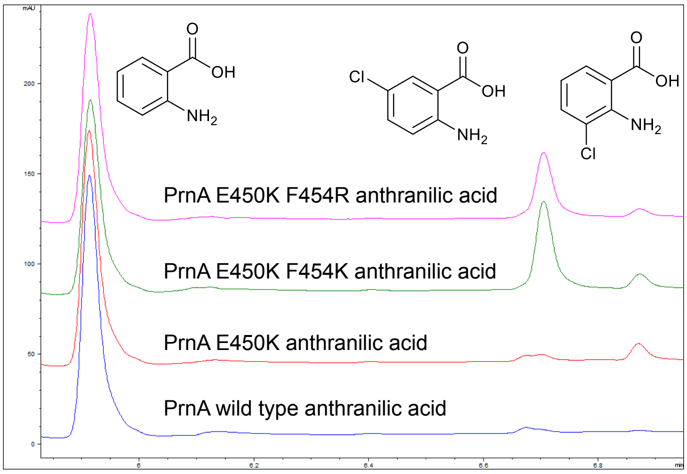
PrnA E450K F454R anthranilic acid
PrnA E450K F454K anthranilic acid
PrnA E450K anthranilic acid
PrnA wild type anthranilic acid

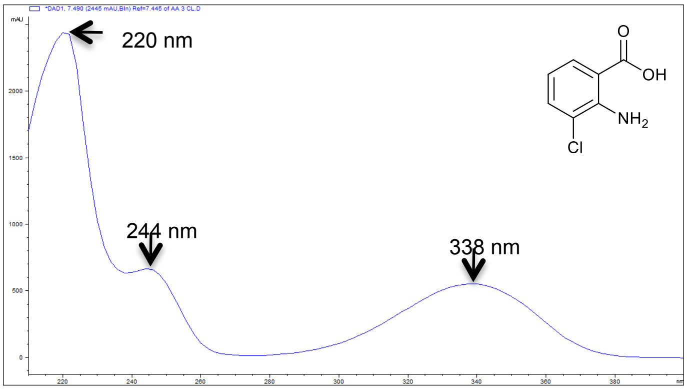
220 nm
244 nm
338 nm

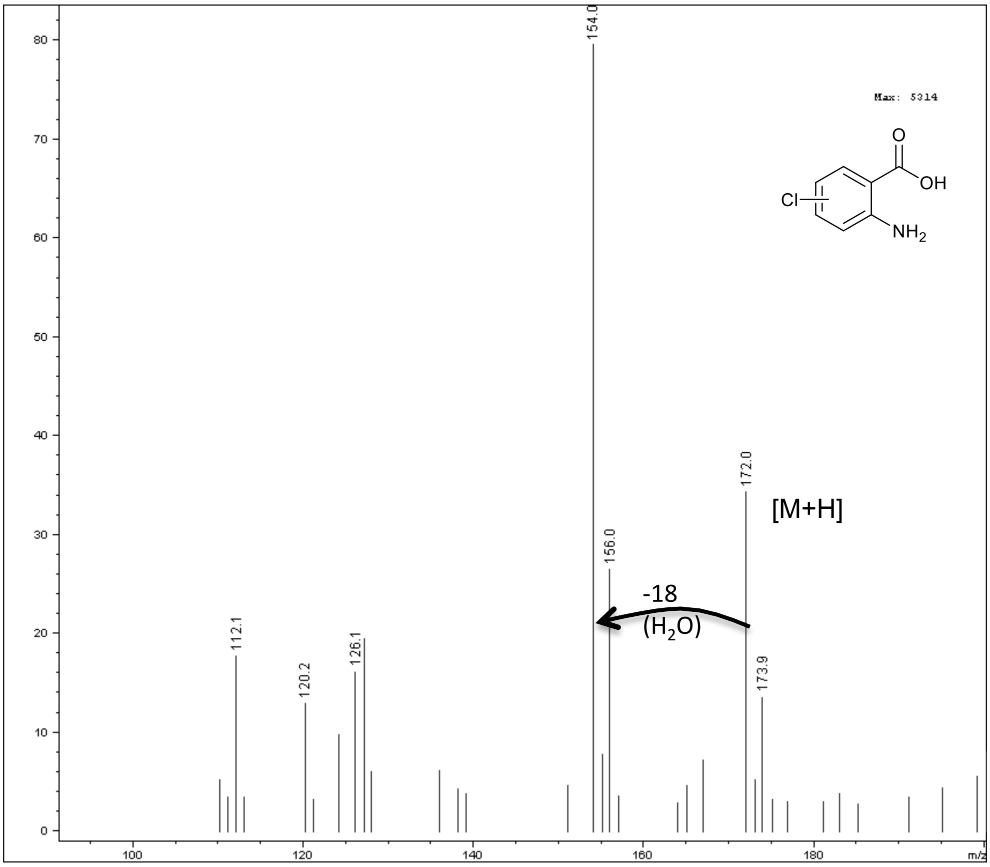
154.0
172.0
[M+H]
156.0
-18
(H_2O)
173.9
112.1
120.2
126.1
Max: 5314

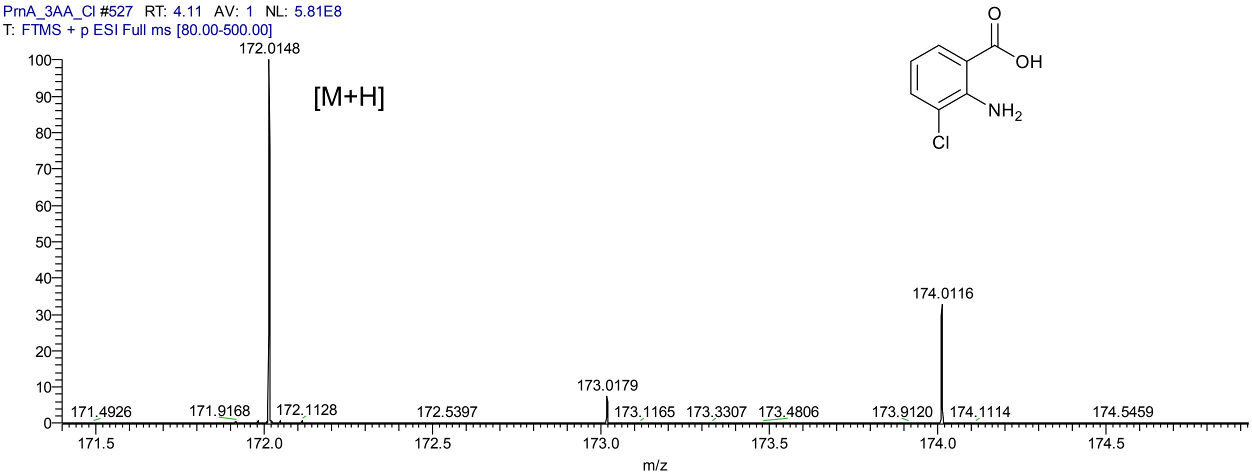
PrnA_3AA_Cl #527 RT: 4.11 AV: 1 NL: 5.81E8
T: FTMS + p ESI Full ms [80.00-500.00]
172.0148
[M+H]
174.0116
173.0179
m/z
OH
NH2
Cl

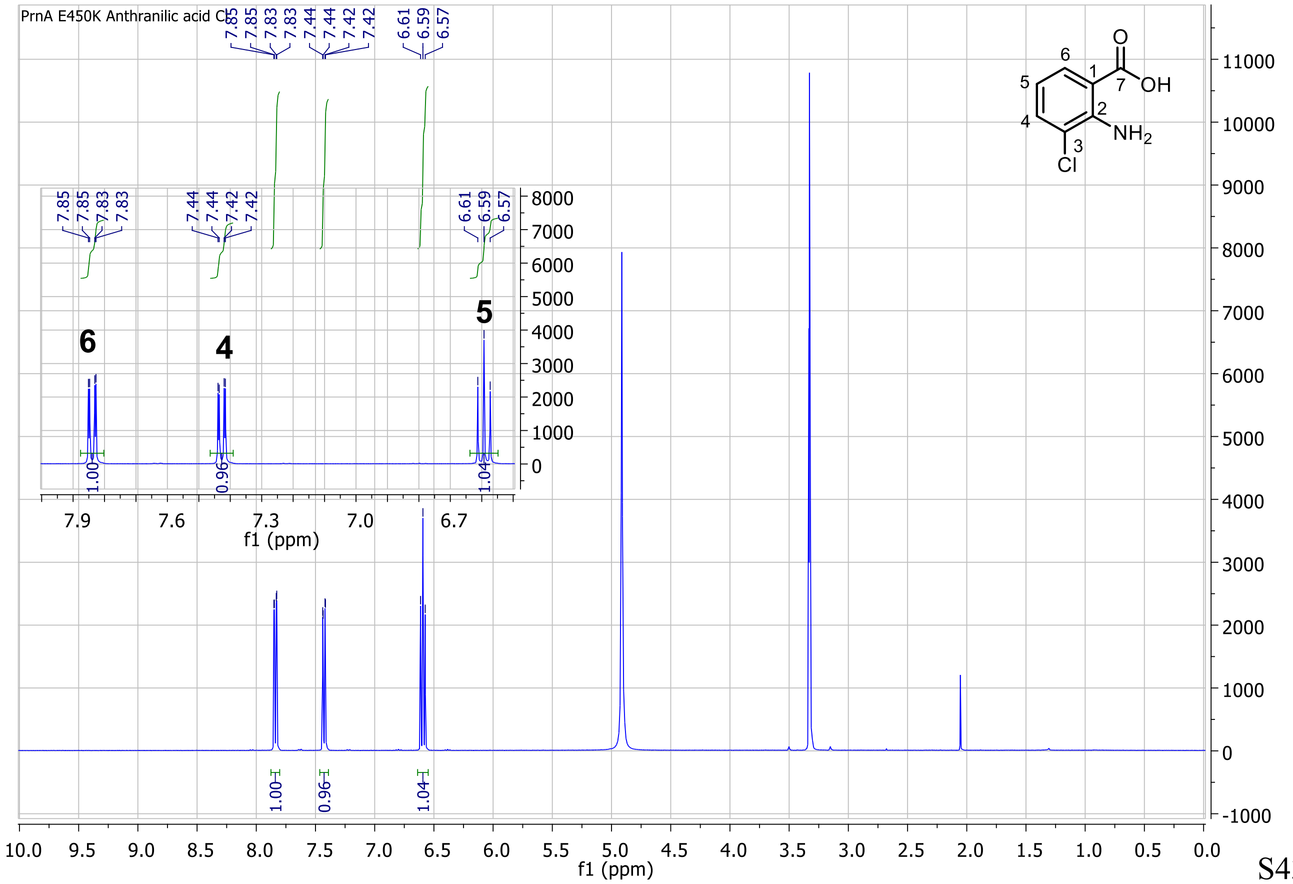
PrnA E450K Anthranilic acid Cl
6
4
5
f1 (ppm)
OH
NH2
Cl

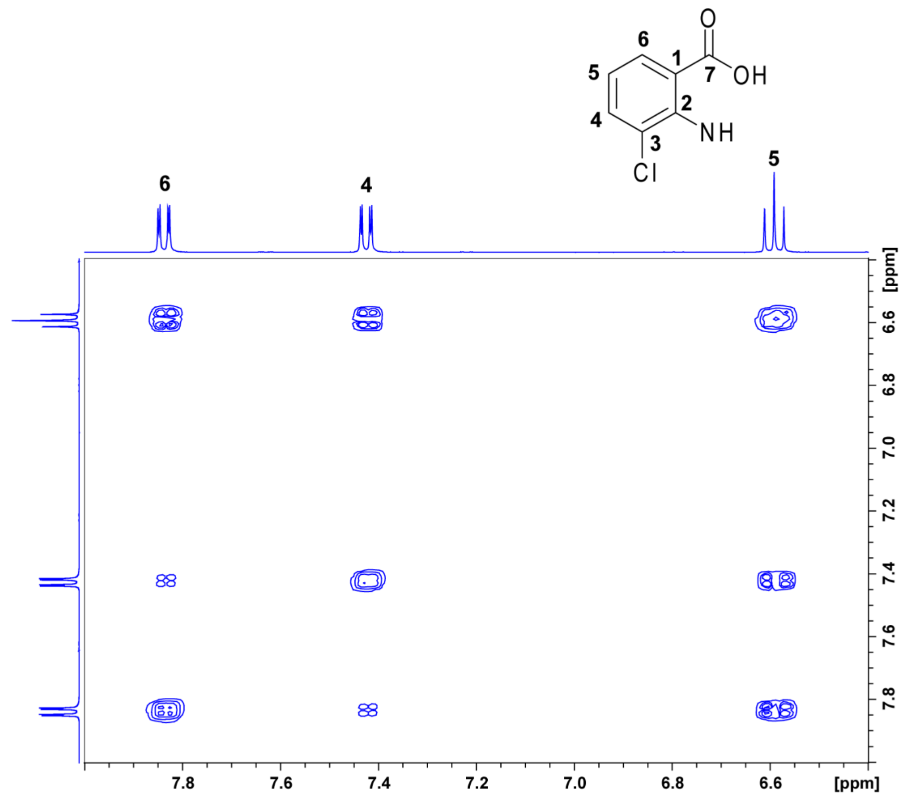

COSY of 3-chloroanthranilic acid

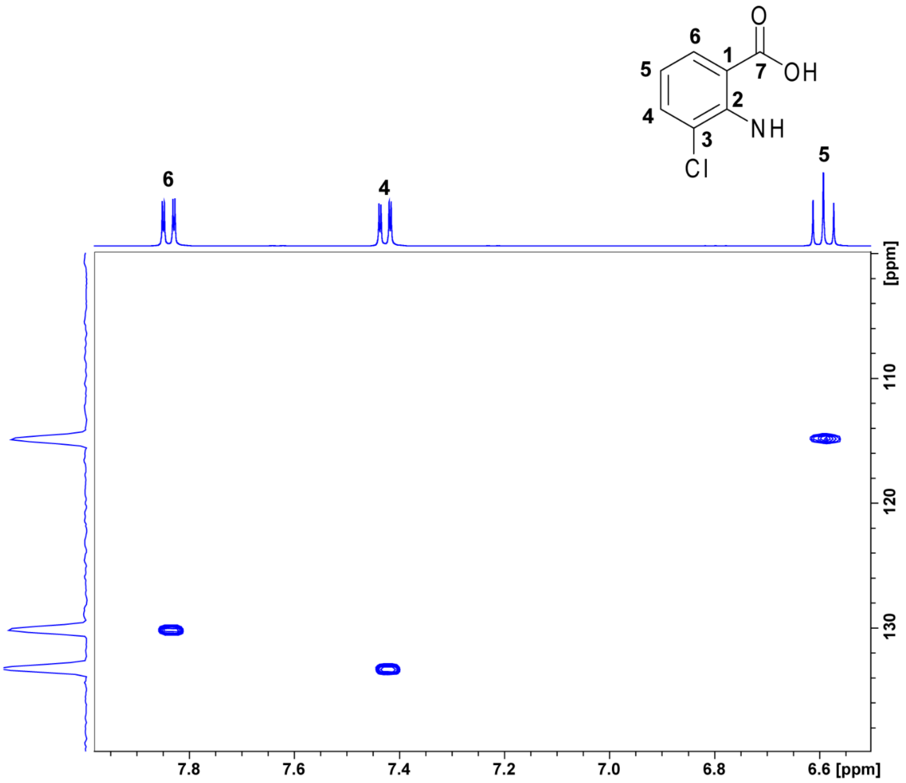

HSQC of 3-chloroanthranilic acid

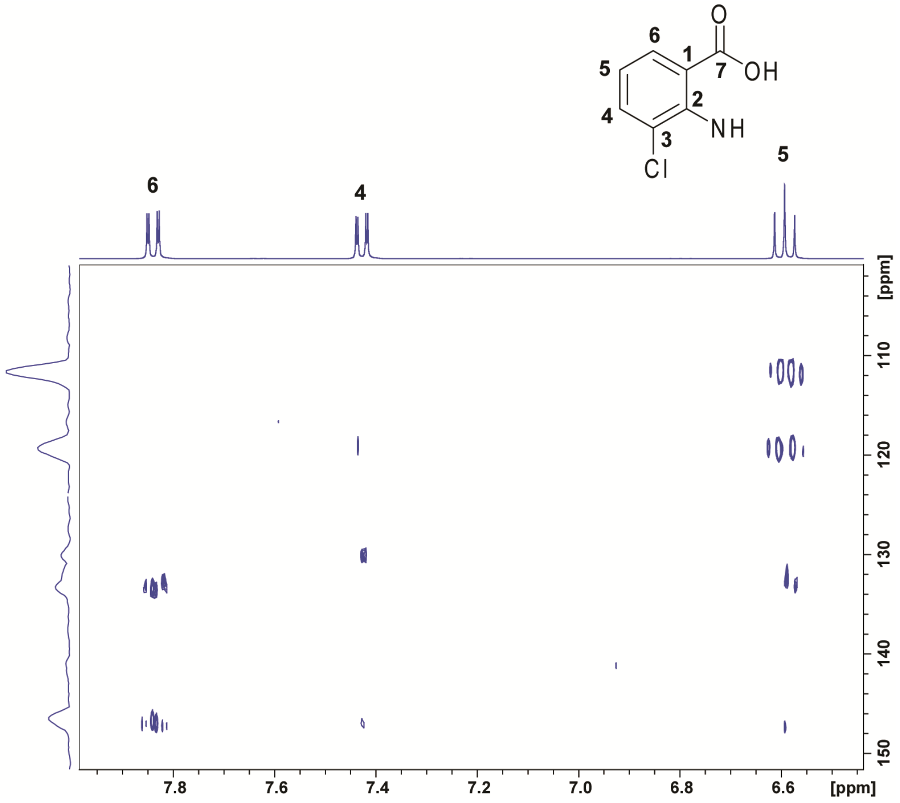

HMBC of 3-chloroanthranilic acid

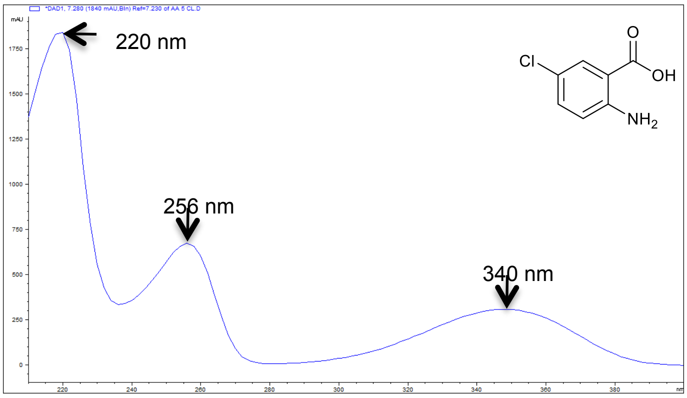

220 nm
256 nm
340 nm
Cl
O
OH
NH2

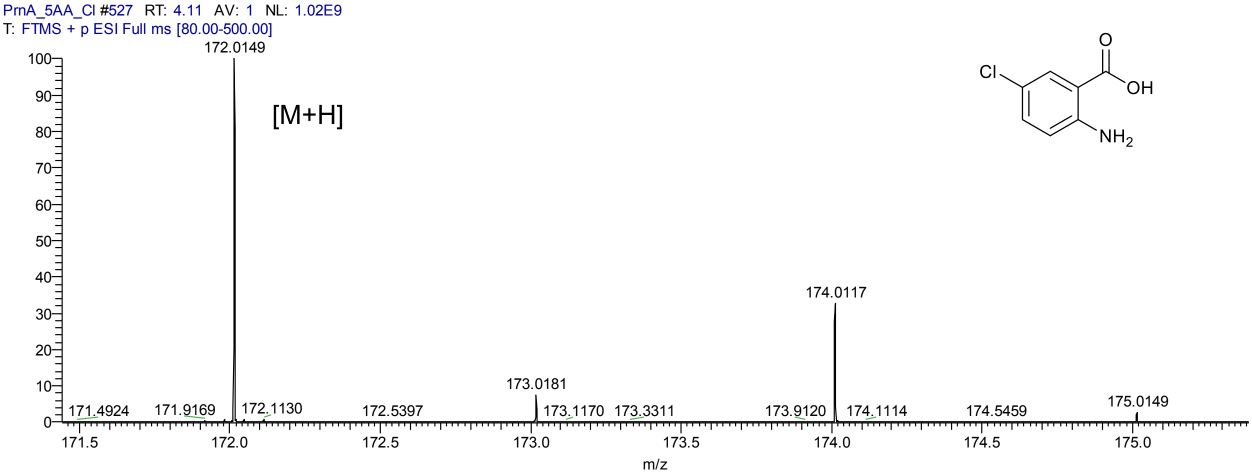

PmA_5AA_Cl #527 RT: 4.11 AV: 1 NL: 1.02E9
T: FTMS + p ESI Full ms [80.00-500.00]
172.0149
[M+H]
174.0117
173.0181
171.4924
171.9169
172.1130
172.5397
173.1170
173.3311
173.9120
174.1114
174.5459
175.0149
m/z
Cl
O
OH
NH2

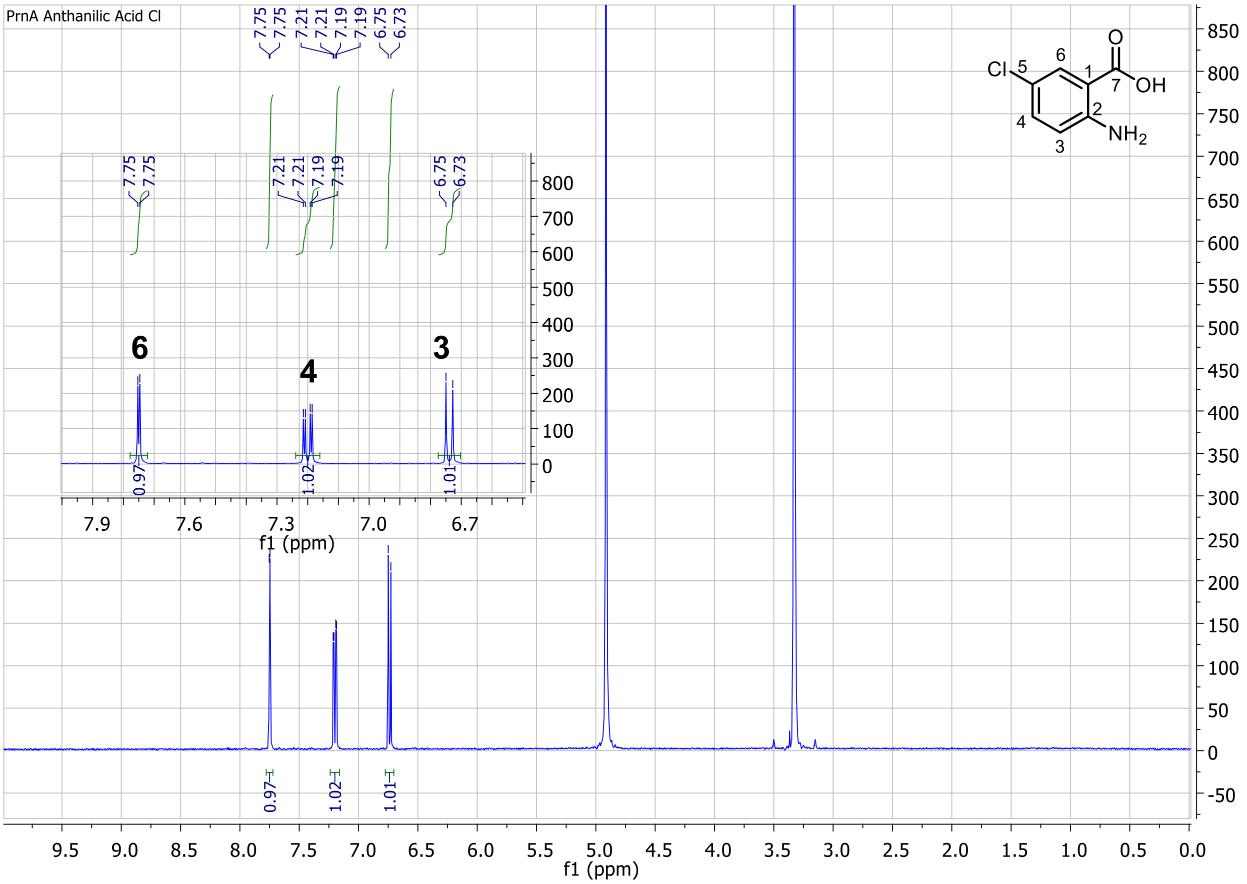

PrnA Anthanilic Acid Cl
7.75
7.75
7.21
7.21
7.19
7.19
6.75
6.73
6
4
3
0.97
1.02
1.01
f1 (ppm)
Cl
NH2
OH
O

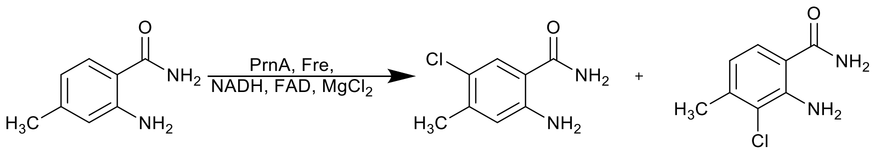
PrnA, Fre,
NADH, FAD, MgCl2

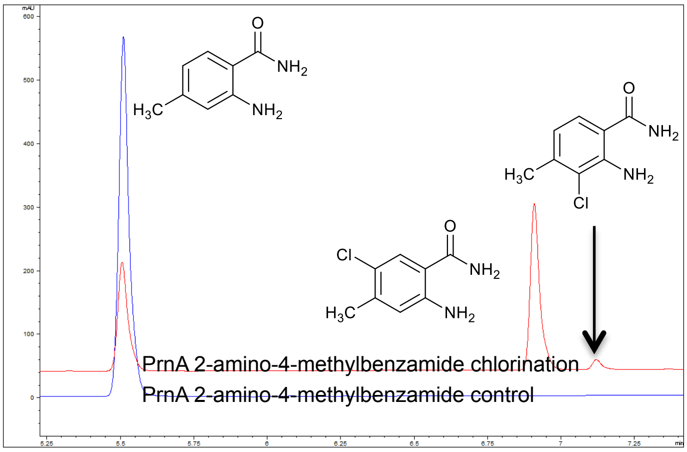
PrnA 2-amino-4-methylbenzamide chlorination
PrnA 2-amino-4-methylbenzamide control

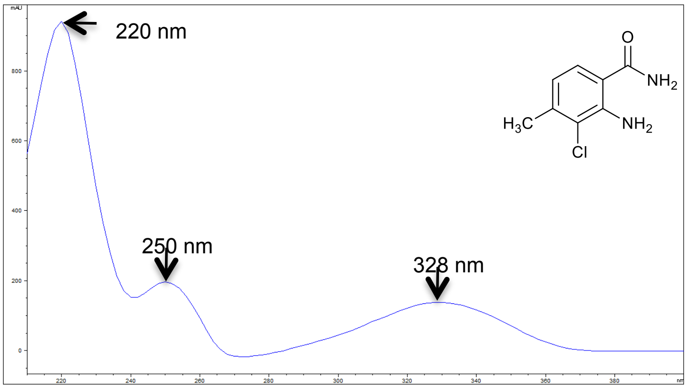
220 nm
250 nm
328 nm

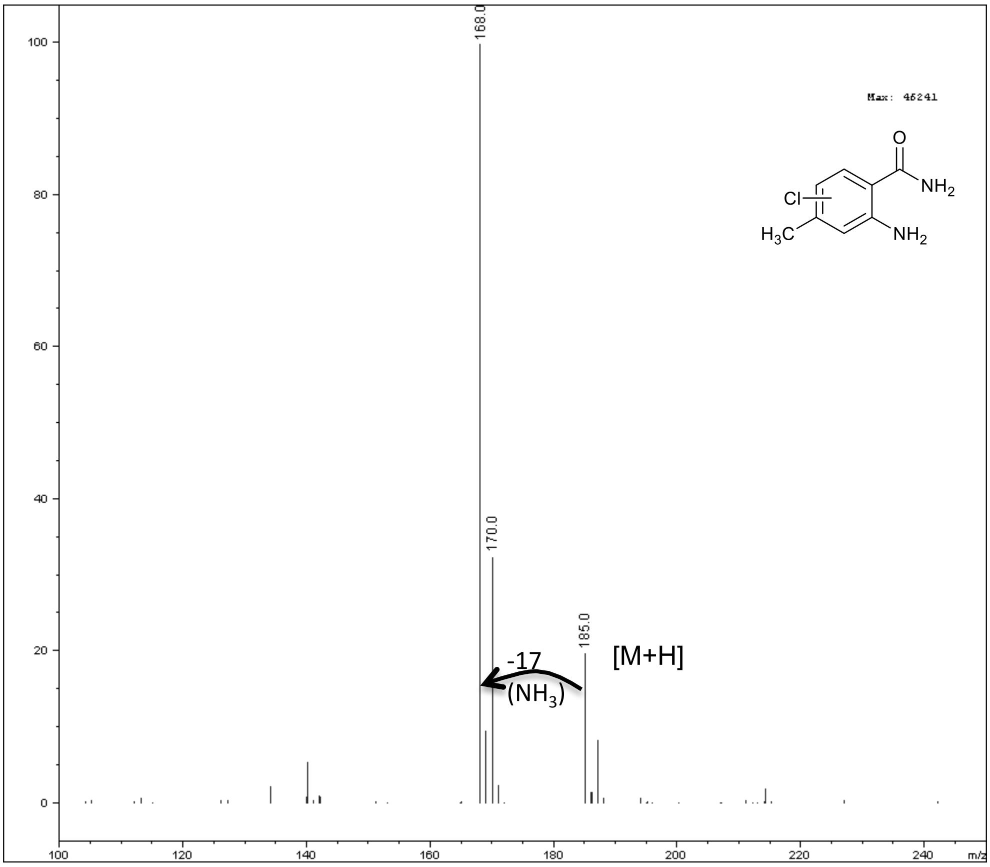
168.0
170.0
185.0
-17
(NH3)
[M+H]
Max: 46241
m/z

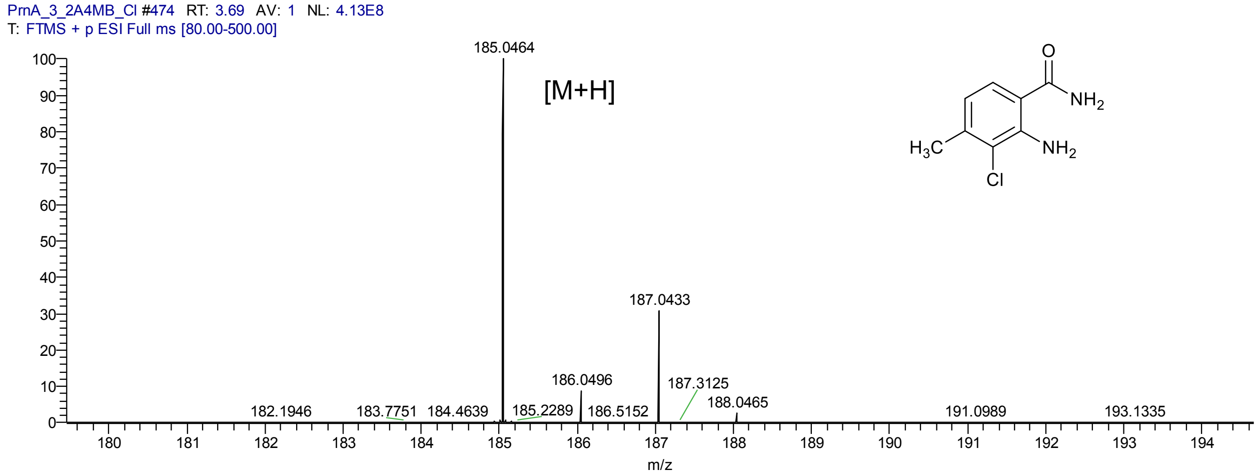

PrnA_3_2A4MB_Cl #474 RT: 3.69 AV: 1 NL: 4.13E8
T: FTMS + p ESI Full ms [80.00-500.00]
185.0464
[M+H]
187.0433
186.0496
187.3125
188.0465
182.1946
183.7751
184.4639
185.2289
186.5152
191.0989
193.1335
m/z
O
NH2
H3C
NH2
Cl

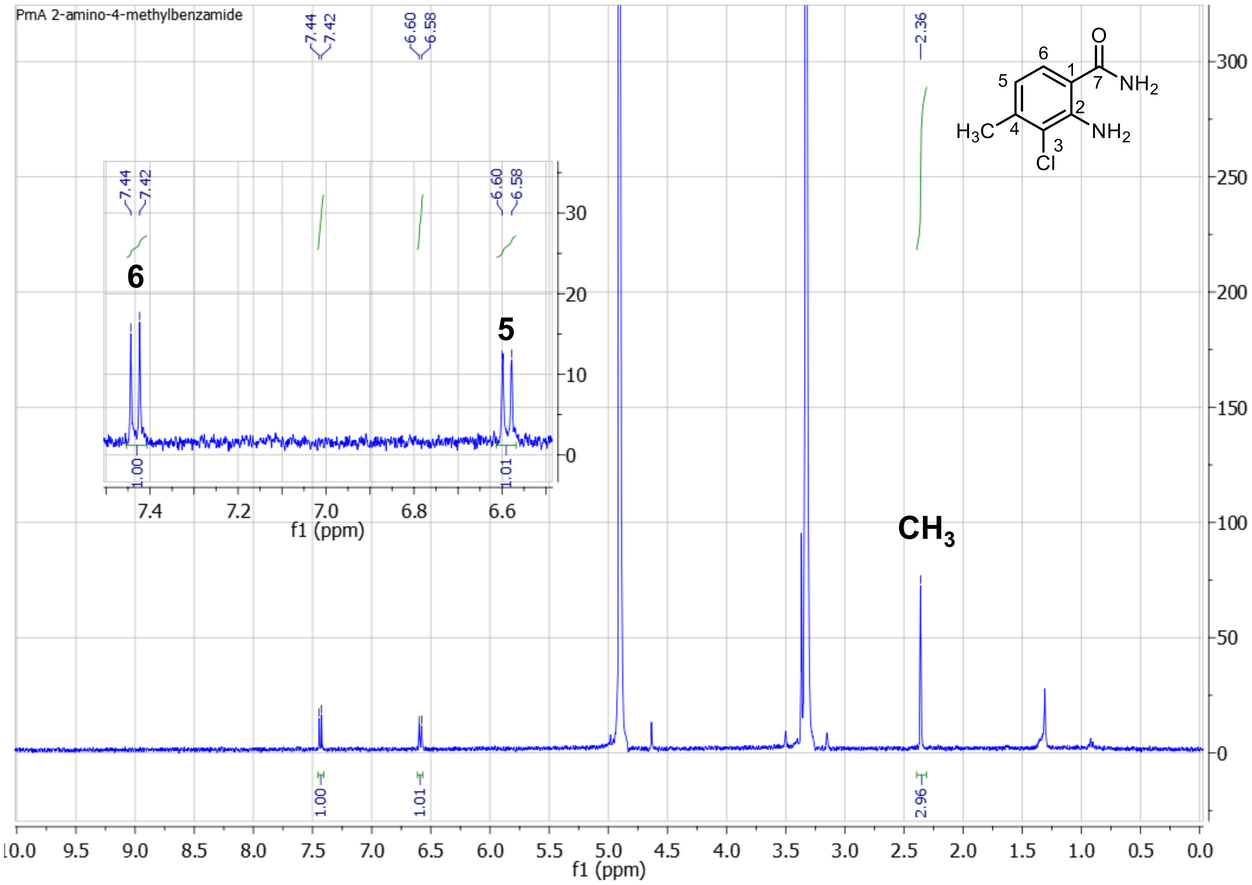

PrnA 2-amino-4-methylbenzamide
7.44
7.42
6.60
6.58
2.36
6
5
CH3
1.00
1.01
2.96
f1 (ppm)
O
NH2
NH2
H3C
Cl

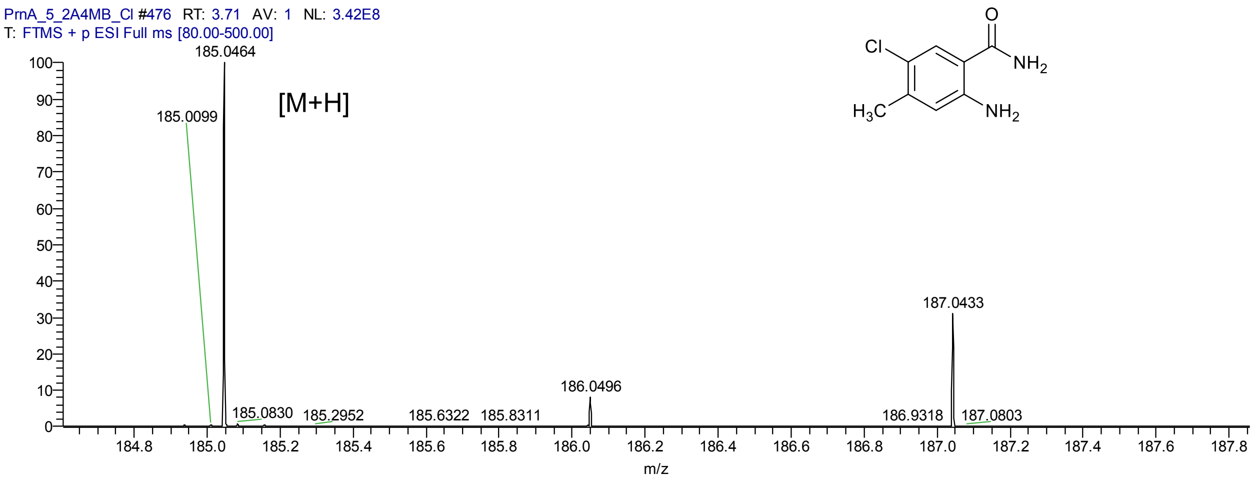
PmA_5_2A4MB_Cl #476 RT: 3.71 AV: 1 NL: 3.42E8
T: FTMS + p ESI Full ms [80.00-500.00]
[M+H]
185.0464
185.0099
185.0830
185.2952
185.6322
185.8311
186.0496
186.9318
187.0433
187.0803
m/z
Cl
O
NH2
H3C
NH2

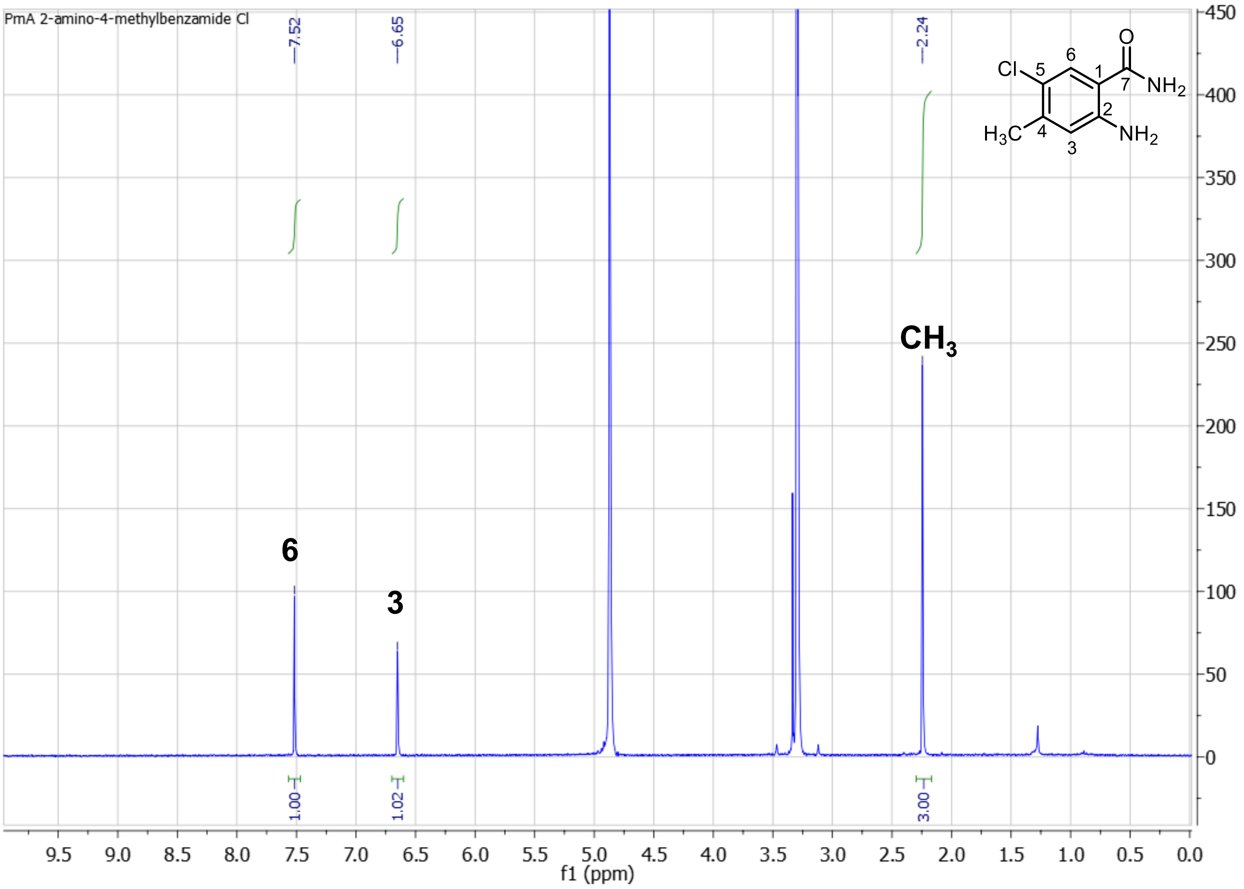
PmA 2-amino-4-methylbenzamide Cl
7.52
6.65
2.24
6
3
CH3
1.00
1.02
3.00
f1 (ppm)
Cl
5
6
1
7
O
NH2
2
H3C
4
3
NH2

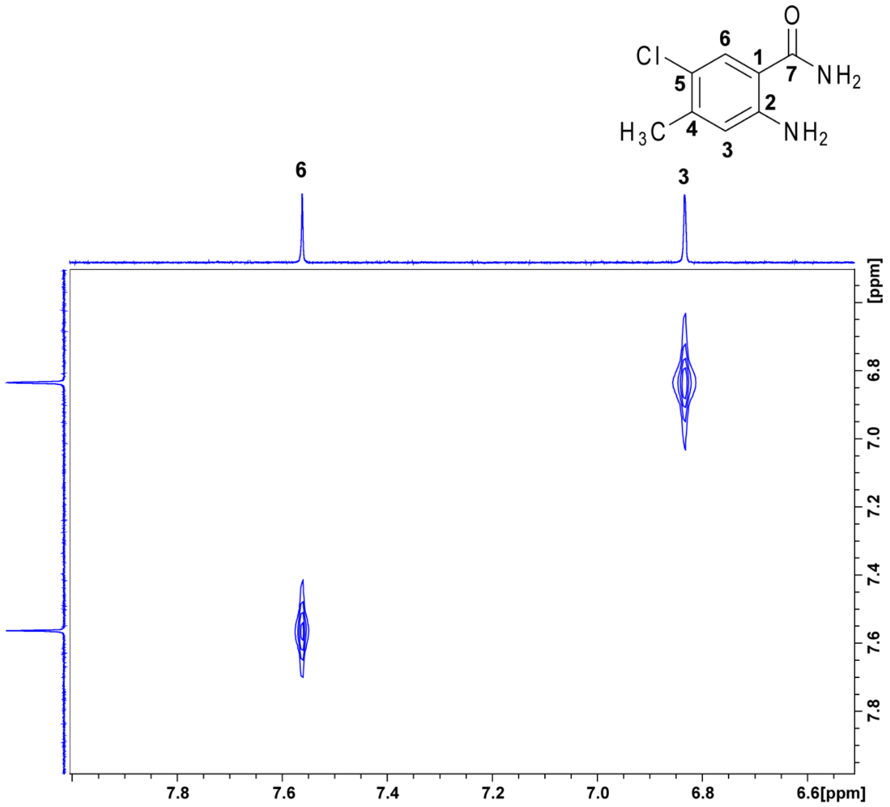

COSY of 2-amino-5-chloro-4-methylbenzamide

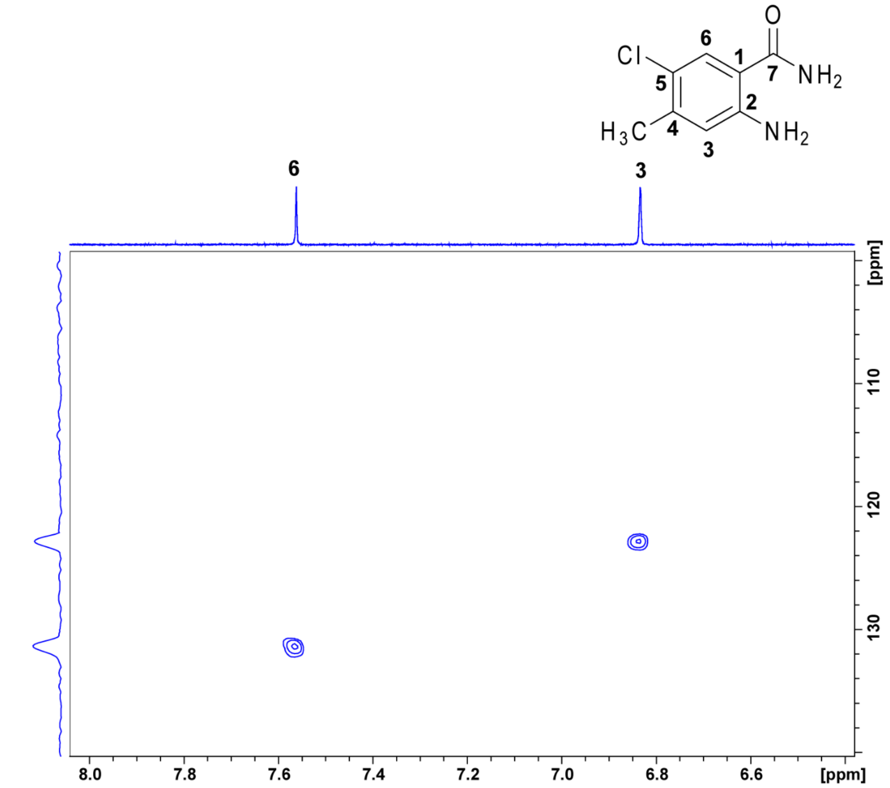

HSQC of 2-amino-5-chloro-4-methylbenzamide

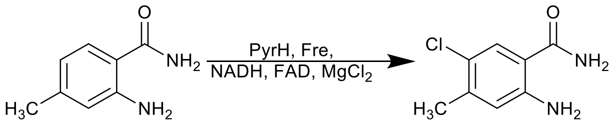
PyrH, Fre,
NADH, FAD, MgCl2

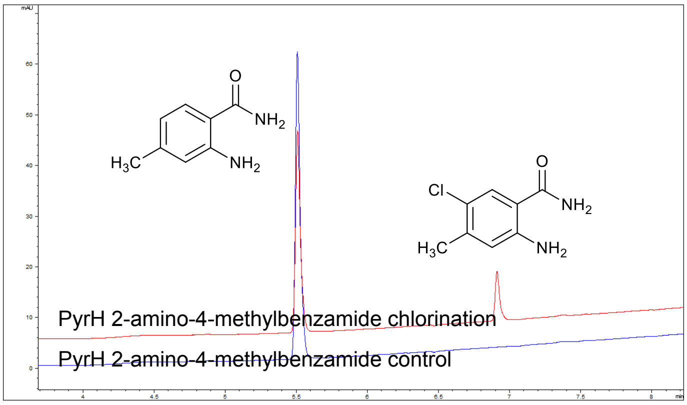
PyrH 2-amino-4-methylbenzamide chlorination
PyrH 2-amino-4-methylbenzamide control

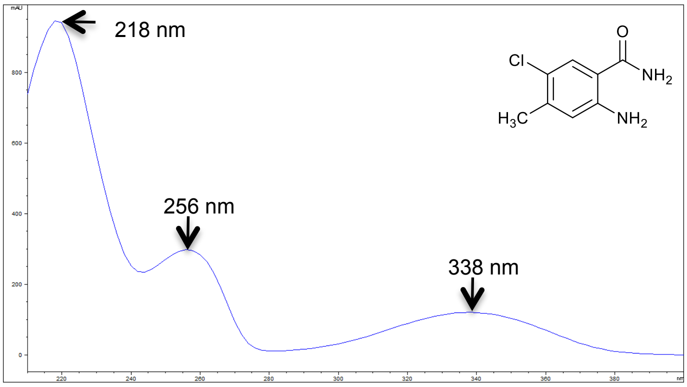
218 nm
256 nm
338 nm

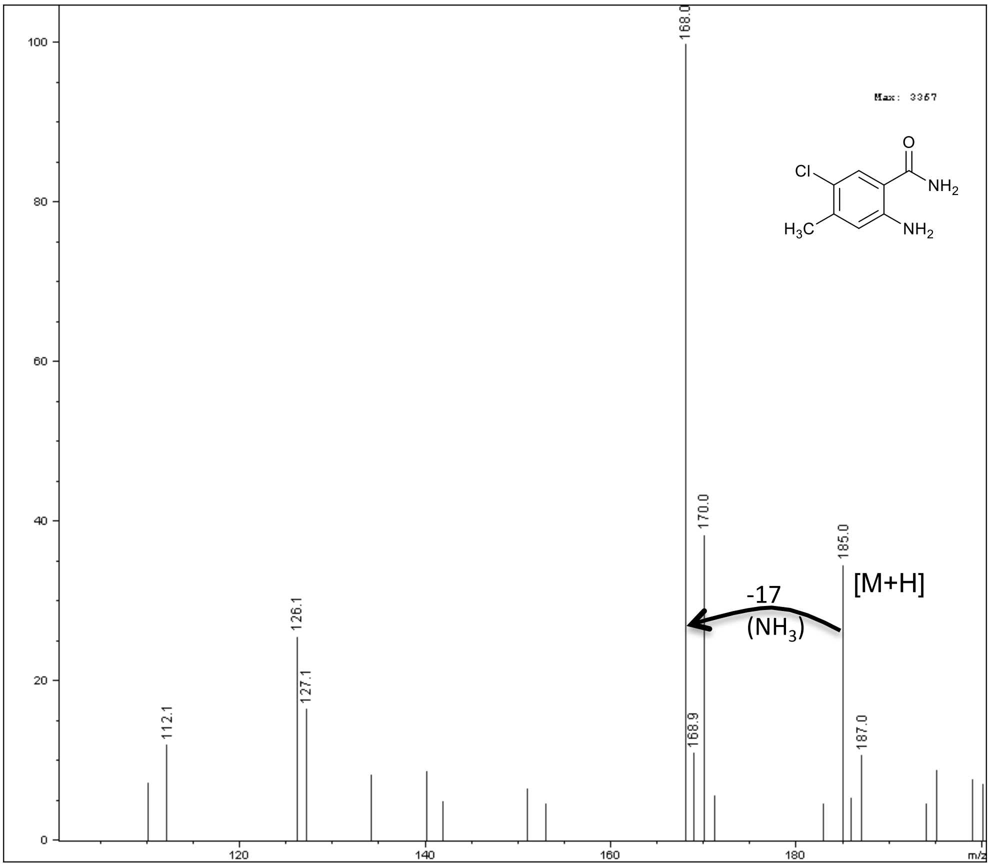
168.0
170.0
185.0
[M+H]
-17
(NH3)
126.1
127.1
112.1
168.9
187.0

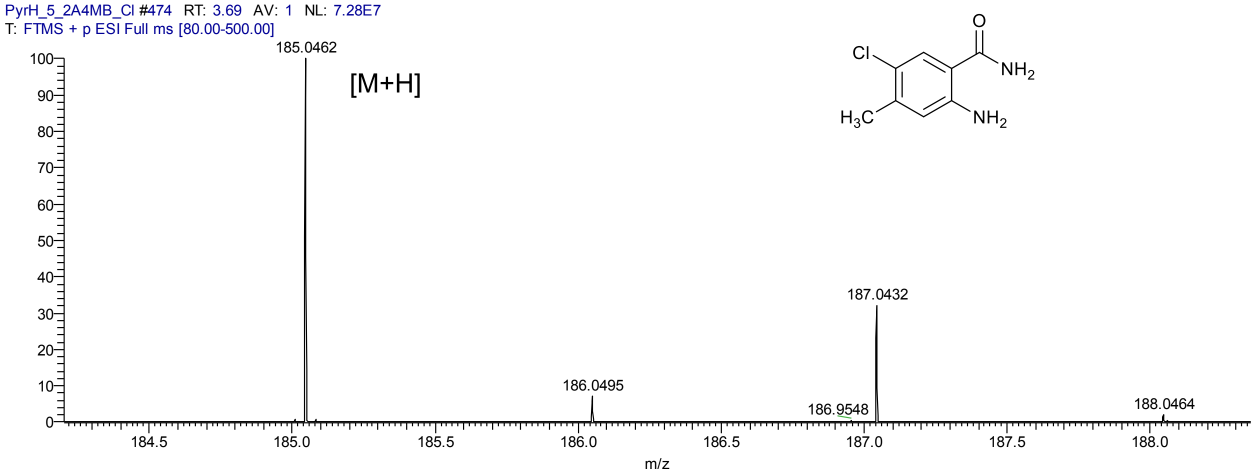
PyrH_5_2A4MB_Cl #474 RT: 3.69 AV: 1 NL: 7.28E7
T: FTMS + p ESI Full ms [80.00-500.00]
185.0462
[M+H]
187.0432
186.0495
186.9548
188.0464
m/z
Cl
O
NH2
H3C
NH2

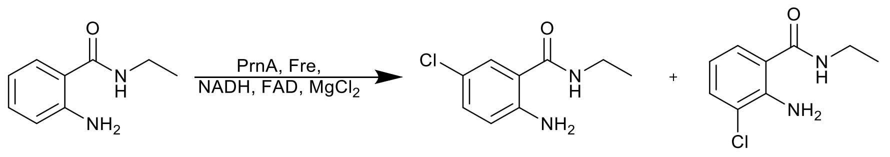
PrnA, Fre,
NADH, FAD, MgCl2
+

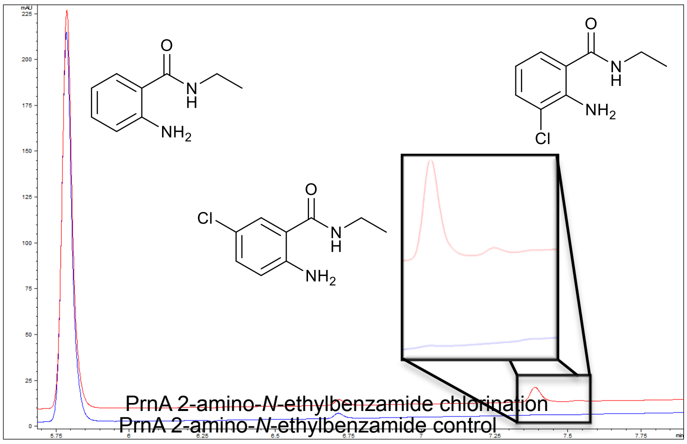
PrnA 2-amino-N-ethylbenzamide chlorination
PrnA 2-amino-N-ethylbenzamide control

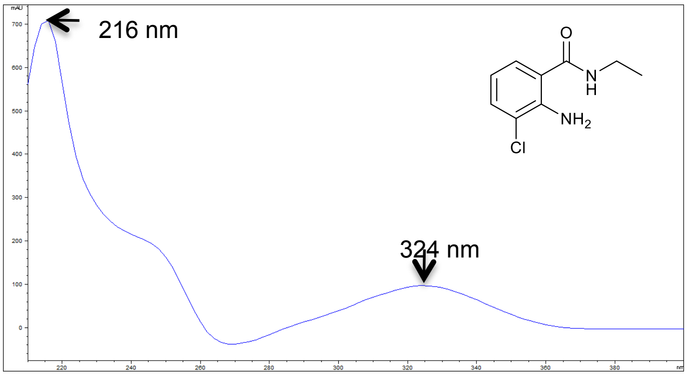
216 nm
324 nm

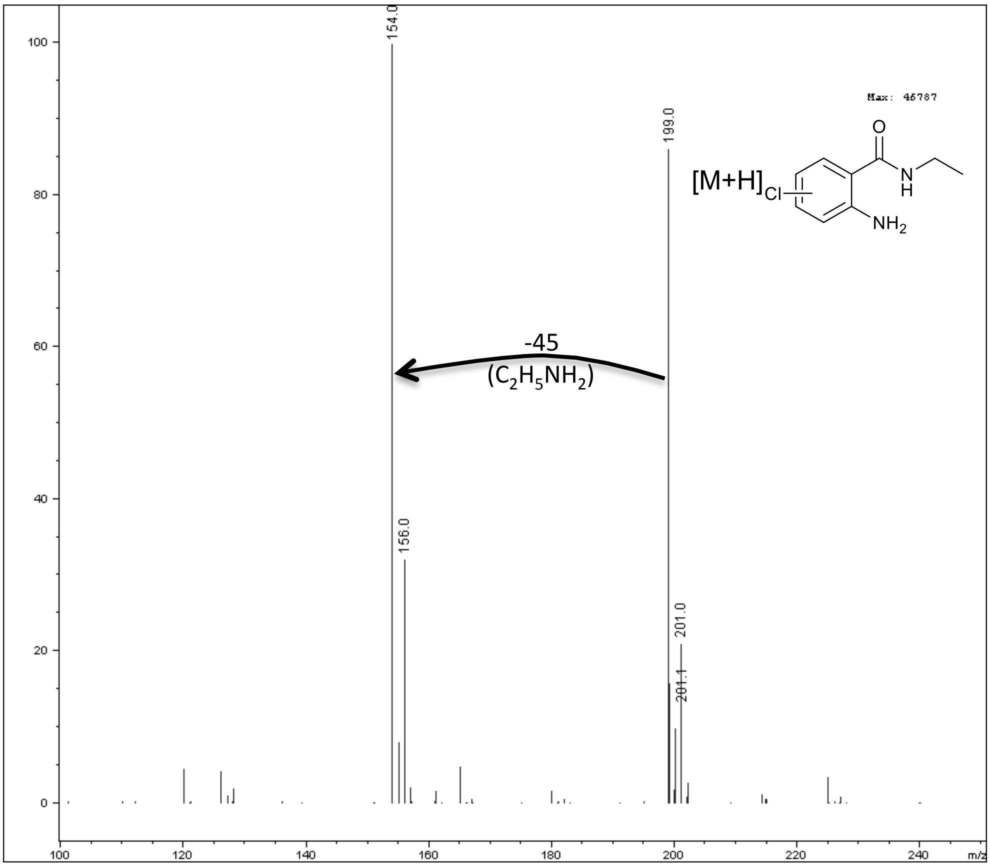
154.0
199.0
156.0
201.0
201.1
Max: 45787
[M+H]
-45
(C2H5NH2)
m/z

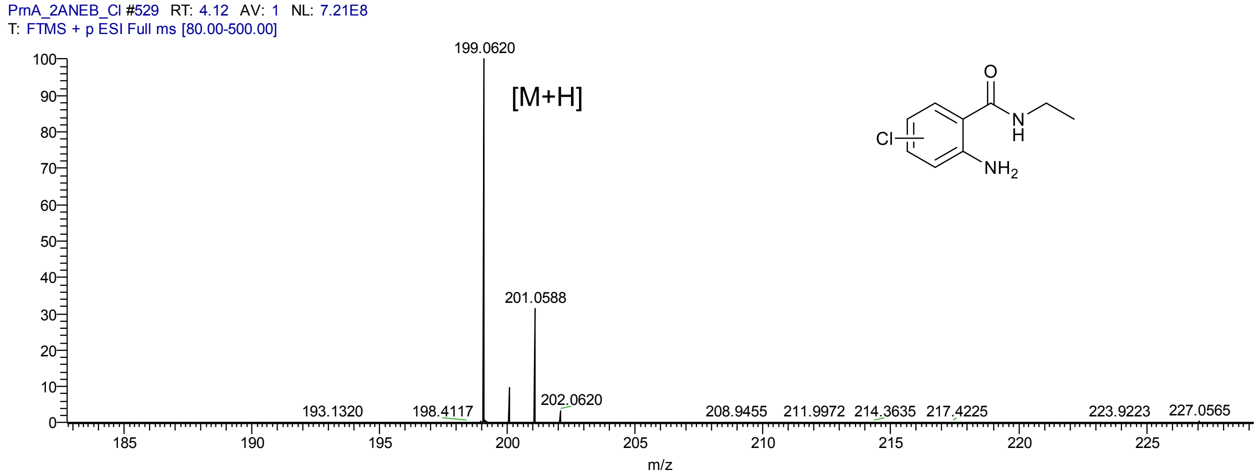
PmA_2ANEB_Cl #529 RT: 4.12 AV: 1 NL: 7.21E8
T: FTMS + p ESI Full ms [80.00-500.00]
199.0620
[M+H]
201.0588
193.1320
198.4117
202.0620
208.9455
211.9972
214.3635
217.4225
223.9223
227.0565
m/z
Cl
O
N
H
NH2

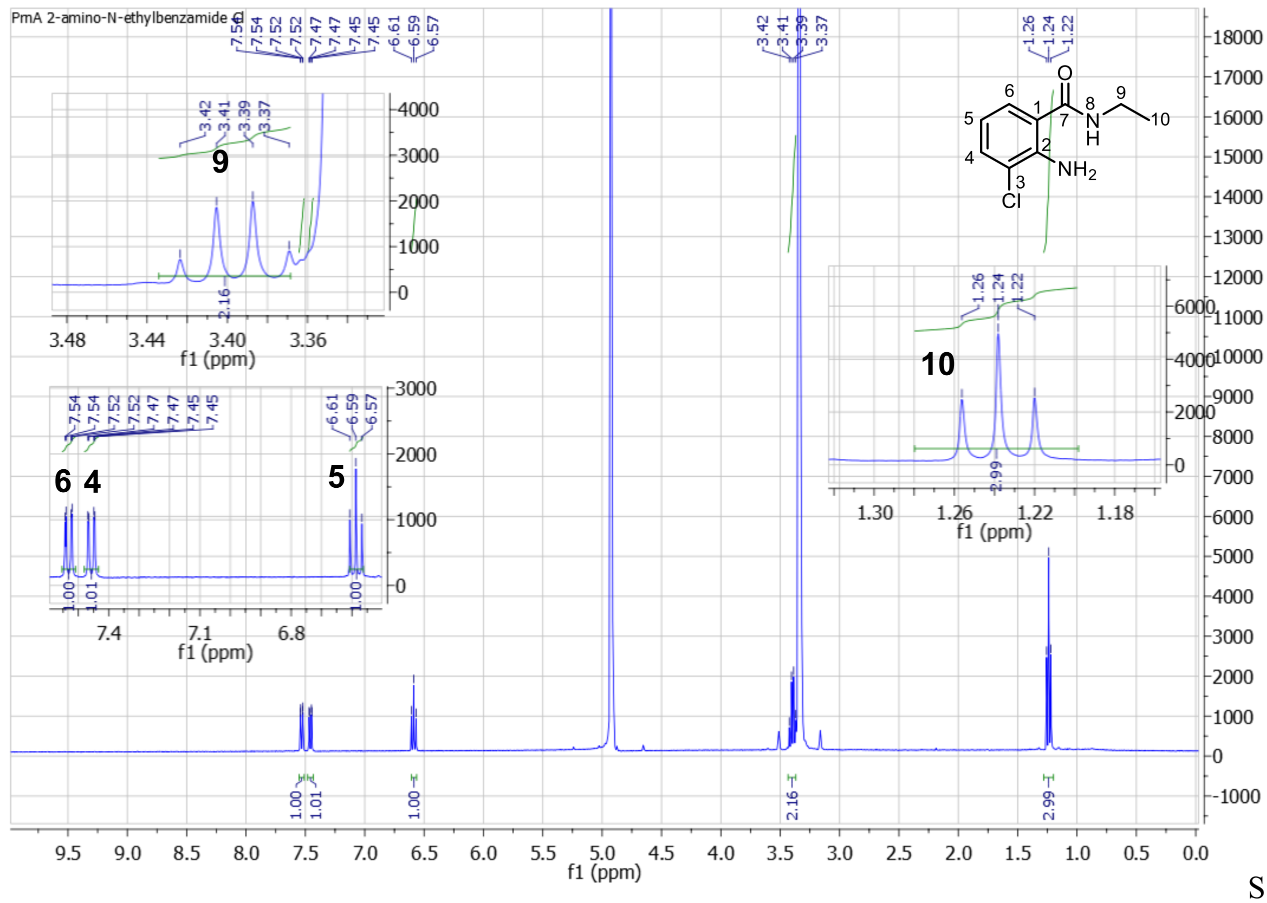
PmA 2-amino-N-ethylbenzamide Cl
9
6 4
5
10
f1 (ppm)
NH2
Cl

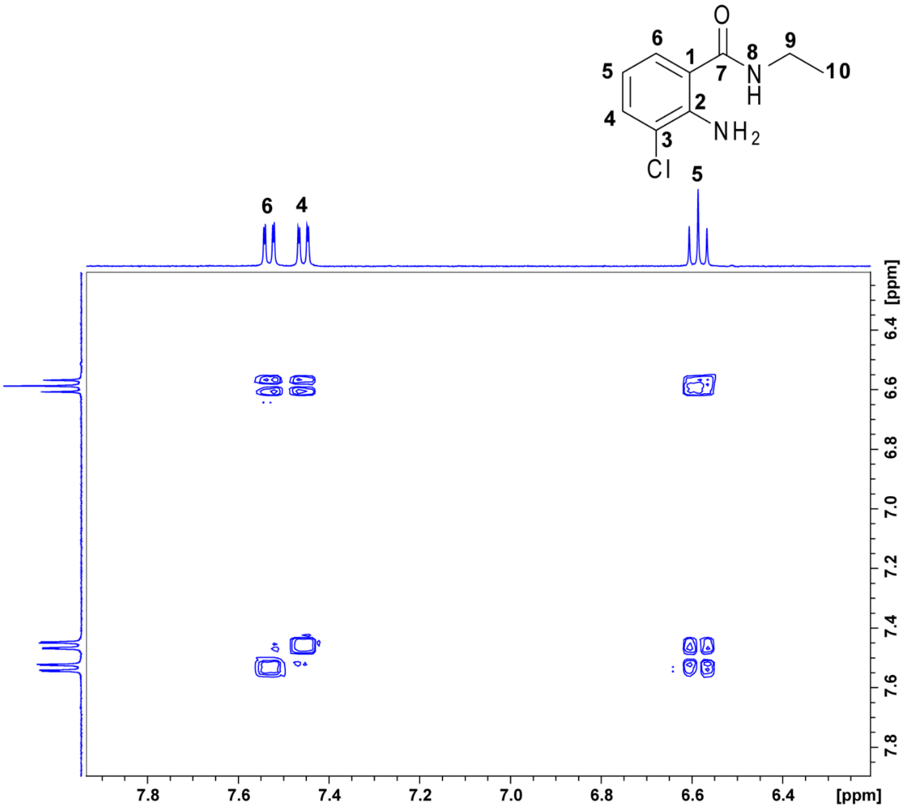

COSY of 2-amino-3-chloro-N-ethylbenzamide

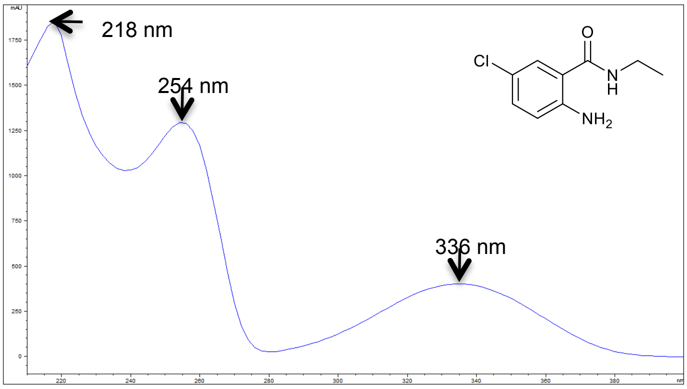
218 nm
254 nm
336 nm

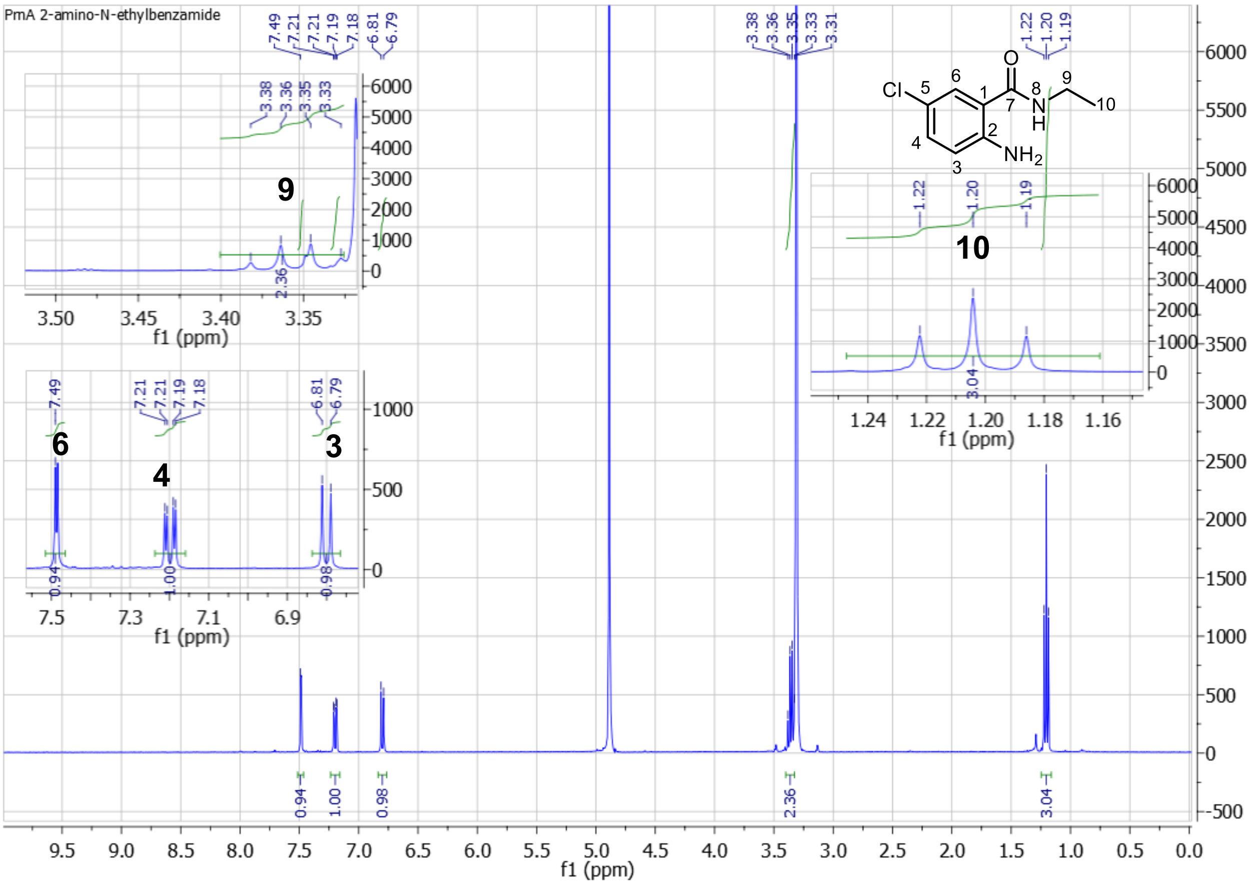
PmA 2-amino-N-ethylbenzamide
6
4
3
9
10
f1 (ppm)

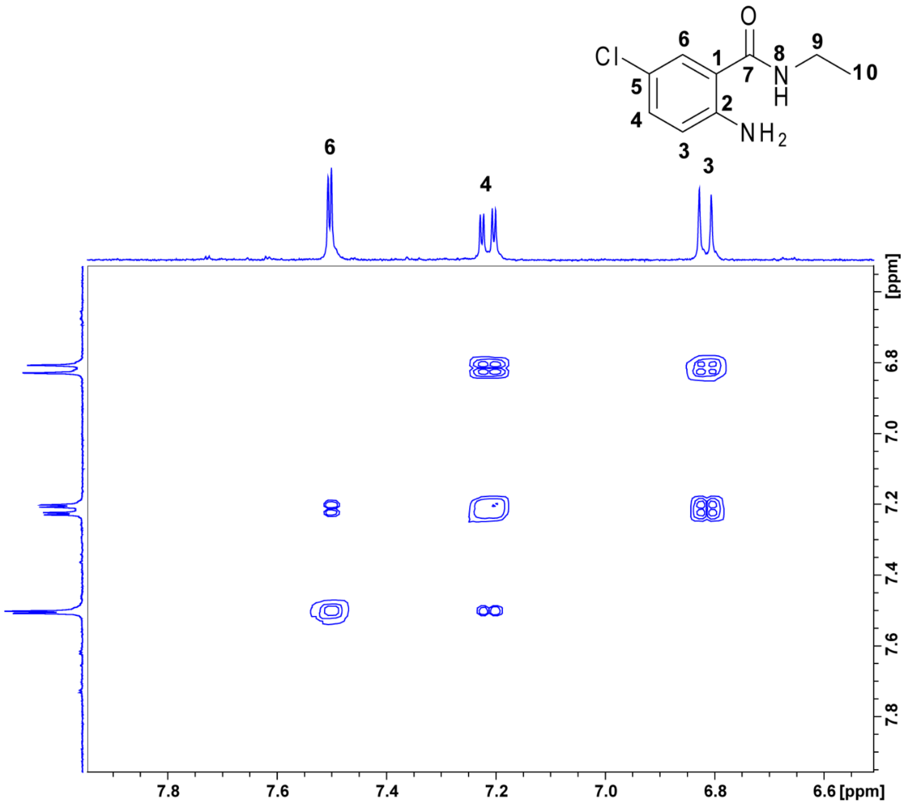

COSY of 2-amino-5-chloro-N-ethylbenzamide

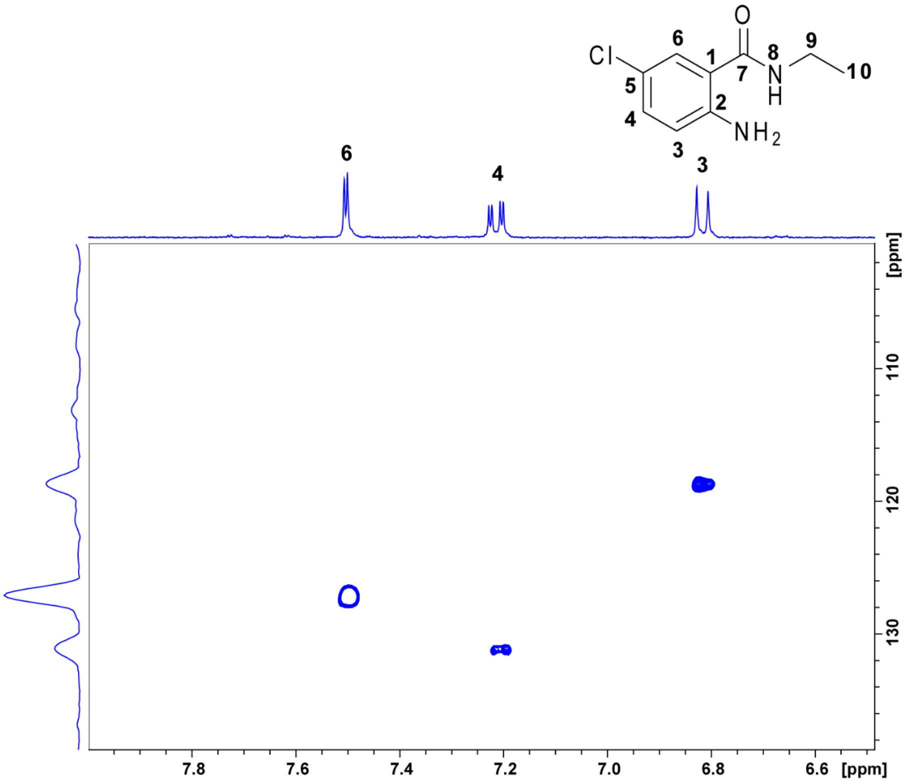

HMQC of 2-amino-5-chloro-N-ethylbenzamide

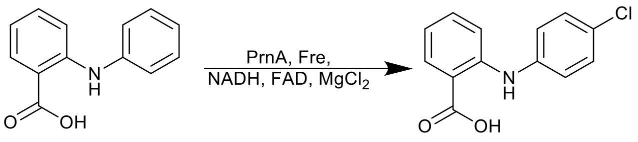
PrnA, Fre,
NADH, FAD, $MgCl_2$
N
H
O
OH
Cl

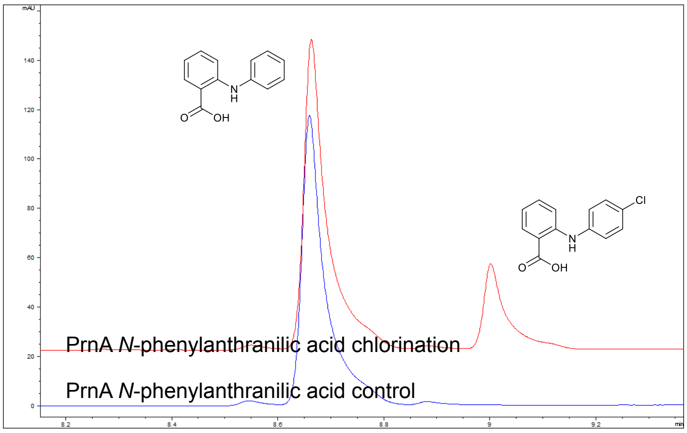
PrnA *N*-phenylanthranilic acid chlorination
PrnA *N*-phenylanthranilic acid control

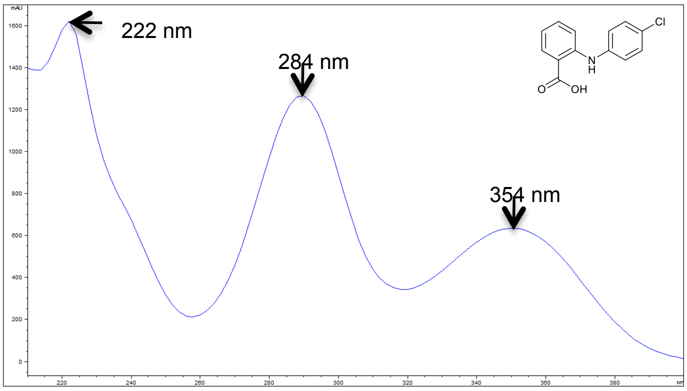
222 nm
284 nm
354 nm

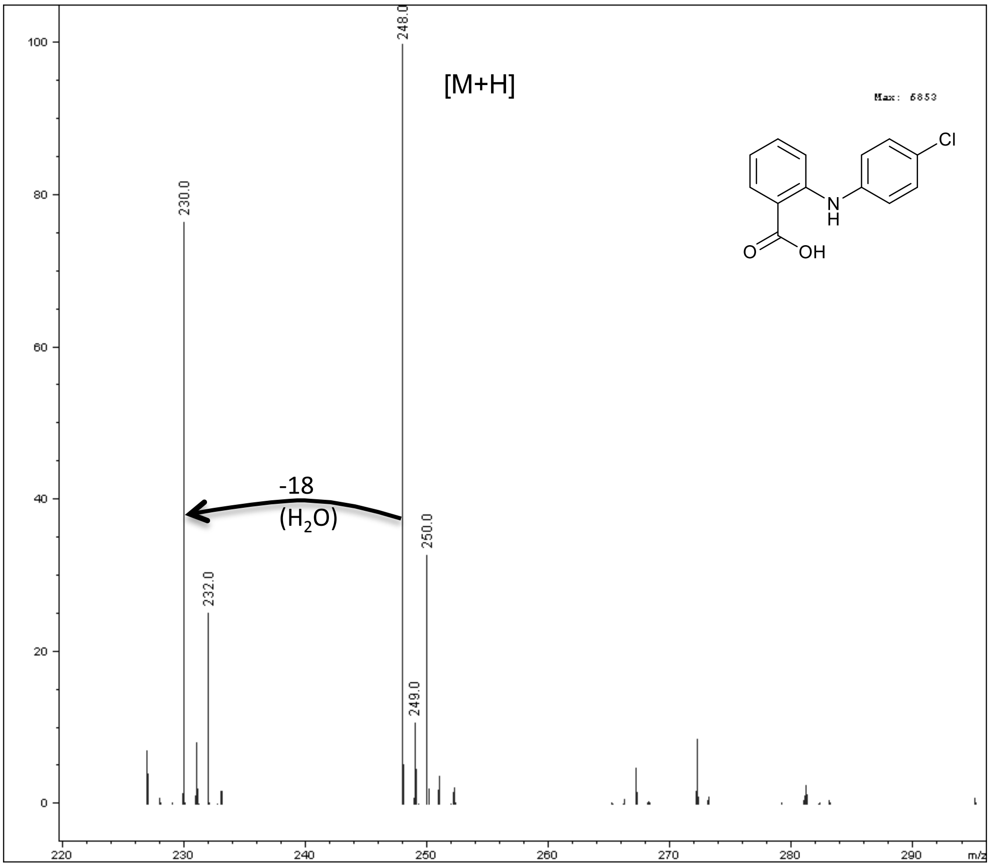
[M+H]
Max: 6853
248.0
230.0
250.0
232.0
249.0
-18
(H_2O)
m/z

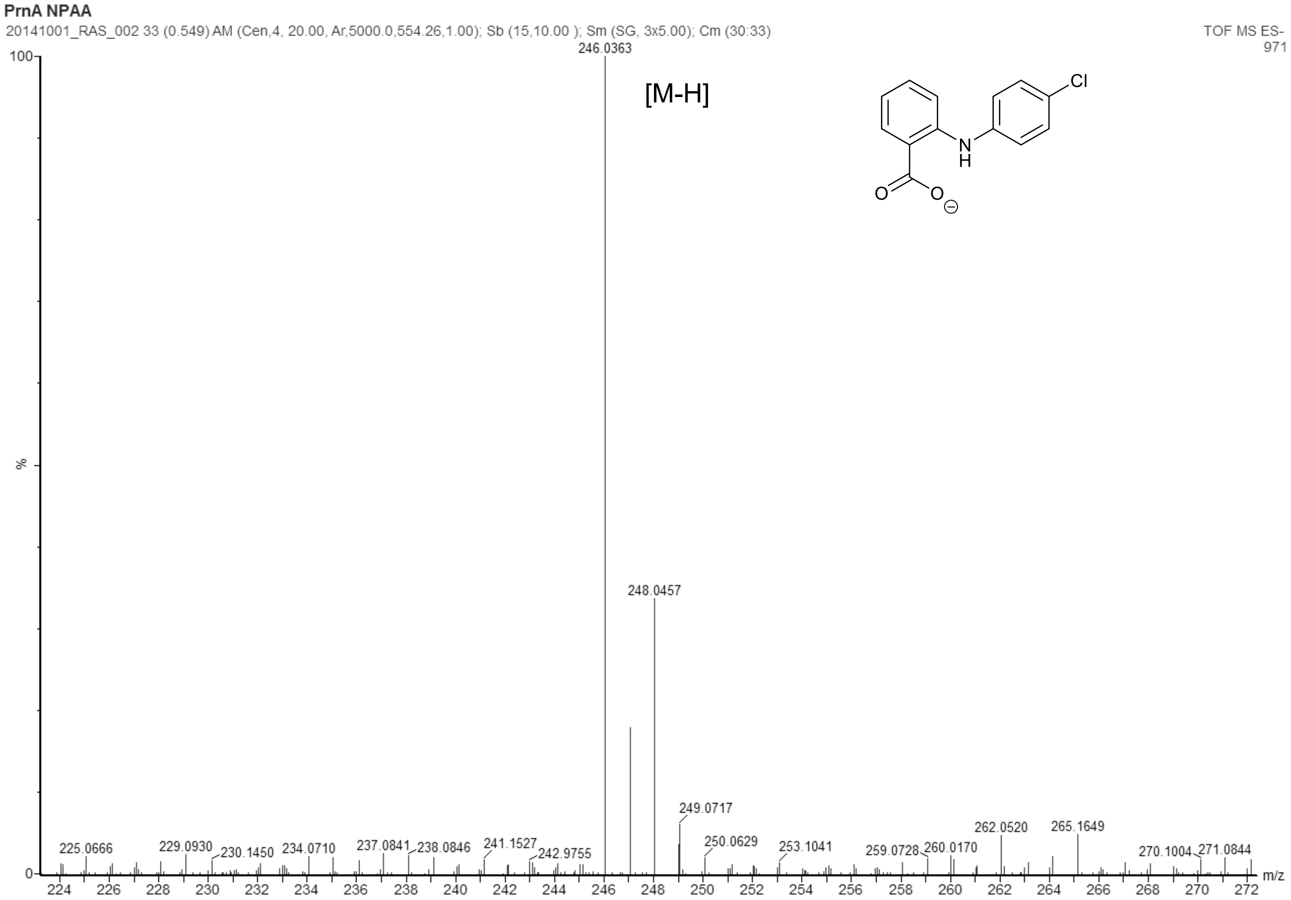
PrnA NPAA
20141001_RAS_002 33 (0.549) AM (Cen,4, 20.00, Ar,5000.0,554.26,1.00); Sb (15,10.00); Sm (SG, 3x5.00); Cm (30:33)
TOF MS ES-
971
246.0363
[M-H]
248.0457
249.0717
262.0520
265.1649
Cl
N
H
O
O
%
m/z

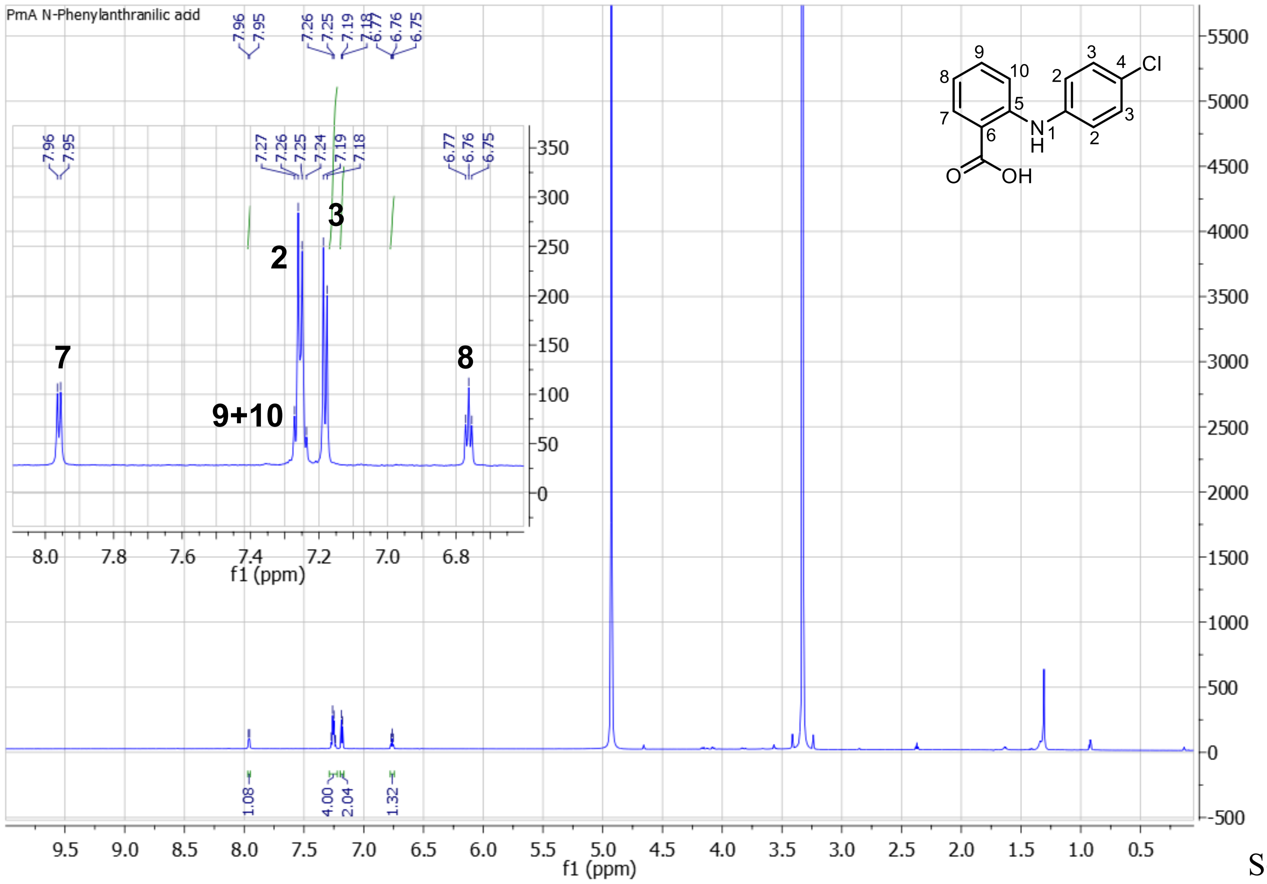
PrnA N-Phenylanthranilic acid
7
2
3
8
9+10
1.08
4.00
2.04
1.32
f1 (ppm)
Cl
OH
O

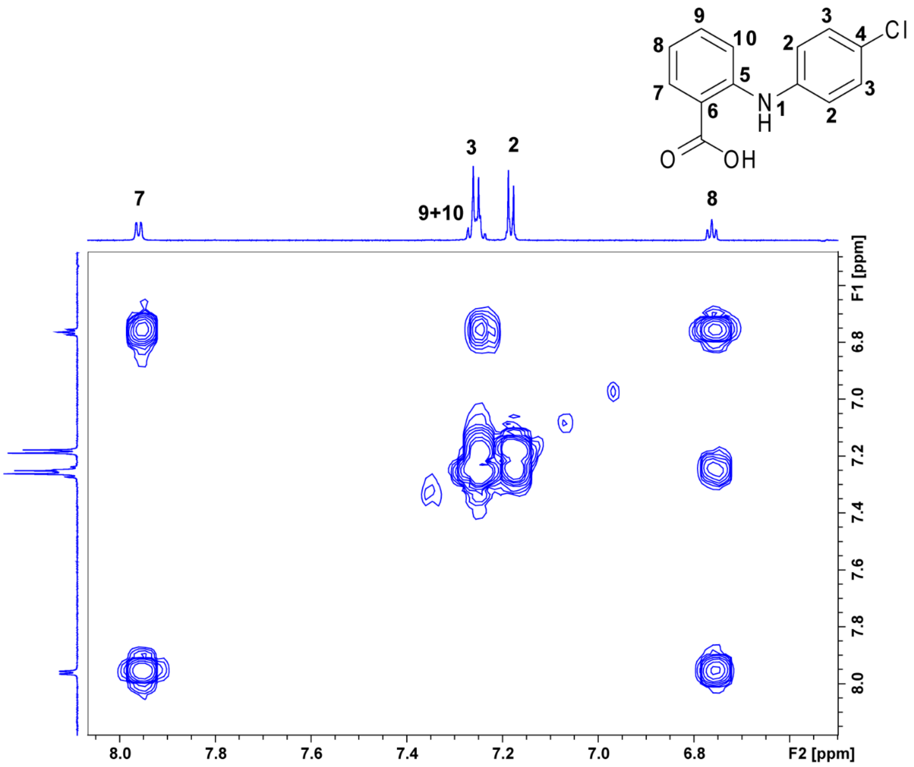

COSY of *N*-(4-chlorophenyl)anthranilic acid

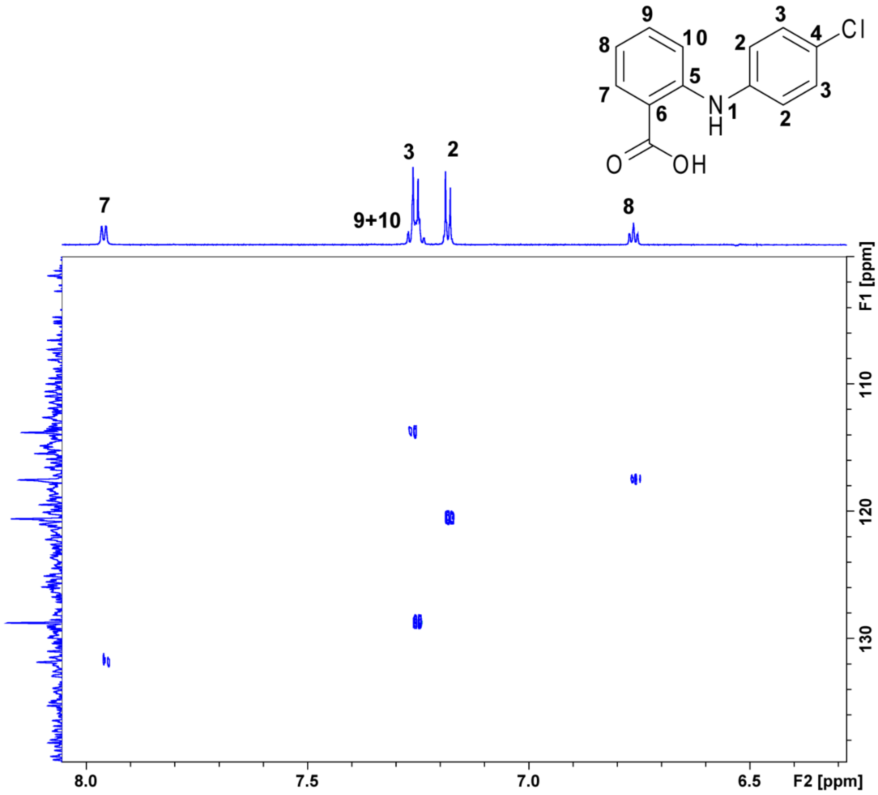

HSQC of N-(4-chlorophenyl)anthranilic acid

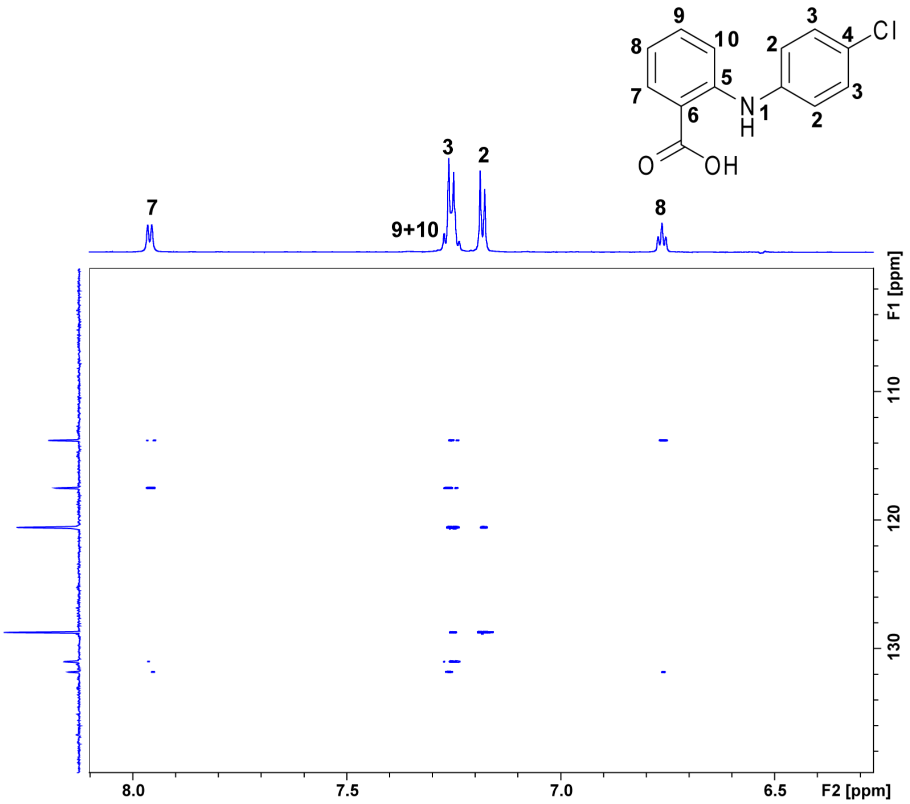

HSQC TOCSY of N-(4-chlorophenyl)anthranilic acid
